# Abstracts of papers presented at the 1998 Pittsburgh Conference

**DOI:** 10.1155/S1463924698000200

**Published:** 1998


Journal of Automatic Chemistry, Vol. 20, No. 5 (September–October 1998) pp. 131–175

### Abstracts of papers presented at the 1998 Pittsburgh Conference

The following abstracts are of papers presented at this year's Pittcon held in March in New Orleans, LA. The editors have selected, from over 1000 presentations, those of particular interest to the *Journal of Automatic Chemistry*'s readers. For information on next year's Pittcon, contact The Pittsburgh Conference, 300 Penn Center Boulevard, Suite 332, Pittsburgh, PA 15235–5503, USA. Tel.: 412 825 3220; fax.: 412 825 3224; World Wide Web: http://www.pittcon.org.

### Microchips for the evaluation of cell processes and cell-drug interactions

*D. Jed Harrison[1], Per E. Andersson[1], Rod Szarka[2], Hossein Salimi-Moosavi[1], Nghia Chiem[1], Charmaine X. Qiu[1], Said Attiya[1], Richard Smith[1] and William Lee[3], [1]Department of Chemistry, University of Alberta, Edmonton, AB, Canada T6G2G2; [2]Alberta Research Council, Edmonton, AB, Canada T6H5X2; [3]Defense Research Establishment, Suffield*

Microchip-based fluidic devices may provide a convenient means to automate sample preparation and assays outside of the laboratory environment. Two application areas that could benefit significantly from the capabilities of these devices are immunoassays and bioassays. The latter term is meant to encompass assays based on monitoring of a biological cell following a chemical or biochemical insult. In particular, single cell assays could benefit from the ease of fluid manipulation within the microchip format.

Bioassays based on cells are well established. Microfluidic devices will allow the study of chemical interactions with single cells, and should prove relevant to both performing assays and drug screening. The devices we have developed consume nl of sample per test, allow precise location of the cells and reagents within a few μm at the time of mixing, provide an estimate of the initial time of mixing to within 0.25 s, precisely define the reagent concentration upon mixing, and allow observation of the non-adherent cell both before and after mixing with a reagent. Multiple reagents can be added at different stages, so that incubation times can be varied. While targeted at single cell analysis, it should be possible to make the device sufficiently parallel to analyse up to several hundred cells at once. Imaging of the cells provides enough information to allow rejection of unsuitable cells before data analysis.

### Development of a CE-EC method employing on-line derivatization with electrogenerated bromine

*Lisa A. Holland and Susan M. Lunite, Department of Pharmaceutical Chemistry and Center for Bioanalytical Research, The University of Kansas, 2095 Constant Ave., Lawrence, KS 66047, USA*

Glutathione is known to be involved in the metabolism and detoxification of many xenobiotics, including drugs, carcinogens and environmental toxins. Xenobiotics are eliminated from the body as acetylcysteine conjugates. Thus, detection of glutathione, cysteine and acetylcysteine conjugates would be useful for metabolism studies. Glutathione and cysteine conjugates have previously been analysed by HPLC coupled to electrochemical detection with the use of on-line bromine generation. The basis of on-line bromine electrogeneration is the production of bromine from bromide at a generating electrode. The generated bromine then reacts with the analyte of interest and the signal at a second electrode, which normally yields a signal from the reduction of bromine to bromide, is diminished according to the amount of bromine that has reacted with the analyte.

The benefits of using CE-EC with a bromine electrogeneration method rather than HPLC-EC are that CE-EC provides more flexibility in the choice of separation and generation pH, and the separation is more efficient. To date, no work with CE-EC electrogeneration of bromine has been reported. Therefore, such a method has been investigated for use as sensitive technique applicable to the study of the metabolism of a variety of pharmaceuticals including cancer drugs.

### Diagnostic sequencing of exons 5–8 of the p53 gene by mass spectrometry

*Kai Tang, Dong-Jing Fu, Andreas Braun, Daniel Little, Maryanne O'Donnell, Dirk Reuter, Brigitte Darnhofer, Charles Cantor[1] and Hubert Köster, Sequenom, 11555 Sorrento Valley Rd, San Diego, CA 92121, USA; [1]Boston University, Center for Advanced Biotechnology, 36 Cummington St., Boston, MA 02155, USA*

We presented here for the first time information that a biologically relevant human gene has been sequenced using matrix-assisted laser desorption/ionization (MALDI) time-of-flight mass spectrometry (TOFMS). Molecular weight, the intrinsic property of a molecule, is detected in MALDI-TOFMS rather than ion mobility in gel media in electrophoresis. This makes MALDI-TOFMS an ideal method for fast and accurate diagnostic sequencing. The p53 tumour suppressor gene was chosen as a model system to demonstrate the feasibility of diagnostic sequencing by mass spectrometry due to the high frequency of mutations which occurred in exons 5–8.

Using an optimized single tube process for target amplification, solid phase sequencing and sample conditioning for MALDI-MS, the total sequence of exons 5–8 has been

0142-0453/98 $12 00 © 1998 Taylor & Francis Ltd obtained through primer walking. Gel-based artifacts caused by unresolved secondary structure in GC-rich sequence regions are irrelevant in MALDI-MS. MALDI-TOFMS with delayed extraction provides high mass-resolution and accuracy, which enable immediate based identification and differentiation of false stops by calculating the mass difference of neighbouring peaks. Twin peaks generated after a polymorphism/mutation site provide unambiguous identification of heterozygosity. These unique advantages coupled with its intrinsic high speed for mass separation make MALDI-TOFMS a powerful technique for clinical diagnostics as well as genomic research. Initial MALDI-TOFMS results of p53 sequencing samples loaded by micro-volume delivering systems, e.g. piezoelectric pipette and pintool, were encouraging, holding promise for future automation and cost reduction.

### Identification and sequencing of an MHC-associated peptide related to *trypanosoma cruzi* infection by tandem mass spectrometry

*Adam H. Brockman,[1,3] Ron Orlando,[1] and Rick L. Tarleton[2], [1] The Complex Carbohydrate Research Center, [2] and The Department of Cellular Biology, University of Georgia, Athens, GA 30602, USA; [3] Current address: Covance Laboratories, P.O. Box 7545, Madison, WI 53707–7545, USA*

Major histocompatibility complex (MHC) molecules serve to present self and non-self peptides to immune system T cells in order to effect an immune response against intracellular pathogens. By identifying and sequencing peptides from pathogen-infected tissues, it is hoped that protein sequences can be identified which are capable of stimulating an immune response against infectious organisms and cancers before they become established in the host, or to limit their proliferation following establishment.

Cytotoxic T lymphocyte (CTL) assay is often utilized to identify peptide fractions from infected tissues which contain target epitopes. However, the assay can require high amounts of peptide due to the diversity of the infection-related peptides in the extracted population. In this work, we have sought to identify active peptides by the use of alternative methods. More specifically, we scanned databases and tabulated peptide sequences and molecular weights from *Trypanosoma cruzi* protein sequences that matched the known class I MHC-binding motif for our animal model ($K^b$ mice). We used this tabulated data to scan extracts for mass-to-charge (m/z) values of interest using mass spectrometry. Peptides with m/z ratios matching those of peptides from the database were then sequenced using tandem mass spectrometry, usually with the help of on-line microcapillary reversed-phase high performance liquid chromatography (HPLC). The success of acquiring this mass spectrometric data with subfemtomole sensitivity required the use of settings tailored for MHC-associated peptides. By this approach, we confirmed the processing of parent proteins by the MHC class I pathway.

### Closed-loop selectivity control for pressure tunable tandem GC columns using electronic pressure control

*Heather Smith and Richard Sacks, Department of Chemistry, University of Michigan, Ann Arbor, MI 48109, USA*

The use of tunable GC columns can result in dramatic reductions in analysis time. By adjusting the pressure at the junction point between two GC columns having different selectivity, the selectivity of the tandem ensemble of the columns can be tuned with the limits of the individual columns. If the individual columns differ considerably in selectivity, then relatively small changes in tuning pressure can cause significant changes in elution patterns. Electronic pressure control recently has been used to obtain very precise control of elution patterns. As columns age, the elution patterns can change and the tuning pressure has to be readjusted in order to re-establish system performance. In some applications, this can be very inconvenient. In addition, where columns need to be replaced, re-tuning is required. By the use of electronic pressure control, closed-loop tuning control can be achieved, and portions of the initial optimization and tuning operations can be automated.

A vector model for separations using tunable column ensembles can be used to determine the sensitivity of the resolution of a critical component pair to changes in tuning pressure. The model will be described and used to determine the degree of pressure control needed in order to maintain adequate separation of a specified component pair. The system hardware and closed-loop tuning algorithms will be described. Long-term stability and elution patterns for test mixtures will be considered. Examples will be presented for the high-speed separation of mixtures or organochloride compounds.

### Multivariate optical computation for predictive spectroscopy

*M. L. Myrick, Matthew P. Nelson and Jeffrey F. Aust, Department of Chemistry and Biochemistry, University of South Carolina, Columbia, SC 29208, USA; J. A. Dobrowolski and P. G. Verly, Institute of Microstructural Sciences, National Research Council of Canada, Ottawa, Ontario, Canada, K1A 0R6*

A novel approach to predicting chemical and physical properties based on principal component analysis (PCA) is proposed and evaluated using a data set from earlier work. In our approach, a regression vector produced by PCA is designed into the structure of a set of paired optical filters. Light passing through the paired filters produces an analogue detector signal (direct product) directly proportional to the chemical/physical property (% cure) for which the regression vector was designed.

This simple optical computational method for predictive spectroscopy is evaluated in several ways, using the example data for numeric simulation. First, we evaluate the sensitivity of the method to various types of spectroscopic errors commonly encountered, and find the method to have the same susceptibilities toward error as standard methods. Second, we use propagation of errors to determine the effects of detector noise on the predictive power of the method, finding the optical computation approach to have a large multiplex advantage over conventional methods. Third, we use two different design approaches to the construction of the paired filter set for the example measurement to evaluate manufacturability, finding that adequate methods exist to design appropriate optical devices. Fourth, we numerically simulate the predictive errors introduced by design errors in the paired filters, finding that predictive errors are not increased over conventional methods. Fifth, we consider how the performance of the methods is affected by light intensities that are not linearly related to chemical composition (as in transmission spectroscopy), and find that the method is only marginally affected.

### On-line determination of mercury in the chlor-alkali industry

*W. T. Corns, P. B. Stockwell and N. Brahma, P S Analytical, Arthur House, Crayfields Industrial Estate, Main Road, Orpington, Kent BR5 3HP, UK; H. Evans and L. Ebdon, University of Plymouth, Drake Circus, Plymouth, Devon PL4 8AA, UK*

The electrolysis of saturated brine using a mercury flowing cathode is still the most efficient method of production for caustic soda. Despite elaborate and efficient mercury removal systems, it is inevitable that small quantities of mercury are transferred to the products of this process. Consequently, there is a need to accurately measure the levels of mercury both in the products themselves and the corresponding waste effluents being discharged from the plant.

Fully automated on-line instrumentation has been devised for a number of samples and effluent streams. The design challenges of these situations, particularly in relation to the control of the chemistry and materials of construction, were discussed. The solutions evolved for a number of these challenging issues were presented.

### New development in enhanced automated pressure control system

*Jing Zhong, Cornell University Research Park, Building #4, 83 Brown Road, Ithaca NY 14850, USA*

For most high pressure control applications, the Automated Pressure Control System (APCS) can generate very accurate results. The APCS comprises a piston-driven pressure generator, a motorized pressure regulator, and motorized valves. The limitation for the APCS is its relatively small capacity and slow refilling speed, which make the conventional system inconvenient and inefficient for certain larger volume applications.

To improve the system performance, the newly developed Enhanced Automated Pressure Control System (EAPCS) combines a digital piston pressure generator and an air-driven hydraulic pump to generate large volume, high pressure output in reduced time. The EAPCS is a microprocessor-controlled, fully automated pressure control system that uses customized Windows-based software.

Testing features of the EPACS include user-defined multiple target pressure steps, pressurization and depressurization rates, holding times and pressure cycling between pre-set maximum and minimum pressures. An improved PID control algorithm was applied to quickly compensate for run-time adjustments to the target pressure and run-time corrections for small leaks in the system. In addition, the EAPCS utilized customized data acquisition software for easy operation and convenient data storage

### Development and performance of an automated air analyser

*Raimund Roehl and Mike Doyle, Dionex Corporation, 1228 Titan Way, Sunnyvale, CA 94088, USA*

In this contribution, the authors reported on recent advances in the development of an automated air sampling and analysis system. Acidic or basic contaminants in air, e.g. HF, HCl, $SO_2$, $NO_2$, $Na^+$, $NH_3$ and volatile amines are absorbed in an aqueous trapping solution using an impinger with diffuser. The reaction products with water are preconcentrated on an ion exchange concentrator column, and then separated and quantitated by ion chromatography with suppressed conductivity detection.

The air analyser can be located up to 30 m away from the sampling site, and perform analyses continuously and in an unattended fashion for several days. The typical sampling frequency is two to three samples per hour. Acidic or basic components in an air or gas sample can be determined at part-per-billion and even part-per-trillion levels. The analytical sensitivity is adjusted by selecting the air sampling time (typically in the range 10–40 min) and the volume of preconcentrated absorber solution (1, 5 or 10 ml) appropriate for a given application.

Recent data collected in semiconductor production facilities and clean rooms for hard disk manufacturing have shown that the system is capable of detecting acidic and basic contaminants in the air of class 100 and class 10 clean rooms. Its excellent sensitivity is derived from the combination of ion chromatography and preconcentration.

Since analyses are performed continuously and results are available immediately after the completion of each analysis, the system can track temporal variations of contaminants close to real time. This capability is particularly useful in industrial clean room monitoring where the occurrence of even low levels of molecular containments can have a profound effect on production yields.

### Real time chemical imaging instrument based on fibre optical imaging compression

*Jiaying Ma, Brian McClain and Dor Ben-Amotz, Department of Chemistry, Purdue University, West Lafayette, IN 47907, USA*

A new fibre bundle-image-compression (FIC) technique is used to measure spectral images without the need to scan either the detection wavelength or spatial position of the sample (or light source). This allows the collection of spectral images up to 1000 times faster than scanning methods, and thus opens the way to the development of a real-time chemical imaging sensor with wide ranging applications in robotic vision, materials diagnostics and medical imaging. Applications of this method to Raman imaging were demonstrated in imaging salt ($KNO_3$ and $K_2SO_4$) and sugar (fructose and glucose) mixed micro-crystal samples. The signal-to-noise advantage of the FIC method relative to tunable filter and other scanning methods was analysed. The FIC technique may be applied to various optical scattering, reflectance and absorption spectroscopies, including Raman scattering and fluorescence, which require active illumination, and passively illuminated UV-VIS-NIR absorption reflectance. The key feature of the FIC design is a fibre-optic bundle used to compress 2D images onto a 1D fibre stack, which serves as the entrance slit of an imaging spectrograph. Thus, complete optical spectra are simultaneously collected from every point within a sample in a single scan of a charge-coupled-device (CCD) detector. In other words, the $x, y$ and $\lambda$ dimensions of the spectral image are all measured simultaneously, rather than sequentially using either a scanning tunable filter or stripe illumination.

### A new diode array based multiparameter continuous on-line monitoring system

*Bhupendra Patel, Bran + Luebbe, 1025 Busch Pkwy, Buffalo Grove, IL 60089, USA; Arne Hansen, Bran + Luebbe GmbH, Werkstrasse 4, D22844 Nordestedt, Germany*

The DiaMon represents a new generation of on-line systems which allows you to analyse multiple parameters on one single instrument and within one analytical run. It contains a diode array spectrometer to measure the complete UV/VIS spectra in one step. The DiaMon combines traditional colorimetric methods with new and innovative uv/vis-chemometrics. A direct application would be in the controlling of a wastewater treatment plant: the DiaMon provides the ability to analyse parameters, e.g. phosphate, ammonium, nitrate and nitrite together with cumulative parameters, e.g. COD, DOC and others; all simultaneously. Other applications are in the process control of plating baths or the closed loop control of a portable water plant.

The DiaMon provides all the benefits known from on-line analysers, e.g. fully automatic operation, self-checking functions, high stability and precision. The integrated computer with a full graphic display and keypad allows for complete control of all functions including remote control via modem.

### Real-time monitoring of single-protein exchange at the liquid/silica interface

*Xiao-Hong Nancy Xu and Edward S. Yeung, Ames Lab-USDOE and Department of Chemistry, Iowa State University, Ames IA 50011, USA*

Real-time continuous monitoring of single-molecule retention and partition at the liquid/silica interface was achieved using a combination of laser-induced fluorescence spectroscopy, evanescent wave excitation and an intensified charge-coupled device camera (ICCD). Fluorescence images were recorded from the same set of isolated molecules excited either within the evanescent field at the quartz–liquid interface or as a thin layer of solution defined by micrometre-sized wires, giving diffraction-limited resolution of inter-connected attolitre volume elements. Electronic synchronization with the initiation of a reaction permits the observation of each molecule as it reacts, providing a window for us to determine how experimental conditions or environments can affect reaction rates and equilibria at the single-molecule level. Direct comparisons of single-molecule reactions with macroscopic measurements are now possible.

Real-time continuous monitoring of single-molecule retention and partition at the liquid/silica interface was achieved with a spatial resolution of 0.3 µm and temporal resolution of 0.2 ms. Exchange dynamics of single-molecules (e.g. individual rhodamine molecule and protein conjugated with rhodamine) at the interface were measured in real-time. Each single-molecule displayed individual retention and partition characteristics at the interface. The retention and partition of single-molecules or single-ions at the interface were a function of pH and ionic strength. For the first time, chromatographic interactions at the single-molecule level were observed in real-time. The prospective applications of these studies and the detailed experimental configurations were discussed in the presentation.

### Rapid optical characterization of individual micro diameter particles

*Kenneth D. Hughes and Kevin D. Crawford, School of Chemistry and Biochemistry, Georgia Institute of Technology, Atlanta, GA 30332-0400, USA*

Initial results indicate that it is possible to generate a rapid and simple optical method for the characterization of individual micron diameter particles that contain polystyrene. Monitoring visible fluorescence intensity from individual particles allows the determination of polymer presence in multicomponent systems, and in some situations weight percentage and relative particle porosity.

The development of synthesis schemes for the production of micron and sub-micron diameter particles with several different polymeric components is a focus of significant research effort. Particles in this size range exhibit many interesting characteristics and have been utilized to modify the performance of synthetic materials, e.g. epoxies, as drug delivery agents and as chemical probes. One of the major difficulties in developing synthetic protocols for the production of multi-component polymer particles has been the analysis of individual particles comprising the bulk product. Currently, electron microscopies are the methods of choice for these investigations.

Investigation of polymer particles by electron microscopy requires time-consuming sample preparation and analysis. The rapid fluorescence method that will be discussed is based upon visible excitation of individual polymer particles immobilized in a visible optical trap or isolated on a microscope slide. Photochemical and solvent-mediated reactions are used to generate fluorescent species in the polystyrene backbone. Spectral characteristics include a broad emission from 500 to 800 nm

(488 nm excitation) which is observable by eye in most instances. Quantification of this emission provides determination of individual particle characteristics and the sub-populations present in a bulk sample.

This optical technique is providing quantitative information concerning polymer weight percentage, the spatial distribution of polymer components, particle porosity and other physical characteristics. This information provides quick feedback for synthetic method development and facilitates a better understanding of material applications.

### Chemical profiling the thermal degradation products of polymeric and composite materials

*Doug B. King, LECO Corporation, 600 Kenrick Dr., Suite C18, Houston, TX 77060, USA*

Recent developments in polymer chemistry have created numerous new materials encompassing a wide range of properties. Some are formulated for strength while others are developed for use in high temperature environments. The processes involved in the fabrication of microelectronics require these advanced polymers to withstand higher temperature environments.

A technique has been developed using an external thermal inlet system interfaced to a gas chromatograph-mass spectrometer (GC-MS) to allow monitoring of thermal degradation products from materials being heated over a wide temperature range (50–700°C) in a flow of inert helium.

Samples in this study were analysed using the instrumentation configured in two separate and distinct techniques. The first technique involved rapid sample heating to 700°C, cryogenic focusing and GC-MS separation of the degradation products. Major ions from the resulting chromatographic peaks were observed and recorded for later use. The second technique involved the same instrumentation; however, the sample was reheated at a constant rate of 30°C/min and no cryogenic focusing was involved. Instead, the out-gassed or degraded sample products were transferred to a hot (320°C) GC injection port and column, and then directly into the MS. In this configuration there is no GC separation but rather a continuous evolution of all degradation products over the time required to heat the sample.

The resulting total ion 'thermograms' contain all the mass ions scanned during data acquisition. By selecting various major ions determined from the original GC-MS run, a series of specific ion thermograms was displayed and compared. An overlay of the sample temperature heating rate onto the retention time of the ion thermograms allowed monitoring of the temperature range at which the production of various degradation products begins.

### Capillary electrophoresis: a valuable component for the lab-on-a-chip

*J. Michael Ramsey, Oak Ridge National Laboratory, P.O. Box 2008, Oak Ridge, TN 37831-6142, USA*

Microfabricated devices that perform chemical analysis procedures have primarily focused on chemical separation methods. The first such device was a gas chromatograph fabricated on a silicon wafer, reported over a decade ago. This seminal work did not produce significant interest from the chemical measurements community due to low performance characteristics. More recently there has been renewed interest in microfabricated chemical instruments, but with a focus on electrophoresis devices and analysis of fluids in general. In moving to the liquid state and employing electrokinetic transport phenomena, microfabricated chemical analysis devices have delivered performance features equivalent to or exceeding that of conventional laboratory instruments. Furthermore, these new devices show promise for integration of multiple laboratory functions that accomplish a complete assay, i.e. placing the features of an entire laboratory on a chip. Ultimately one would like to have a collection of micro-laboratory components (the analogues of resistors, transistors, etc.) that could be designed into new devices to solve specific measurement problems. Realization of these micro-laboratory components could allow a paradigm shift for chemical and biochemical synthesis and analysis similar to that provided to electronics by the transistor and integrated circuit. Some of the components we have developed for the laboratory on a chip will be described. The importance of chemical separations components will be stressed.

### Watching individual chemical reactions in femtolitre volumes

*R. Mark Wightman, Cory Curtis and Erin McDonald, Department of Chemistry, University of North Carolina at Chapel Hill, Chapel Hill, NC 27599-3290, USA*

Electrogenerated chemiluminescence (ECL), the production of light from intermediates generated during electrolysis, occurs when the energy liberated by chemical reactions is sufficient to generate a species in an electronically excited form. This phenomenon has long been of interest since it provides a simple route for photon production. Excited state formation in such reactions is a consequence of the Marcus prediction that formation of the ground state is sufficiently exothermic that it lies in an inverted kinetic region. The efficient nature of ECL, coupled with the simplicity of photonic detection, has led to its use in trace analytical methods, with subpicomolar detection limits reported in some applications.

In recent work, we have investigated the generation of ECL with microelectrodes. A significant advantage to this approach is that high-speed potential switching can be used. In this way the diffusion layer in which the reagents producing light are found is confined to a region within a few nanometers from the electrode surface. When used with ECL systems that arise from the reaction of electrogenerated radicals, the short time scale of the experiment ensures that they react before dilution and reaction with trace impurities occur. In dilute solutions and with electrodes of micro dimensions, the number of molecules involved in the light generation approaches a countable number. This provides the unique opportunity to observe chemical reactions under stochastic conditions.

With higher concentrations, the light is visible by eye, and the location of nanometer tips can be observed as a consequence of the ECL reaction

### Using neural networks and statistical methods to determine the geographic origin of potatoes

*Brian W. Smith, Department of Pure and Applied Mathematics, Washington State University, Pullman, WA 99164, USA*

Protecting a grower's/producer's reputation and consumer confidence to pay a premium for their produce/food is meaningful to the food industry and regional economies. Misidentifying a commodities growing region deceives the consumer. Overpayment by consumers for a commodity hurts not only the consumer, but eventually affects growers and producers. Produce-marketing groups each claim the quality of their branded fruits and vegetables is enhanced by superior growing conditions in their region. Protecting a quality reputation is essential to the industry.

*Buyer-Beware* in the age of labelling (and nutritional labelling) requirements in our current legislative environment is no longer a valid caution unscrupulous retailers can employ. Consumers should have available the same information already available to importers, shippers and retailers. Consumers are beginning to demand to know where their food is coming from and have the opportunity to buy geographically defined produce.

The use of neural network and statistical pattern recognition techniques to determine the geographic origin of potatoes based on trace element concentrations will be examined. The benefits of multi-dimensional analysis was discussed. Examples using 18 variables and nearly 1000 samples were used to illustrate neural network modelling techniques. Application challenges of neural networks, e.g. 'over-fitting' were discussed. Finally, a comparison of neural network modelling and some statistical approaches was presented.

### Raman spectroscopy at the production line—some benefits and pitfalls

*Neil Everall and Bert King, ICI Technology, Wilton Research Centre, PO Box 90, Wilton, Middlesbrough, Cleveland TS90 8JE, UK*

Recent advances in holographic optical technology have meant that highly efficient, compact and robust fibre-coupled Raman systems, suitable for at or on-line analysis, are now readily available to industrialists. In this presentation, we discussed *in situ* and on-line Raman measurements which we made on both solid and liquid systems to allow process/reaction monitoring and control. It was shown how Process Raman Analysis provides an automated replacement for other at-line analytical techniques, which often require intensive manual effort in sampling and subsequent analysis. The use of Chemometrics for analysing Process Raman spectra was discussed, including the (automated) data pretreatments necessary to allow effective analysis of spectra with a significant fluorescence background. Without this pretreatment, techniques, e.g. Principal Components Analysis or Partial Least Squares were not effective. We showed how changes in polymer morphology and composition can be followed as a function of process conditions.

We also discussed the influence of laser wavelength, and levels of recycled material in polymer streams, on background fluorescence, using laser excitational 532 and 785 nm to illustrate these points. Moving solid polymers can have an intense background fluorescence under 532 nm excitation, due to the failure of fluorescence quenching/bleaching. Changing to 785 nm excitation prevented this fluorescence for virgin polymer, but inclusion of recycled (and thus slightly degraded material) can regenerate the fluorescence signal.

Finally, we also considered some of the more difficult sampling conditions for on-line Raman analysis. Systems, e.g. dilute turbid emulsions place significant demands on the optical collection design. It is difficult for the laser to penetrate turbid solutions sufficiently to obtain good Raman data, and often one obtains strong spectra for the window material (even silica is a significant Raman scatter if the laser cannot reach the sample!). Unless the laser is focused precisely at the window/liquid interface, a poor spectrum results. The benefits of alternative window materials were considered.

### Non-invasive Raman spectroscopy of pharamaceuticals in USP vials: calibration, reproducibility, and library searching

*Richard L. McCreery, Angela Horn, Jack Spencer and Everett Jefferson, Department of Chemistry, The Ohio State University, 100 West 18th Avenue, Columbus, OH 43210, USA*

The revolution in Raman instrumentation has resulted in compact, integrated spectrometers of practical utility in chemical analysis. With these hardware and software developments in hand, a major advantage in the area of sampling has emerged. Raman spectra can often be acquired rapidly and non-invasively, through bottles, blister packs and even translucent packaging.

The current discussion centres on rapid qualitative identification of organic solids. A commercial dispersive Raman spectrometer operating at 785 nm with a CCD detector was used to acquire spectra of USP reference materials inside amber USP vials. The laser and collection beams were directed through the bottom of the vials, resulting in a 60% loss of signal. Raman shift was calibrated with a 4-acetamidophenol standard, and spectral response was corrected with a luminescent standard. After these corrections, the Raman spectra obtained inside the USP vial and on open powders differed by less than 5%. A spectral library of 309 reference materials was constructed, with spectral acquisition times ranging from 1–60 s. Of these, 8% had significant fluorescent background but observable Raman features, while 3% showed only fluorescence. A blind test of 26 unknowns revealed the accuracy of the library search to be 88–96%, depending on search algorithm, and 100% if operator discretion was permitted. The tolerance of the library search to degraded signal to noise ratio, resolution and Raman shift accuracy were tested, and the search was very robust. The results demonstrate that Raman spectroscopy provides a rapid, non-invasive technique for compound identification.

### FT-IR spectroscopy as a rapid QC tool for the food industry

*Michael C. Garry, Nicolet Instrument Corporation, 5225 Verona Road, Madison, WI 53711, USA*

Fourier transform infrared (FT-IR) spectroscopy provides information about molecular structures of chemical species. This fact has allowed FT-IR spectrometers to serve as a valuable identification tool in many industrial laboratories. With improved computer and spectrometer technology, advancements in sampling accessories, better quantitative software and focused application development efforts, FT-IR has expanded in use for quality control and process monitoring. The ability of mid-infrared spectral analysis to obtain detailed functional group characterization allows this technique to replace or supplement many wet chemical titration methods in manufacturing facility laboratories. Speed of analysis and the ability to analyse samples directly without significant sample preparation are the key features that make this technique appealing.

This presentation focused on the use of FT-IR spectrometers in the food industry for the rapid analysis of key chemical parameters related to fats and oils and sweeteners. Topics that were discussed include the types of chemical parameters assessed, and how sampling and software interfaces are used to make the system simple to use in the industrial quality control laboratory.

### Simultaneous near-infrared determination of total acidity and dissolved solids in concentrate orange juice

*Paulo Roberto Saliba, Renato Guchardi and Celio Pasquini, Institute de Quimica, Universidade Estadual de Campinas, C.P. 6154, CEP 13083-970, Campinas, SP, Brazil*

Concentrate orange juice produced in Brazil is exported widely through the world. The quality control of the juice is mainly based on the measurement of two parameters: the total acidity and the dissolved solids (BRIX). This work attempted to investigate the use of transflectance measurements of concentrate juice in the NIR region (1400–2500 nm) to access, simultaneously, both parameters.

Tranflectance measurements were obtained in a 3-mm-long optical cell. The NIR spectrophotometer was assembled in the laboratory and employed an Acoustic-Optical Tunable Filter (AOTF—Brimrose) as monochromator. Light is guided to and from the measurement cell through two liquid-filled optical cables (Lumatec) (OG). Transflectance were calculated taking the empty cell transmittance spectrum as reference. A transflectance spectrum can be obtained in 20 s and the total measurement time is about 1 min. The juice is used without water remotion. The results show that the SEPs (18 samples) for total acidity and BRIX, obtained by a PLS model (30 samples, five principal components) are $0.25\,g\,l^{-1}$ and 0.38%, respectively. These results show that the proposed method can be employed to replace the conventional methodologies employed for concentrate juice quality control.

### Non-invasive determination of peanut maturity using near-infrared reflectance spectroscopy

*Casey C. Grimm, Olga Richard, Bryan Vinyard and Elaine Champagne, USDA-ARS-SRRC, 1100 Robert E. Lee Blvd, New Orleans, LA 70124, USA; Timothy H. Saunders, USDA-ARS-MQHR, Box 7624, NC State University, Raleigh, NC 27695-7624, USA*

A near-infrared reflectance spectrum was obtained on each of 100 intact peanuts. The exocarp of the peanut hull was then removed and the maturity-related colour of the mesocarp determined using a Hunter Colorimeter. The colour of the mesocarp of the peanut hull has been related by Williams and Drexler to the maturity of the peanut. Using half of the samples ($N = 50$), regression analysis was performed to produce a mathematical model relating the NIR spectra to the Hunter $L^*$, $a^*$ and $b^*$ values. Using the model, Hunter $L^*$ and $b^*$ values were then predicted for the remaining 50 peanut spectra. Hunter $a^*$ has been shown to have a non-linear relationship with maturity. A correlation was observed for the NIR spectra and the Hunter $L^*$ and $b^*$ values. Comparison of the predicted results against the measured Hunter $L^*$ and $b^*$ values gave a standard error of validation of 2.4 and 2.3, respectively. The range of the Hunter $L$ value was from 0 to 100, resulting in a relative error of less than 5% ($\pm 2 \times$ SEV). The prediction of a Hunter $b^*$ value due to its shorter angle (5–24) resulted in a relative error of *ca.* 18%. Possible techniques for automating the analysis were also discussed.

### Precise measurement of binding to proteins by automated headspace gas chromatography

*Ravindra P. J. Ranatunga and Peter W. Carr, Department of Chemistry, University of Minnesota, Minneapolis, MN 55455, USA*

The measurement of the binding of small non-electrolytes whose spectra do not shift upon binding is a difficult problem. Headspace gas chromatography has therefore been developed as a tool for the measurement of binding of volatile non-electrolytes to proteins. The approach involves the gas chromatographic analysis of the headspace in equilibrium with a solution containing alkylbenzenes at infinitely dilute concentrations, addition of aliquots of dissolved protein under computer control, allowing for re-equilibration between the headspace and solution, followed by the analysis of the headspace for alkylbenzenes. The partition coefficient for distribution of the solutes betwen the buffer solution and the protein is obtained from the slope of an appropriate plot of normalized peak area against the amount of protein added. It was found that the free energy of binding of the alkylbenezenes to bovine albumin, as well as the $\Delta G^{\circ}$ per methylene group, were smaller compared to a transfer process, e.g. from water to hexadecane. The binding of alkylbenzenes with proteins, e.g. bovine serum albumin and chicken egg albumin (ovalbumin) were found to be vastly dissimilar, indicating that the binding properties of these proteins are significantly different from each other.

### Application of modified gas chromatograph to analyse space experiment combustion gases on space shuttle mission (STS-94)

*William K. Coho, NASA Lewis Research Center, Cleveland, OH 44135, USA; David M. Vanzandt, Aerospace Design and Fabrication, Brookpark, OH 44142, USA*

A space experiment designed to study the behaviour of combustion without the gravitational effects of buoyancy was launched aboard the Space Shuttle Columbia on 1 July 1997. The space experiment, designated as CM-1, was one of several space experiments manifested on the MSL-1 (Microgravity Sciences Laboratory No. 1) Mission. The launch, designated STS-94, had the Spacelab Module as the payload, in which the MSL-1 experiments were conducted by the Shuttle crew-members.

CM-1 (Combustion Module for MSL-1) was designed to accommodate two different combustion experiments during MSL-1. One experiment, the investigation of the Structure of Flame Balls in Low Lewis-number (SOFBALL), required Gas Chromatography Analysis to:

(1) verify the composition of the known, premixed gases prior to combustion;
(2) determine the combustion by-products resulting from the combustion process in microgravity.

A commercial, off-the-shelf, Dual-Channel Micro Gas Chromatograph was procurred and modified to:

(1) accept Spacelab electrical power;
(2) accommodate two different carrier gasses, each flowing through its own independent column module;
(3) withstand the launch environment of the Space Shuttle;
(4) interface with the CM-1 Fluids Supply Package and the CM-1 Combustion Chamber;
(5) meet the Spacelab Flight Requirements for EMI and Offgassing.

Argon was used as the carrier gas through a Mol Sieve 5A Column and helium was used as the carrier gas through a PoraPlot Q Column. The resulting GC analytical data were downlinked to Project Scientists at Houston.

### Further development in digital signal processing techniques for multiple modulation FTIR measurements

*David L. Drapcho, Raul Curbelo, Richard A. Crocombe, Lizhong Zhang and David Johnston, Bio-Rad Laboratories, Digilab Division, 237 Putnam Avenue, Cambridge, MA 02139, USA*

In previous papers, we demonstrated the use of digital signal processing (DSP) software algorithms for demodulating the phase modulation component in step-scan FTIR photoacoustic measurements, and the simultaneous demodulation of both the phase modulation and the sample modulation components in dynamic polymer stretching measurements. These software techniques eliminate the requirement for one or more lock-in amplifiers for demodulation of the component signals, thereby eliminating the inherent cost and experimental complexity of the lock-in amplifiers. In addition, the DSP software techniques provide increased sensitivity by allowing the full use of the dynamic range of the FTIR spectrometer. In contributing developments in this area, we will discuss the development of DSP algorithms to triple modulation experiments utilizing a photoelastic modulator (PEM). Preliminary results are shown below. Experiments in this application include vibrational circular dichroism, vibration linear dichroism and dynamic infrared linear dichroism measurements.

### Spectral library searching: mid-infrared versus near-infrared spectra of neat food ingredient powders

*James B. Reeves III and Charles M. Zapf, USDA, Agricultural Research Service, Nutrient Conservation and Metabolism Lab., Bldg 200, Rm 124, Beltsville Agricultural Research Center East, Beltsville, MD 20705 and McCormick & Company Center, 202 Wight Avenue, Hunt Valley, MD 21031-1066, USA*

The objective was to determine the potential of using mid- or near-infrared diffuse reflectance spectra to construct food ingredient spectral libraries for product identification and checking. Samples (106) consisting of buttermilk, dehydrated onion, cheese and milk-egg powders, wheat flours and two powdered seasonings were scanned (neat powders, diffuse reflectance) at 4 and 16 $cm^{-1}$ resolution in the mid-infrared on a Digi-Lab FTS-60 and Perkin Elmer Model 2000, and in the near-infrared, FTS-60 (4 and 16 $cm^{-1}$ resolution) and NIR-Systems model 6500 scanning monochromator (10 nm bandwidth). A custom-made sample transport device was used on the FTS-60, a rotating sample cup on the NIRSystems 6500 and a stationary cell for the Perkin Elmer 2000. Every third sample of each group was used as a test sample and searched against a library containing the remaining samples. Results showed that only full spectrum-based searches using Euclidian distance of correlation (with or without a first derivative) were useful. All unknowns were correctly matched using near-infrared spectra generated on either the scanning monochromator or the FTS-60 (4 $cm^{-1}$ resolution), or using any of the mid-infrared spectra. Results demonstrated that near- or mid-infrared spectral libraries of powdered food ingredients can be used for product identification and checking. Finally, spectral interpretation should be considerably easier using mid-infrared spectra.

### Automated multidimensional gas chromatography

*Medha J. Tomlinson, Charles R. Foster, Tania A. Sasaki and Charles L. Wilkins, University of California-Riverside, Department of Chemistry, Riverside, CA 92521-0403, USA*

Gas chromatography (GC) systems with coupled columns in a multidimensional (MD) mode have shown potential utility for the analysis of complex mixtures. Enhanced selectivity is obtained through selective transfer of the sample and by use of different stationary phases. Analyte transfer from a first-stage column to a subsequent analytical column is accomplished with the use of mechanical valves or by utilizing a valveless pressure switching device based on the design originally developed by Deans. Such heartcutting techniques have the advantage of analysing the component of interest by reducing the interference from matrix components. In such systems, a single cryogenic trap is most often used between two serially coupled columns. For complex samples, this approach can be time consuming if many heartcuts from the same chromatogram need to be analysed.

Use of multiple parallel traps can significantly reduce the time required for complete sample analysis because more than one heartcut can be trapped for each primary injection. Since many valves are required to direct the effluent to the appropriate trap, detector and column, parallel trap experiments are operator-intensive. To overcome this problem, we have developed an automated MDGC system using combined infrared and mass spectral detection. Selection of the heartcut, trapping and reinjection is accomplished with computer-controlled valves using a program written in Microsoft Visual Basic. Although valveless switching is generally considered as being the best method for sample transfer in MDGC, mechanical valve-based systems have been shown to function as well as their valveless counterparts. The present design is based on the success of a semi-automated system, which allowed the automated selection of a heartcut and manual reinjection of the trapped components. Since only one trap was used, analysis times were longer than desirable. The present system includes seven traps, thereby allowing many sections of a chromatogram to be trapped and a choice of two columns, to allow enhanced selectivity of the trapped components. Results obtained with the automated system for both standard and unknown mixtures were presented.

### New developments in automated analytical and preparative HPLC/MS for the rapid characterization and purification of compound libraries

*Lu Zeng, Xiaoli Wang and Daniel B. Kassel, CombiChem, 9050 Camio Santa Fe, San Diego, CA 92121, USA*

A fully automated analytical and preparative HPLC/MS method has been developed and applied to the characterization and purification of combinatorial libraries. Our automated LC/MS system incorporates fast, RP-HPLC (5 min analyses) and ESI-MS. PrepLCMS is a method we developed, and is the first system that permits automated and rapid purification of multi-milligram quantities of compound libraries replying on mass spectrometric ion signals to 'trigger' fraction collection. This one-sample one-fraction format means that 'batches' of components can be purified without the need for excessively large fraction collector beds.

Recent advances include the use of reverse and normal phase HPLC/APCI-MS, and the development of an automated multiple column or parallel column analytical/preparative HPLC/MS system. By using two columns, two UV detectors and dual electrospray ion source, the parallel HPLC/MS system provides higher throughput analysis and purification of compound libraries.

### New instrumentation for high throughput SPE coupled on-line to HPLC and MS

*J. Bert Ooms, Otto Halmingh and Gerard S. J. Haak, Spark Holland B.V, P. de Keyserstraat 8, P.O. Box 388, 7800 AJ, Emmen, The Netherlands*

Solid Phase Extraction coupled on-line to HPLC or (LC)-MS ('hyphenated SPE' is our preferred terminology) is increasingly used for analysis with integrated sample clean-up, especially when large numbers of biological samples are involved. Hyphenated SPE provides full automation of the entire assay (plasma or urine samples are analysed without any manual treatment), and better precision and sensitivity because the extract is quantitatively transferred to the HPLC or MS with minimum dilution. Continuing efforts, especially in the pharmaceutical industry, to reduce the assay cycle time have led to very short LC run times and mass spectrometry as the preferred mode of detection. The high-resolution power of the MS-MS systems has greatly reduced the need for efficiency in the LC separation, thus allowing LC-MS analysis times of only a few minutes. Since hyphenated SPE takes place during analysis of the previous sample, it is important that SPE can be performed with the same speed as the analysis to prevent the SPE procedure becoming the rate-limiting step for the assay.

We presented new approaches for fully automated high throughput hyphenated SPE based on the PROSPEKT technology. Innovations include: parallel processing with two PROSPEKT units for one MS, extended cartridge capacity (220 per PROSPEKT) and system plumbing modifications to speed up SPE-LC/MS assays. In combination with small cartridge dimensions and small sorbent particles, high SPE efficiency is obtained in a very short time. Examples of short assay cycle times were shown, including the application of direct SPE-MS, where the SPE cartridge performs both clean-up and separation prior to MS analysis. A special system for rapid automated SPE method development was also presented and discussed.

### Determination of hexachloro-hydrocarbons in ground water by automated SPE extraction

*Edward M. Conroy and Robert S. Johnson, Horizon Technology, 8 Commerce Dr., Atkinson, NH 03811, USA*

The determination of hexachloro-hydrocarbons from ground water samples is well documented. At the present time, EPA methods 625 and 8270 are predominantly used for the determination of the hexachloro compounds. These methods currently specify the use of liquid–liquid extraction (LLE), or continuous liquid extraction to extract these compounds. However, LLE and the subsequent concentration steps have well-known problems which can adversely effect recoveries.

In comparison, solid phase extraction (SPE), in a disk or membrane format, offers many benefits over conventional liquid extraction techniques. Significant benefits are faster sample throughput, higher recoveries and lower sample costs. Due to these benefits, more methods are being written to include SPE as an alternative extraction technique. Plus, as more selective packing phases become available for SPE disks, the ability to extract more volatile and water-soluble compounds increases.

By combining the benefits of SPE disks with a fully automated extraction system, it is now possible to achieve higher and more consistent recoveries for the hexachloro compounds. We reviewed the automation steps and techniques used to process ground water samples and achieve these higher recovery values. Also, several critical variables that can adversely affect these recoveries were discussed.

### Automated analysis of chlorinated phenols with SPE disks

*Edward M. Conroy and Robert S. Johnson, Horizon Technology, 8 Commerce Dr., Atkinson, NH 03811, USA*

Environmental requirements concerning the concentration levels of chlorinated phenols in ground water are of great health importance. As with most environmental samples, some type of extraction and concentration technique must be used prior to analysis by the analytical instrument. Liquid–liquid extraction (LLE) using a separatory funnel is still typically the extraction method used for aqueous samples when performing SW-846 methods. However, LLE and the associated concentration steps have well-known problems which can adversely affect recoveries. These problems are further complicated when trying to extract polar phenolic compounds, i.e. low and inconsistent recoveries.

With the introduction of Solid Phase Extraction (SPE) disks, many of the associated problems with LLE (i.e. emulsions) were addressed, but recoveries for the more polar compounds were still low and inconsistent.

Recent introductions of new disk phases designed for water-soluble compounds have increased recoveries, but due to problems associated with manual SPE operations, consistent recoveries are still difficult to achieve.

In order to achieve higher and more consistent recovery levels of chlorinated phenols when using SPE disks, it is important to minimize operator intervention in the extraction process. We reviewed the automated steps and techniques used to achieve higher recovery values for chlorinated phenols from ground water. Also, several critical variables, which must be understood and resolved before attempting to analyse aqueous samples for these compounds, were discussed.

### The development of a proposed EPA method for the determination of arsenic and selenium in potable water based on atomic fluorescence spectrometry

*W. T. Corns and P. B. Stockwell, P S Analytical, Arthur House, Crayfields Industrial Estate, Main Road, Orpington, Kent BR5 3HP, UK; N. Bloom and D. Wallschlaeger, Frontier GeoScience, Environmental Research Corporation, 414 Pontius North, Seattle, WA 98109, USA*

Hydride generation-atomic absorption spectrometry (HG-AAS) is a well-established technique for the determination of arsenic and selenium. Natural levels of arsenic and selenium found in potable waters are often less than the current method detection limits obtainable by EPA methods. As a direct consequence, there is therefore a need for a more sensitive, selective and automated method for these elements.

Hydride generation, coupled to atomic fluorescence spectrometry was extensively evaluated and shown to offer superior performances, particularly in terms of sensitivity. Method detection limits of 10 and 20 $ng^{-1}$ were established for total arsenic and selenium, respectively. The detection system utilizes a boosted discharge hollow cathode lamp as an excitation source, a chemically generated hydrogen diffusion flame for atomization of the gaseous hydrides and a short bandpass filter to achieve wavelength isolation. Results were presented for a range of potable waters, and possibilities for other applications were presented.

### Characterization of microdialysis cells for continuous sampling

*Christine M. Zook and William R. Lacourse, Department of Chemistry, University of Maryland, Baltimore County, 1000 Hilltop Circle, Baltimore, MD 21250, USA*

Microdialysis provides continuous samples. This sampling technique utilizes semi-permeable membranes to separate a flowing perfusion medium from the matrix of interest. Small molecules able to cross the membrane are collected by the perfusion medium and are collected for analysis. In this way, large molecules are excluded from the sample, making additional sample clean-up unnecessary. Clean-up may also include charge exclusion. The sampling is continuous, making this technique ideal for monitoring reaction or process kinetics.

Microdialysis cells which accept flat dialysis membranes have been designed in order to study the membranes used in this process as well as to control the sampling environment as to temperature and hydrodynamics. Carbohydrates are sampled and then analysed using High Performance Anion Exchange Chromatography-Pulsed Amperometric Detection (HPAEC-PAD). By using cells of this type, membranes of different materials can be exchanged and characterized. Membranes tested included those of cellulose ester and regenerated cellulose. Performance of these cells was examined with respect to recovery of analyte, response time and reproducibility of recovery. The monitoring of enzyme kinetics is an application which illustrates the utility of these cells.

### Monitoring electrically stimulated release of dopamine with voltammetry and microdialysis: insights into quantitation of *in vivo* dopamine levels

*Jennifer L. Peters and Adrian C. Michael, Department of Chemistry, University of Pittsburgh, Pittsburgh, PA 15260, USA*

Microdialysis is an analytical technique which is commonly used to measure dopamine in the brain. However, little agreement exists on how to quantitate the concentrations measured by microdialysis, resulting in a widely disparate estimates of basal extracellular dopamine concentration. A goal of this work is to examine the impact of the brain environment on *in vivo* measurements in order to gain a better understanding about what factors influence the concentration of dopamine measured by various devices. We have used fast scan cyclic voltammetry and microdialysis under identical experimental conditions, allowing direct comparison of the results. Carbon fibre microelectrodes were used to monitor the electrically stimulated release of dopamine in dialysate, brain tissue and brain tissue directly adjacent to microdialysis probes, both before and after uptake inhibition.

The key results of this study were:

- Spatial heterogeneities exist within the tissue on a micrometer scale.
- The tissue surrounding microdialysis probes is abnormal with respect to release of dopamine.

Based on our experimental results, we have developed a simple mathematical model which couples mass transfer and kinetics. Furthermore, the model does not assume a spatially homogeneous extracellular dopamine concentration, allowing the effect of heterogeneity to be examined. The model qualitatively agrees with our experimental results and can further be applied to steady state microdialysis results produced by other laboratories, allowing transient and steady state voltammetry and microdialysis experiments to be interpreted within a single theoretical framework. Our calculations strongly suggest that quantitative interpretation of *in vivo* dopamine concentrations requires detailed information about diffusion rates, uptake kinetics, the spatial distribution of release sites in the tissue under examination, and the extent to which probe induced damage impacts the tissue.

### Monitoring neuropeptide release from neuroendocrine cells of *Aplysia* using MALDI mass spectrometry

*Lingjun Li, Scott, A. Shippy, Rebecca W. Garden, Philip D. Floyd, Stanislav S. Rubakhin and Jonathan V. Sweedler, Department of Chemistry, Beckman Institute, University of Illinois, Urbana, IL 61801, USA*

The neuroendocrine bag cells of the marine mollusk *Aplysia californica* provide an attractive model system to investigate neuronal signalling and function. Matrix-assisted laser desorption ionization (MALDI) coupled with time-of-flight (TOF) mass spectrometry is a sensitive and powerful tool for rapid peptide profiling in biological cells. Although cellular MALDI analysis assays the peptides present in cells, the ability to follow neuropeptide release is desirable to understand their physiological roles. However, two challenges to following neuropeptide release from marine species using MALDI MS are the high salt extracellular matrix and small sample volumes. While matrix rinsing allows peptides to be measured from intact cells, measurement of peptides released is carried out in high salt (~ 500 mM NaCl) extracellular media. Due to the small sample volume and relatively low peptide concentration, many conventional desalting methods are problematic. We reported a new capillary-based sampling system, which allows direct analysis of cellular neuropeptide release in high salt matrices using MALDI-TOF MS. Experimental strategies and results were discussed. Briefly, the bag cell neurons are electrically or chemically stimulated, and releasates are collected via gravimetric flow at the end of the sampling capillary followed by addition of DHB matrix. A typical MALDI mass spectrum from bag cell releasate revealed that both identified and novel processing peptide products are released in a calcium-dependent manner upon stimulation, suggesting their potential role in signal transduction or cellular communication. Additionally, a modified capillary-based microdialysis sampling system was applied to follow peptide release from juvenile *Aplysia* bag cell neurons. This promises a better insight into developmental aspects of neuronal signalling.

### Tracking gene expression and enzymatic processing in single neurons with MALDI mass spectrometry

*Rebecca W. Garden, Tatiana P. Moroz, Lingjun Li and Jonathan V. Sweedler, Department of Chemistry, University of Illinois at Urbana-Champaign, Urbana, IL 61801, USA*

Proteolytic cleavage of neuropeptide precursors is a well-regulated process, where multiple bioactive products, encoded by a single gene, often have diverse functions. Many cells throughout the nervous system of the invertebrate model *Aplysia californica* are well characterized, in terms of neural circuitry and DNA, yet the physiological significance of co-existing transmitters is poorly understood. In order to gain further insight into the roles played by identified genes, we developed matrix assisted laser desorption/ionization (MALDI) with time-of-flight mass spectrometry (TOF MS) to survey the gene expression patterns and peptide processing in freshly isolated as well as cultured cells from *Aplysia* and other marine molluscs.

Single cells, clusters and connective tissues are isolated by microdissection in the presence of a MALDI matrix (e.g. aqueous 2,5-dihydroxybenzoic acid) and transferred onto a sample probe using tungsten needles. Alternatively, neuronal networks grown in culture on a custom MALDI probe insert can be spatially profiled by aiming the laser at different regions of the cell(s). High quality mass spectra are obtained with resolution and mass accuracy typical for the instrument employed. A semiquantitative method is developed for comparing cell profiles using spectral normalization.

A mass spectrum, combined with knowledge of expressed genes, is often enough information to identify peptides and determine new processing pathways. However, there are cases when either the prohormones are not known or the gene products have been modified in an unpredictable manner. Hence, it is desirable to obtain supplemental, sequence-revealing information. One viable approach is to use cellular MALDI in conjunction with specific proteolytic hydrolysis. As one example, acquiring mass spectra both before and after pyroglutmate aminopeptidase treatment enables the detection of *in vivo* N-terminal cyclization.

Our technique is inherently different from conventional methods, in that peptides present at significant concentrations are detected, not only those which are preselected or radioactively tagged. Moreover, we demonstrated the ease of directly screening single cells without biochemical separation of the cellular matrix. Sampling, data acquisition and strategies for identifying novel post-translational modifications and genetic mutations were presented, which reveal the utility of MALDI-TOF MS for cellular analysis.

### Preparative scale chiral separations by conventional gel electrophoresis

*Samuel R. Gratz, Richard M. C. Sutton and Apryll M. Stalcup, Department of Chemistry, University of Cincinnati, Cincinnati, OH 45221-0172, USA*

The area of chiral separations has seen tremendous progress in recent years with the development of new chiral stationary phases and the discovery of new chiral selectors. However, an overwhelming majority of the work in this area has targeted analytical scale separations. Consequently, the ability to perform preparative scale chiral separations has been left glaringly deficient.

The use of conventional gel electrophoresis for the chiral separations of mg quantities of pharmaceutical compounds was presented. Instrumentation, optimization and recovery strategies were discussed. Separations were accomplished using Bio-Rad's Mini Prep Cell, a continuous elution, vertical gel system, with agarose used as a mechanical support impregnated with a chiral selector. Separation conditions are first developed using capillary electrophoresis and then transferred to the preparative system. During a run, one end of the electrophoresis bed is continually washed with an elution buffer which is sent to a standard HPLC UV detector and then to a fraction collector. Fractions identified from the UV trace as containing analyte are then subjected to CE analysis to determine enantiomeric composition. We have obtained both enantiomers of chiral analytes with greater than 99% enantiomeric purity.

### Optimizing HPLC instrumentation and separations for the characterization of combinatorial chemistry libraries

*Michael E. Swartz, Waters Corporation, 34 Maple Street, Dept. TG, Milford, MA 01757, USA*

HPLC plays a vital role in the characterization of libraries obtained by the combinatorial chemistry process. However, in this role, HPLC faces many unique challenges; perhaps the most demanding of which is the need for high sample throughput, on both an analytical and preparative scale. Purity and identification are required, often from samples for widely different chromatographic properties. Suitable sample preparation and handling are also critical to success. The development and optimization of an HPLC separation must address these needs, often in a 'generic' manner in order to be applicable to a wide range of samples. Various components of HPLC, e.g. instrumentation, software and chemistry all play a role, and a holistic approach to optimizing the use of these components results in higher efficiency.

This presentation addressed the optimization of various parameters necessary to accomplish high sample throughput, including column and mobile phase chemistry, as well as instrumentation and software design and utilization. High throughput gradient methods on the order of minutes per run (injection to injection), were shown, and the use of advanced features of modern HPLC instrumentation necessary to significantly reduce analysis and characterization time were highlighted.

### Combinatorial library screening with and without affinity capillary electroporesis

*Yen-Ho Chu and Zhiguang Yu, Department of Chemistry, The Ohio State University, Columbus, OH 43210, USA*

Recent progress in combinatorial screening for lead compounds from both soluble libraries and libraries on solid supports will be discussed. In the case of soluble libraries, affinity capillary electrophoresis/mass spectrometry was developed as an integrated, solution-based approach to drug lead discovery. This technology incorporates into one step the two essential parts of a screening process: compound selection and structure identification. A great deal of information can be rapidly obtained in conjunction with efficient assay methods, the strategy of deconvolution can also be employed in the search for lead candidates from soluble libraries.

For the analysis of libraries on solid supports, it is suggested that combinatorial library may be useful in the search of diagnostic agents and cross-reactive epitopes.

### Protein crystal growth by automated dynamic control of temperature

*Alireza Arabshahi and W. William Wilson, Center for Macromolecular Crystallography (CMC), University of Alabama at Birmingham, Birmingham, AL 35294, and Department of Chemistry, Mississippi State University, MS 39762, USA*

The quest for obtaining large, high quality protein crystals for understanding structure–function relationships by X-ray crystallography is the driving force to optimize crystals growth methodology. Recently, these crystallographic studies have become of great interest to biomedical, pharmaceutical and chemical industries, as a promising tool in drug design, protein engineering and other biological applications. Recent advances in microtechnology were used to develop a device that uses laser light scattering as a non-perturbing probe for detecting both the aggregation and post-nucleation stages of protein crystal growth. Dynamic control of various stages of crystal growth was achieved by imposing temperature changes. This report presented various temperature profiles and ramp rates that were used to grow crystals in a way to influence the size, number and quality of protein crystals.

### Real-time molecular scale investigations of plasma protein adsorption

*Truong Ta, Michael Sykes and Mark T. McDermott, Department of Chemistry, University of Alberta, Edmonton, Alberta, Canada T6G 2G2*

The adsorption of proteins to biosensors continues to be a large hurdle in sensor development. Sensor versatility and robustness can be achieved if protein adsorption is minimized. Thus, our studies in this area are focused on understanding the influence of surface chemistry on the formation of protein films and on the orientation of adsorbed biomolecules.

Our approach involves the use of scanning force microscopy (SFM) to monitor the growth of fibrinogen films in real time on highly oriented pyrolytic graphite (HOPG) and mica surfaces. We find that film formation on these two well-defined surfaces proceeds through different mechanisms. On HOPG surfaces, fibrinogen layers evolve through the growth of a network structure. This observation implies the involvement of strong protein–protein interactions. In contrast, the growth of fibrinogen films on mica does not include significant lateral intermolecular interactions. These different growth mechanisms are manifested in the final film morphology and structure.

We also presented our preliminary results describing the influence of specific functional groups on the formation of protein films. In these studies, infrared reflection adsorption spectroscopy and SFM were combined to give a macroscopic and microscopic description of protein adsorption at gold supported self-assembled monolayers.

### Multigram preparative separation of enantiomers in support of drug discovery activities

*Lester A. Dolak, Thomas M. Castle and Eric P. Seest, Structural, Analytical and Medicinal Chemistry, Pharmacia and Upjohn, 301 Henrietta Street, Kalamazoo MI 49001, USA*

The discovery of new drugs is an expensive process. Few synthesized molecules reach drug candidate status, much less the market place. Teams of scientists from many disciplines pool their talents in an effort to shorten the time required to select a single, best lead compound from those discovered to be active in high volume screening programmes. The sources of the test compounds can be natural products libraries acquired by in-house synthesis over many years, purchase and combinatorial synthesis. Collections of tens of thousands of compounds can be routinely tested in a panel of assays deemed relevant to therapeutic reality on a weekly basis. Compounds that give a favourable response in any of these assays are further tested and may become the basis of analogue programmes that intend to optimize the biological response of the lead compound.

When the lead compound is asymmetric, one must determine which isomer to develop. It is currently very difficult to develop a mixture of isomers, e.g. as racemate, to a marketed drug. On the contrary, several drugs initially sold as racemates are now marketed as the single enantiomer responsible for the therapeutic activity. In the early stages of drug discovery, one does not know which stereoisomer will be superior.

Several methods are available for the determination. First, one could apply stereoselective synthetic methodology and separately prepare each enantiomer in suitable purity for evaluation. Secondly, one could prepare diastereomeric derivatives or salts of the racemate, and separate the enantiomers on the basis of physical chemical properties, e.g. by crystallization. Thirdly, one could employ enzymatic resolution. Finally, one can separate the enantiomers by chromatography on a chiral stationary phase (CSP).

We discussed our experiences with the application of automated HPLC techniques and CSPs to racemates of interest in the drug discovery activities of Pharmacia and Upjohn. We described our approach to resolution problems, our instrumentation and related issues, e.g. safety and costs.

### Automation, instrumentation and approaches to the rapid screening of pharmaceutical enantioseparations

*Gerald J. Terfloth, SmithKline Beecham Pharmaceutical, 709 Swedeland Road, King of Prussia, PA 19406, USA*

The pharmaceutical industry is faced with a number of challenges which require new approaches in the way it discovers, identifies and manufactures new drug substances. Some of the challenges in a chemical development environment are directly connected to the increased molecular complexity. Reducing the time required to obtain the first gram to kilogram quantity of a chiral drug candidate has a strong impact on the development process.

This presentation discussed a possible strategy for integrating software tools and standardized hardware for screening, developing and scaling-up separations from an analytical to pilot scale. The application of packed-column supercritical fluid chromatography, capillary electrochromatography, thin layer chromatography and HPLC was evaluated on the background of different problems. The issue of throughput in batch, continuous and parallel HPLC was addressed.

### Computer simulation of the influence of kinetics in capillary electrophoresis

*Daniel L. Hopkins and Victoria L McGuffin, Department of Chemistry, Michigan State University, East Lansing, Michigan 48824-1322, USA*

A Monte Carlo computer simulation has been developed to study the influence of kinetics in capillary electrophoresis. The program includes algorithms for the transport processes of diffusion, electroosmotic flow and electrophoretic migration that provide a comprehensive description of the electrophoretic process. In addition, algorithms for first-order and pseudo first-order kinetic interconversion of species can be invoked when the solute molecules exist as more than one species. These algorithms are appropriate for acid–base equilibria in a buffered medium, as well as metal ion complexation equilbria when the ligand concentration is relatively high. In this case, each molecule is randomly assigned a lifetime from a distribution derived for the specific reaction pathway. If more than one reaction pathway is available, random lifetimes are selected for each pathway and the shortest lifetime is then selected for the molecule. When the incremented simulation time is equal to the assigned lifetime for the molecule, it is converted from the original to the new species according to the appropriate reaction pathway. The properties of the new species (electrophoretic mobility, diffusion coefficient, etc.) are applied during its assigned lifetime. The algorithms for the transport and kinetic processes have been fully validated by comparison with classical transport and kinetic theories.

The computer simulation has been used to study the electrophoretic behaviour of solutes with two, three or four interconvertible species having a wide range of kinetic rate constants and equilibrium constants. The results indicated that slow interconversion of species leads to increased broadening of the solute zone profile, as suggested in previous experimental studies, but also may lead to persistent asymmetry. These effects may be more pronounced when discontinuities or gradients in buffer pH or ligand concentration lead to deviations from steady-state conditions. The computer simulation can be used to provide guidance in the selection of experimental conditions to minimize these detrimental effects.

### On-line capillary electrophoresis detection using microbeads

*Yan He and Lei Geng, Department of Chemistry, University of Iowa, Iowa City, IA, 52242, USA*

We explore a new detection method for capillary electrophoresis based on packed silica beads. Silica beads with diameters of ~ 5μm are packed into a capillary. Two frits are formed to hold the silica beads in an optical detection zone. The microbeads can be either unmodified or coated with molecular probes. The coupling of a capillary electrophoresis instrument made-in-house and lasers with a commercial spectrometer allows detection by laser-induced steady state fluorescence, time-resolved fluorescence and Raman spectroscopy. The detection method provides information on the interaction between the analyte and the surface of the microbeads. It thus has the potential of chemical speciation, sensitivity enhancement and probing molecular mechanisms in capillary electrochromatography.

### Optimizing capillary electrophoresis-mass spectrometry: separation and sensitivity

*Thomas E. Wheat and Kim A. Lille, Thermo BioAnalysis, 8 East Forge Parkway, Franklin, MA 02038, USA*

Capillary electrophoresis coupled to mass spectrometry provides a powerful analytical tool that enhances the value of either technique practiced separately. The mechanical and electrical aspects of the interface have been well documented, and commercial adapter kits are available. While numerous useful applications have been demonstrated, the chemical aspects of this hyphenated technique are less well developed. The mass spectrometer imposes constraints on the use of the range of CE buffers typically used for separations monitored with absorbance detectors. Most CE-MS separations have been performed in dilute acetic acid or ammonium acetate with an acidified aqueous–organic electrospray sheath liquid. Further, the sensitivity of the technique is often inadequate for authentic samples because of the low volume and mass capacity of the separation capillary. In this report, we described buffer optimization strategies to give improved CE separations. The effects of different buffer ions and co-ions were demonstrated, as was the effect of ionic strength. Particular attention was given to the impact of such manipulations on sensitivity. Options for adjusting the composition of the sheath liquid in concert with the separation buffer were described. The effect of changing electrospray operating parameters to obtain a better signal-to-noise ratio for a given separation buffer was also explored. In particular, positive and negative ionization modes were compared for appropriate analytes. The principles developed in this series of experiments were demonstrated with a variety of samples, including proteins, peptides, small molecules of pharmaceutical significance, and a variety of industrial chemicals.

### Development of pattern recognition methods for the identification of an environmentally important analyte (TCE) in diverse backgrounds using fluorescence data from an optical sensor array

*S. R. Johnson, H. L. Engelhardt, J. M. Sutter and P. C. Jurs, Chemistry Department, Penn State University, University Park, PA 16802, USA; T. A. Dickinson and D. R. Walt, Chemistry Department, Tufts University, Medford, MA 02155, USA; J. White and J. S. Kauer, Department of Neuroscience, Tufts University School of Medicine, Boston, MA 02111, USA*

The design of chemical sensing systems has traditionally involved the development of a single sensor with high selectivity for a single analyte. This approach becomes increasingly cumbersome as the number of monitored analytes grows. The mammalian olfactory system utilizes a collection of semi-selective receptor cells which generate a response pattern which can then be related to the odorant of interest. This results in high sensitivity and broad-band detection, which has generated significant interest in the development of arrays of cross-reactive sensors analogous to the mammalian nose. In this report, we present the development and application of pattern recognition methods which exploit the temporal and multiple wavelength information obtained from an optical sensor array for the classification of trichloroethylene (TCE) from several different relevant chemical backgrounds. In addition, a separate model was developed to identify the background component of each mixture.

The data used for this study were collected using an array of 19 fibre-optic sensors each coated with a different polymer formulation containing a solvatochromic dye. The response of each sensor consisted of the change in fluorescence intensity of the dye at two monitored wavelengths in response to an analyte presentation. The site of analytes consisted of TCE as a pure compound and in combination with various relevant chemical background species at several concentration ratios. The fluorescence signal for each observation was encoded with numerical descriptors designed to focus on important features that were useful for analysis. A fuzzy ARTMAP computational neural network model was trained to indicate the presence or absence of TCE for each observation. The descriptors forming each model were selected using the genetic algorithm; the fitness function was evaluated using the fuzzy ARTMAP computational neural network. Correct predictions were made for more than 94% of the observations in an external prediction set held aside for validation of the model.

### The development of a quantitative structure–property relationship for the prediction of dielectric constants using neural networks

*Robert C. Schweitzer and Jeffrey B. Morris, Army Research Lab, AMRL-WM-PC, Aberdeen Proving Ground, MD 21005-5066, USA*

The dielectric constant is an important fundamental molecular property that can be useful in the prediction of the behaviour of substances on a macroscopic scale. One of the cases in which knowledge of the dielectric constant is useful is in the selection of appropriate modifiers for supercritical fluid extraction. Since the dielectric constants of potential modifiers are not always readily available in the literature, a method is needed that allows the prediction of dielectric constants.

Quantitative structure–property relationships (QSPR) are calibration models in which the independent variables are molecular descriptors calculated from the molecular structure. These models are built using a calibration set of structures for which the molecular descriptors can be calculated and for which the dielectric constants are known. Given such a model, the dielectric constants for structures not present in the calibration set can be calculated. There are many classical multivariate calibration techniques available which can be used to build calibration models. These include multivariate linear regression, partial least-squares regression and principal components regression. The technique used in this research, however, was the neural network. The advantage of neural networks is that they develop a non-linear relationship among the input variables without requiring a predefined relationships specified by the user. The training algorithm used in this work to find the optimal weight values was the Broyden–Fletcher–Goldfarb–Shanno quasi-Newton algorithm.

A data set of 800 compounds for which dielectric constant data were available is compiled. Three-dimensional structures are calculated using MM2. Dipole moments, polarizabilities and partial atomic charges are calculated using Gaussian 94. Simple molecular descriptors, e.g. counts of the number of atoms of a particular element, and the presence or absence of hydrogen bonding capability are calculated. Two other descriptors calculated are molecular connectivity descriptors and charged partial surface area descriptors. In all, a total of 65 descriptors was calculated for a subset of the 800 compounds. Many QSPRs are developed for various subsets of descriptors in a search for the set of descriptors that yields the lowest mean error for the calibration set and test set. The results of these screening experiments were given along with more in-depth analyses of the best QSPRs.

### Neural networks in the analysis of water soluble sulphonylurea herbicides using LC/DAD/MS

*Joseph M. Pompano and Sarah C. Rutan, Department of Chemistry, Virginia Commonwealth University, Richmond, VA 23284-2006, USA*

Because of environmental concerns, more efficient pesticide residue screening methods are now needed to protect the public from unwanted side effects of pesticide use. One class of pesticides, the sulphonylureas, is known for its high herbicidal activity, which results in a very low application rate.

Methods that can measure several pesticides at a time offer great advantages in time and effort, e.g. the coupling of high performance liquid chromatography (HPLC) with mass spectrometry (MS). This combination offers the excellent separation ability of HPLC with the selectivity of MS. In addition, photodiode array detection (DAD) can be used as a selective and sensitive detector in HPLC. New chemometric data analysis techniques are currently being developed to handle these large amounts of data. One subfield, artificial neural networks (ANNs), has seen explosive growth in the past decade.

ANNs are useful tools when a problem cannot be solved by other computational techniques due to noisy data or lack of a specific model. ANNs are also very good at handling non-linear data or outliers in data.

Nine sulphonylurea herbicides were the target analytes in a study of the ability of an ANN to simplify the sample preparation needed for analysis using an LC/DAD/MS instrument. A feed-forward, back propagation ANN was developed in the Matlab programming environment. This network modulated itself while running, by using singular value decomposition of the hidden layer output, in order to determine the optimum number of nodes in the hidden layer. This should help in preventing overfitting. This ANN was used in the analysis of the LC/DAD/MS data of the sulphonylurea herbicides.

### Chemometrics as a competitive strategy for R&D

*Suzanne Schönkopf, Camo ASA, Box 1405 Vika, N-0115 Oslo, Norway*

Chemometrics is today recognized as a means for multivariate calibration of multichannel instruments, e.g. spectroscopy. In addition, many companies worldwide utilize chemometrics with success within a broad range of other applications in research and development. They thus understand their processes better, develop new products more efficiently, and make more rational and better measurement programmes for quality management and process control.

Despite the benefits of the methods, there are still relatively few users, compared to their enormous potential, because the chemometrics education is still young and sparse, and corporate management is not aware of the possibilities. To illustrate the large potential of chemometrics we will present a range of different applications (quality analysis, process optimization, materials characterization, rational testing, simulation of tests, QSAR, method development, etc.) within many different industries (paper and pulp, pharmaceutical, petroleum, food, chemical).

Implementation of chemometrics on a broader basis in the R&D is possible. Major companies that successfully have taken this approach and gained millions of dollars define the following criteria as critical for success; tools, knowledge, organization, acknowledgement by the management and profiling. A strategy to achieve this was presented.

### Direct measurement of testosterone and epitestosterone conjugates in urine using HPLC/MS/MS

*David J. Borts and Larry D. Bowers, Athletic Drug Testing and Toxicology Laboratory, Department of Pathology and Laboratory Medicine, Indiana University Medical Center, 635 Barnhill Drive MS A-128, Indianapolis, IN 46202, USA*

The ratio of urinary testosterone (T) to epitestosterone (E) conjugates is used as an indicator of steroid doping in athletes. Presently, the T/E ratio is determined by GC/MS. The GC/MS analysis requires enzymatic or acidic hydrolysis of the steroid conjugates, followed by derivatization. These steps are time consuming and can produce erroneous results because of the ineffectiveness of the enzymatic hydrolysis on steroid sulphate conjugates and the interconversion of steroid species caused by acid hydrolysis.

An LC/MS/MS method has been developed by quantitate urinary T and E conjugates directly, bypassing the hydrolysis and derivatization steps. In this method, urine samples are subjected to a solid phase extraction, the extract is injected for microbore liquid chromatography separation, and the steroid conjugates are detected directly by electrospray ionization tandem mass spectrometry. Because of the complexity of the urine extract, there was extensive variation in inter- and intra-sample ion intensity suppression. In addition, the structural similarity of the many endogenous steroids led to ambiguous qualitative identification by mass spectrometry. These problems were overcome through the use of a deuterated internal standard for each of the analytes of interest. The LC/MS/MS method is fast, simple and eliminates the quantitative recovery problems associated with the conventional GC/MS method. The method is sufficiently sensitive and specific for the routine analysis of T and E conjugates in urine, and demonstrates the importance of including T and E sulphates in the calculation of T/E ratios.

### Microwave digestion of petroleum crudes and petrochemical products with subsequent analysis via ICP and a CID-ECHELLE spectrometer

*J. David Hwang, David Leong, Toni Z. Miao, NGA Malekzadeh and Andy C. Bell, Chevron Research and Technology Company, 100 Chevron Way, Richmond, CA 94802, USA*

For many years, analytical laboratories in the petroleum industry have prepared samples for subsequent analysis by conventional ashing and air-refluxing methods. These traditional methods are always time consuming, labour intensive, tedious and cumbersome. Additionally, contamination is very likely to occur from the acids (especially sulphuric acid) and/or vessels used during sample preparation. Moreover, sulphur determination may not be achieved because sulphuric acid is usually used in these methods.

We have recently developed innovative and rapid analytical methods that use a high pressure microwave system. The high operating pressure attainable in this closed microwave digestion system ensures total decomposition of complex petroleum crudes and petrochemical products, with no residual organic content. Sample preparation time has been shortened from days (or, in some cases, even more than a week) to minutes. These preparation methods are useful for the determination of sulphur in crudes and petrochemical products, e.g. polymer samples, because sulphuric acid is not necessary in the microwave digestion process. The microwave digestion system also reduces contamination from more traditional digestion vessels and airborne particulates in the lab environment. Additionally, because the microwave digestion approach requires less sample, smaller amounts of reagents are needed and less chemical waste is generated.

ICP-AES analyses are prone to errors due to changes in power level, nebulization rate, plasma temperature and sample matrix. This is especially true for ICP-AES applications in the petroleum industry where extremely complicated sample matrices are common. As a result, accurate analyses of petroleum crudes and related materials often require bracketing with matrix matched standards, the use of internal standards and a flexible ICP-AES system. An ICP spectrometer using a solid state detector, e.g. the Charge Injection Device (CID), fits these requirements well and is a powerful tool for problem solving. Examples in lubricant oils and additives as well as in catalysts were presented.

The CID-based ICP spectrometer provides both a sequential scanning monochromator (to pick and choose any needed wavelength) and a high throughput polychromator(to perform simultaneous multi-elemental analyses). This flexibility is in addition to improvements in absolute performance capabilities of the equipment.

### The measurement of mercury vapours flow from Earth crust and earthquakes prediction

*Yury I. Stakheev[1], Yury G. Tatsy[1], Yury A. Bakaldin[2], Tazbulat Ibrayev[1], Igor V. Bezsudnov[2] and Vladimir A. Sevryukov[2], [1] Vernadsky Institute of Geochemistry and Analytical Chemistry, 19 Kosygin Street, 117334 Moscow, Russia; [2]Scientific and Production Enterprise 'Nauka-Service', 11 (Bld 1) First Volkonsky Lane, 103473 Moscow, Russia*

The main source of mercury in the atmosphere is the upgoing stream of mercury vapours from Earth crust. The literature data confirm that mechanical destruction of solids caused gas realization not only from pores and inclusions, but as a result of the appearance of new fresh planes, along geological faults. The variations of mercury flow have been used as precursors for earthquake prediction.

Registrations of weak mercury flow require a high sensitive instrumentation and elimination of meteorological effects. Analytical system—plastic sampler placed underground, AF mercury analyser and bispiral gold collector for mercury preconcentration; closed gas system with flow rate 0.4 l/min; syringe calibration. Measurements were taken every hour in the unattended around-the-clock mode. Detection limit 10 pg of Hg; $S_r$ for 250 pg $< 0.05$ ($n = 30$). Mercury flow has been registered for more than three years; total number of measurements = 25 000. The computer processing of obtained information shows a lognormal distribution of results with mean value for mercury flow 0.22 ng/m$^2$h, and variations of about three orders of magnitude. Special experiments showed that mercury flow moves in an upright direction with a velocity of 2.6 cm/h.

Autocorrelation and FT analysis detect harmonic components in variations of mercury flow. The periods of these components (11.8; 23.4 h and 13.6 day) correspond to tide oscillations of the Earth crust as a result of Earth–Moon–Sun interaction. The real earthquake precursor for a magnitude of 3 and distance from epicentre of 15–20 km demonstrated a five-fold rise of mercury flow 29 h before and 90-fold rise 19 h before earthquake. The duration of precursors was usually 6–10 h. Fifty-one precursors were detected over a period of mercury flow registration. This method was used for prediction of time and intensity of earthquakes.

### Artificial 'noses' and their use in the beverage industry

*Carolyn M. Merkel and Roma Vazirani, McNeil Speciality Products, 501 George Street, New Brunswick, NJ 08903-2400, USA*

Artificial 'noses' can be used for a number of purposes in the beverage industry. For example, noses can assist in determining whether a raw material is acceptable or what is the source of off-odour or off-flavour in a questionable product. While the nose does not eliminate the use of humans in the determination of whether a product is good or bad, once the target quality has been determined, the nose can be used to repetitively screen samples in a rapid and cost-effective manner.

Another potential use for noses is assisting in the rapid development of optimal products. Once sufficient consumer acceptability information is obtained on a product type, test products can be screened for likely acceptability. Prescreening reduces the need for costly and lengthy consumer research tests, speeding up the development process. In this paper, the results of using an artificial nose to assist in the development of an optimal carbonated soft drink product were presented.

### Complementarity of QCM and MOS sensor technologies in coffee analysis

*Q. Lucas, J. Poling and V. Benincasa, Alpha MOS: 20, avenue Didier Daurat, 31400 Toulouse, France*

The quality control of coffee can be carried out at all stages of processing: from the raw material to the finished products. The control of the roasting process can lead to better quality and reduce wastage. It is very important to control the raw material green coffee, the finished product grounded or not grounded after the roasted process. Therefore, a quick and reliable method for testing the origin of the coffee, the roasted level and the finished product is necessary for the quality control of such raw materials.

The control of the purity of coffee at different levels previously defined are investigated using the Electronic Nose FOX5000.

This paper dealt with the optimization of the discrimination level using the different sensor technologies. These include metal oxide sensors, conducting polymer and quartz crystal microbalance.

This investigation showed that the quartz crystal microbalance and metal oxide sensors are complementary to define and describe the coffee.

### Potential use of the electronic nose in the tobacco industry: investigation of flavour enhancers and tobacco origin and quality

*O. Dutheil, Q. Lucas, J. Poling and V. Benincasa, Le St Exupery -20, Avenue Didier Daurat, 31 400 Toulouse, France*

The tobacco industry is very complex. It mixes chemistry, taste, flavour and marketing. This industry deals with various raw materials (tobacco), but also with many essential co-products (paper, filter, aroma enhancers, stabilizers, etc.). Furthermore, the extension of new legislation requires the improvement of Q/C procedure on all the raw materials and intermediates used in cigarette manufacturing. We investigated two applications with an hybrid Alpha MOS system using multi sensor arrays:

Glycerin analysis, and tobacco quality and grading

(1) A large set of glycerin classified as good and bad, from different suppliers has been analysed. Optimization of the analysis parameters has been performed in order to discriminate the different products and to train the system for new sample prediction.

(2) In addition we have analysed tobacco from different origins using a new optimization method and calibration protocol in order to assess successfully a prediction model with classical statistical method.

Good discrimination and stability were obtained in order to allow a successful identification of blind tests for the acceptable products.

In conclusion, it has been shown that the electronic nose for the qualification of tobaccos and additives was a fast and reliable technique. This phase has shown good performance which allows the start of the prediction of a model.

### Application of the electronic nose for quality control of edible oils

*Tze Tsung Tan, François Loubet and Sandrine Bazzo, Alpha MOS S.A., 20, Avenue Didier Daurat, 31400 Toulouse, France; Jacqueline D. Hewitt-Jones, Unilever Research Laboratorium Vlaardingen, Olivier van Noortlaan 120, 3133 at Vlaardingen, The Netherlands*

Different qualities of edible oils were successfully evaluated using a Fox3000 Electronic Nose (Fox3000 EN). Good discrimination and stability were obtained over a six week period for the successful identification of acceptable quality oil in 'blind tests'. Inter-batch training sets of the oil were used to ensure that a representative batch variation was introduced into the model using a custom identification method, and also to ensure that the discrimination level of the acceptable, marginal and unacceptable oils was sufficient when different batches were considered. Both Principal Component Analysis (PCA) and Discrimination Function Analysis (DFA) were used for data processing and identification. Good correlation between GC data, sensory panel and the Fox3000 EN were found. It is clear that for a successful application of Fox3000 EN, good instrument stability is necessary to ensure good and stable discrimination over time. In addition, good sample preparation techniques, including a low odour environment, were also necessary to obtain good repeatability of measurements. Finally, the principle of calibration was demonstrated by showing data for the edible oil with and without correction for drift by reference to the pure chemicals.

### Electronic nose evaluation of haircare polymers

*Brenda Hendrickson, International Speciality Products, 1361 Alps Road, Wayne, NJ 07470, USA*

Electronic nose technology is not meant to replace either GC or the human nose for odour evaluations, but to bridge the gap between objective and subjective evaluations, i.e. analytical and sensory testing. These three methods (headspace GC, electronic nose and human nose) when used together are able to define and identify more clearly customer and consumer needs for quality and consistency in personal care raw materials.

The electronic nose was able to detect resistance changes in the sensors due to the adsorption of vapour components of the polymers. We discussed the method of development for a polymer that contains ethanol and is used as a fixative in haircare products.

The odour profile of the polymer has several distinct characteristics that were analysed by a trained expert odour panel. We showed that the expert odour panel not only identified these characteristics, but determined their relative odour intensities and predicted acceptability in accordance with customer criteria.

We also showed that the differences observed by the electronic nose were found to be very complementary to the results obtained by headspace GC analysis, as well as expert odour panel sensory evaluation.

### Quality control of paper with an electronic nose

*Q. Lucas, J. F. Chauvet and V. Benincasa, Alpha MOS: 20, avenue Didier Daurat, 3140 Toulouse, France*

Packaging plays a very important role in the food industry and the quality of foods. No modification or transformation shall occur to the final product, e.g. contamination by residual solvents or additives, or degradation of the freshness. The manufacturer must package the finished products with the available materials as paper board. To meet these demands, the quality of material supplied is extremely important.

Since the final product quality can be influenced in such cases, a company must be able to control the packaging element through suitable quality tests. Today, sensory panels, GC-MS are often tied up to do this tedious and repetitive assessment. Electronic Nose can be used to lift this burden thanks to its different advantages (speed, no sample preparation, direct correlation with quality descriptors).

Analyses on paper boards, of various quality (different manufacturing process, concentration of residual solvents, coatings, inks) have been performed with an electronic nose, FOX4000 with 18 metal oxide sensors.

The data obtained have been processed with multivariate analysis (linear statistics) and neural networks (nonlinear) in order to predict the quality of unknown samples.

Analyses of coated paper of different qualities have been tested. The results of the FOX system are correlated with sensory panel data. Unknown samples are predicted after one month of training over a period of six months. The statistical data processing is able to visualize the territory of each quality. Moreover, the neural network trained recognized on-line the unknown samples with 98% of success.

Therefore, the electronic nose, FOX4000, has been successful in predicting the paper quality, and will be a very useful tool for accessing the quality of packaging materials rapidly with a single run using a very small amount of sample (a few grams).

### Automating sample preparation in capillary electrophoresis for enhanced separations and sensitivity

*Kim A. Lilley and Thomas E. Wheat, Thermo BioAnalysis, 8 East Forge Parkway, Franklin, MA 02038, USA*

High resolution separations and unique selectivity are attributes of capillary electrophoresis that have made the technique attractive for the analysis of a variety of complex samples. However, the analytes of interest are often found in complicated matrices and in relatively small quantities. Such samples can alter an established separation and can interfere with the detection of the analytes where unexpected co-migrations occur. It is also difficult to identify and quantitate the small analyte peaks in the presence of the more abundant matrix components. To address these problems, the established sample preparation techniques associated with alternative separation methods, e.g. HPLC or GC, can be adapted to CE. However, these techniques can be laborious and time consuming. On-line, automated sample preparation and concentration techniques, in particular, those that exploit unique electrophoretic mechanisms, would be a preferred approach.

The simplest form of sample preparation serves only to concentrate the sample. This can be achieved electrophoretically by stacking, field-amplified sample injection and transient isotachophoresis. The sample can be simplified by chemical fractionation, e.g. automated liquid–liquid extraction. Such processes can be enhanced by electroextraction that also serves to concentrate the analytes. Finally, the possibility to automate derivatization for CE methods allows the use of established derivitization techniques to improve the detectability, selectivity and separation. Each technique was demonstrated and optimized for several small molecules of pharmaceutical interest and of environmental concern.

### On-line sample preconcentration and NMR detection for capillary electrophoresis

*Dean L. Olson, Michael E. Lacey and Jonathan V. Sweedler, University of Illinois, Department of Chemistry, 600 S. Mathews, Urbana, IL 61801, USA*

Recent advances in NMR microcoils have led to their application as detectors for nanoliter samples common to capillary electrophoretic separations. A CE-MR microcoil made of copper wire is wrapped to form a solenoid around a capillary with an o.d. of 235 μm and an i.d. of 100 μm. CE is performed with on-line NMR data acquisition while the separated components reside in the microcoil observed volume (about 8 nl). As one example for 290 nl injection of a mixture of arginine and triethylamine (Arg and TEA), both at 50 mM (15 nmol of each injected), field-amplified sample stacking preconcentrates the components several-fold for improved NMR detectability. Under stopped-flow conditions, spectra are obtained in 1 min with NMR line widths of less than 2 Hz using a 300 MHz spectrometer. The limits of detection for CE-NMR (based on $S/N = 3$) are 57 ng (330 pmol; 41 nM) for Arg and 9 ng (88 pmol; 11 mM) for TEA. Other sample preconcentration methods using organic phases are discussed. In addition, the advantages of employing a magic angle NMR microcoil are presented. The separation ability of CE and the rich spectral information provided by NMR spectroscopy combine to yield a valuable analytical tool, especially in the study of mass-limited samples of biological origin or pharmaceutical interest.

### On-column sample preconcentration using pH-mediated stacking for capillary electrophoresis

*Sangyroul Park, Yeping Zhao and Craig E. Lunte, Department of Chemistry, University of Kansas, Lawrence, KS 66045, USA*

A significant limitation of capillary electrophoresis is the poor concentration detection limits, particularly with optical-based detection. Current on-column preconcentration techniques place significant restrictions on the sample matrix. For example, field-effect stacking requires that the sample conductivity be much lower than the electrophoresis background electrolyte (BGE) in order to produce a high electric field across the sample plug to achieve stacking. In this report, an on-column stacking technique, termed pH-mediated stacking, which uses manipulation of the BGE to achieve the low conductivity zone for sample stacking was described.

Using a BGE prepared from either a weak acid or weak base, a low conductivity zone can be temporarily created by titration of the BGE component through electrokinetic injection of a plug of strong acid or base. In acid stacking, the BGE is composed of a weak acid, e.g. acetic acid. During electrokinetic injection of the sample, acetate anions migrate into the sample zone displacing the sample anions. The subsequent electrokinetic injection of strong acid neutralizes the acetate creating a low conductivity zone across which sample cations stack. To preconcentrate sample anions, the electroosmotic flow is first reversed and the BGE is prepared from a weak acid, e.g. an ammonium salt. In this case, during electrokinetic injection of the sample, ammonium ions migrate into the sample zone displacing the cations. Subsequent electrokinetic injection of strong base titrates the ammonium cations creating the low conductivity zone across which the sample anions stack. For acid stacking, a sensitivity increase of 17-fold relative to normal electrokinetic injection was achieved without loss in separation efficiency for samples in Ringer's solution. Increases of over 50-fold in sensitivity relative to normal electrokinetic injection were achieved with base stacking.

The degree of stacking was limited by the maximum length of capillary that could be employed without producing unacceptably high electrophoretic currents. A double capillary approach was developed to overcome this limitation. In this approach, a tee is placed in the capillary. Stacking occurs in one arm of the tee after which the potentials are adjusted to force the electrophoretic separation to occur in the other arm of the tee. The use of the tee effectively allows the use of a much longer capillary without producing the higher currents, as only a short length of capillary is used at any time. Using the double capillary approach, a 300-fold increase in sensitivity was achieved without loss in separation efficiency.

### On-line proton-NMR microcoil detection for capillary isoelectric focusing

*Michael E. Lacey, Dean L. Olson and Jonathan V. Sweedler, Department of Chemistry, University of Illinois at Urbana-Champaign, Urbana, IL 61801, USA*

The high structural information content of NMR spectroscopy makes it desirable to use as a detector in separations. NMR has been coupled to CE, and methods which increase the concentration of analytes (e.g. preconcentration and focusing) improve NMR detectability. Isoelectric focusing in immobilized pH gradient gels offers reproducibility and stability over time, due primarily to the absence of free carrier ampholyte drift. However, such gel matrices for isoelectric focusing are more complex to prepare than comparable solutions of free carrier ampholytes.

A method to reproducibly generate immobilized pH gradient gels within a capillary (100 μm i.d.; 235 μm o.d.) is under investigation. The procedure (modified from conventional gel casting techniques calls for simultaneous injection of acidic and basic acrylamide solutions into a mixing chamber, from which the resulting solution flows into a capillary and polymerizes *in situ*. An NMR detection coil is fabricated by wrapping 50 μm copper wire directly around the separation capillary. The separated components may be transferred pneumatically to the observed region of the microcoil; alternatively, the capillary may be moved with a sleeve fitted with a microcoil.

Because of the large differences in NMR relaxation times between the analytes and the polyacrylamide gel matrix, NMR spin-echo pulse sequences can generate spectra with a reduced gel background signal while retaining the analyte signal. Capillary separations with NMR detection of biologically relevant samples are presented, and various electrophoretic separation modes compared.

### Continuous electrophoretic separations in narrow channels with pot-channel derivatization and laser-induced fluorescence detection

*Christine E. MacTaylor and Andrew G. Ewing, Department of Chemistry, Penn State University, 152 Davey Laboratory, University Park, PA 16802, USA*

Continuous channel electrophoretic separations with post-channel derivatization and laser-induced fluorescence detection are described. Previously, channel separations of dansylated amino acids and on-channel derivatized analytes have been demonstrated. Here, we describe the development of a new instrument design which allows for derivatization after the analytes have separated. A narrow bore electrophoresis capillary (25 μm i.d.) is coupled to a rectangular channel (15 cm × 5 cm × 25 μm). The capillary is used to continuously deposit material while moving across the entrance of the channel which produces a time axis. The separated analytes are derivatized with naphthalene dicarboxaldehyde (NDA), excited with an argon ion laser (488 nm), and fluorescence is then detected with a charge couple device.

This novel design is significant because post-channel derivatization is capable of eliminating multiple peaks formed when an analyte has more than one derivatization site. When analytes are derivatized before separation, their migration times vary depending on the number of sites that were actually derivatized.

This is an important development for studying cellular dynamics, since many biologically interesting analytes (i.e. proteins and peptides) have multiple derivatization sites.

### Continuous sampling and electrophoretic elution for monitoring dynamic chemical events

*Said M. Attiya and D. Jed Harrison, Department of Chemistry, University of Alberta, Edmonton, Alberta, Canada T6G 2G2*

Sample introduction into planar microfluidic devices is an area requiring further development. In this report, we demonstrate a sample interface integrated into the microfluidic chip structure, which in principle allows the chips to be used as monitoring stations for large reaction vessels. Within the chip a 1-mm-wide and 300-μm-deep rectangular channel was designed as a sample introduction channel (SIC). It connects with a 30-μm-wide and 9-μm-deep injection channel (IC). The drastic difference in the channel dimensions means the channels have radically different resistance to flow, allowing exchange of sample in one region without contaminating the rest of the chip. To interface with an external system, the chip was sandwiched between two plexiglas plates and connected with fittings to an external fluid line. When the sample was delivered by a peristaltic pump at 1 ml/min into the SIC, less than 4% of the length of the IC was filled by the sample. Thus, the rest of the channels were not contaminated.

To evaluate the device, an external beaker containing tris-boric acid, pH 9 served as a sample reservoir to interface to the microchip. Reservoir 1 was connected to the outlet of the peristaltic pump and the effluent of reservoir 2 was returned to the external beak. Fluorescein sample concentrations in the beaker was increased by stepwise addition of 10 μl aliquots of 1 mM fluorescein. The peristaltic pump delivered solution from the beaker through the SIC on-chip at 1 ml/min. The injection channel was then flushed for 30 s with the new sample by applying 3 kV between reservoir 3 and the beaker. Elution was then run on the sample.

### The on-line combination of direct PCR from whole blood and capillary array electrophoresis for DNA analysis

*Nanyan Zhang and Edward S. Yeung, Department of Chemistry, Ames Lab-DOE, Iowa State University, Ames, IA 50011, USA*

DNA is usually purified first from blood or other biological material before it is amplified by polymerase chain reaction (PCR). Here the PCR is performed by using whole blood as the template without pretreatment.

The blood samples from eight individuals were used for the genotyping of four loci: vWA, THO1, TPOX, CSF1PO. The four loci were amplified together inside a microreactor which is a 255 µm i.d., 360 µm o.d. fused silica capillary. The total reaction volume was 10.0 µl. The thermocycler was a hot air rapid thermocycler.

Capillary array electrophoresis has been used for high throughput DNA sequencing and genetic typing. Here, the PCR products of eight blood samples were on-line analysed by eight-capillary array electrophoresis system. The separation matrix was 1.5% 8 000 000 poly(ethylene oxide) (PEO) in 1× TBE buffer. An argon ion laser was used to excite the fluorescence. A CCD camera was used as the detector of laser-induced fluorescence. The whole instrumental set-up was discussed in detail.

The direct PCR from whole blood simplifies the sample preparation to a minimum. It allows fast genotyping in this application. It also has the potential to increase the speed of clinical applications, e.g. diagnosis of genetic-related diseases, and bacterial or virus typing directly from body fluids. A capillary array electrophoresis system will increase the speed and throughput of analysis. The on-line combination of the direct PCR from whole blood and capillary array electrophoresis shows an automated system for genotyping from blood to final result without any middle handling. Future total automation by computer control was also discussed.

### Microchip assays for enzyme kinetic analyses

*Claudia B. Cohen, Theo D. Nikiforov and J. Wallace Parce, Caliper Technologies Corporation, 1275 California Ave., Palo Alto, CA 94304, USA*

Microfluidic biochemical processing systems promise to replace traditional bench top analyses and biosensing devices. Electrokinetic fluid transport in interconnected channel structures allows for the complete automation of complex biochemical analyses. Herein, we describe kinetic enzymatic analyses performed in a microfabricated device: a microchip. Once the chip is loaded with the chemical species of interest, no fluid handling is required. All reagent mixing and monitoring is performed through the computer interface. Fluorescence microscopy is implemented for sensitive detection in nanoscale volumes. Small reagent volumes, fast analysis times, and flexible on-chip calibration and control experiments provide easy, quick and accurate results.

Continuous flow on-chip enzyme assays for three classes of enzymes are presented. Enzymes with turnover rates varying by three orders of magnitude are studied on the same microfluidic device. The mechanism for signal production differs for each assay. Fluorogenic catalysis, suppression of resonance energy transfer quenching, and mobility shift mechanisms are utilized to enable detection by fluorescence microscopy. Changes in fluorescence as a function of substrate concentration, and the absence and presence of known inhibitors is used to determine kinetic constants for the enzymes. Excellent agreement between on-chip and traditional cuvette measurements of $K_m$, $K_i$, $V_{max}$ and $K_{cat}$ is reported. The continuous flow on-chip enzyme assay with inhibition is demonstrated for 8 h with no reagent replacement. The reproducibility and stability of this extended experiment were discussed.

### Microchip isotachophoresis/Raman spectroscopy

*Patrick A. Walker III and Michael D. Morris, Department of Chemistry, University of Michigan, Ann Arbor, MI 48109-1055, USA; Brian N. Johnson and Mark A. Burns, Department of Chemical Engineering, University of Michigan, Ann Arbor, MI 48109-2136, USA*

We describe real-time on-chip Raman spectroscopy of ions separated by microchip capillary isotachophoresis and related electrophoretic techniques. The microchip is directly compatible with most Raman microscopes, with no interfacing needed. Because of the exquisite spatial resolution inherent in Raman microprobes, separation after isotachophoretic concentration into contiguous analyte zones is often unneeded. Preconcentration by field amplification and/or isotachophoresis (ITP) easily increases ion concentrations by three–four orders of magnitude. Microchip normal Raman spectroscopy from initially ultradilute solutions is possible, although Raman scattering is an intrinsically weak effect.

Fluorescence problems common to normal Raman spectroscopy, which are largely due to matrix impurities, are minimized because the interference is spatially removed from the analytes. In the isotachophoretic mode, detection limits depend upon the preconcentration factor, but also on the spatial resolution of the microprobe and the spectrum acquisition rate. Acquisition rates of 4–10 spectra/s are adequate.

We illustrated these advantages and concepts using environmentally interesting pesticides, paraquat and diquat. We showed the advantages of simple chemometric procedures for reduction of detection limits by resolution of overlapped spectra in interdiffusion zones.

### Liquid chromatography with on-line oestrogen receptor detection: selective determination of oestrogenic compounds

*Aaike J. Oosterkamp and Hubertus Irth[1] Department of Medical Bioanalysis, IIBB-CSIC, c/ Jordi Girona 18-26, 08034 Barcelona, Spain; [1]ScreenTec, P.O. Box 9502, Einsteinweg 55, 2300 RA Leiden, The Netherlands*

Xenoestrogens are natural or man-made compounds that disrupt the endocrine system by interacting with the oestrogen receptor. Many xenoestrogens have already been identified, and it is believed that more yet unidentified compounds can interact with the oestrogen receptor. Thus, for environmental analysis, methods are needed that can identify known and unknown oestrogenic compounds.

A novel approach is the use of biochemical assays, e.g. receptor assays as a tool for detection in liquid chromatography. This technique assay combines the separation efficiency of LC with the selectivity and sensitivity of biochemical assays, and can be used in areas where both LC and receptor assays are used, e.g. the discovery of receptor binding ligands in complex samples.

In this presentation, the development of an on-line LC-oestrogen receptor detection analysis method for the analysis of oestrogens in difficult matrices was presented. The on-line LC-oestrogen receptor detection was based on a post-column homogeneous receptor assay. In this assay, the oestrogen receptor was added to the LC effluent to react with eluting oestrogenic compounds. In a second step, a fluorescent oestrogen, coumestrol, was added to react with the remaining binding sites of the oestrogen receptor. Since free and receptor-bound coumestrol differ significantly in fluorescence intensity, the reaction can be monitored using a conventional fluorescence detector.

The use of this analysis method to analyse selectively oestrogens in urine and plasma was discussed. Furthermore, its application to analyse oestrogenic compounds in river water and the analysis of novel xenoestrogenic compounds were evaluated.

### The concept of 'electronic nose' technology

*Krishna C. Persaud, Department of Instrumentation and Analytical Science, UMIST, P.O. Box 88, Sackville Street, Manchester M60 1QD, UK*

Conducting polymer sensors have been developed at UMIST and other centres. Films of conducting polymers consist of polycationic chains of aromatic or heteroaromatic molecules, with the positive charges counterbalanced by anions. Conductivity of these materials may be modulated reversibly when various chemicals are adsorbed, the sensitivity being generally greater for polar molecules. Specificities of individual sensors to groups of chemicals are relatively broad, but the selectivity of individual sensor elements can be tailored by adapting the chemical structure of the polymers to the type of chemical groups to be detected. Hence, an array of sensors, when exposed simultaneously to the identical concentration of a volatile chemical, produces a pattern of responses that may be used as a descriptor of the chemical species adsorbed. This concept has been exploited in recent years by a number of companies and adapted to a wide range of sensor technologies, as well as a large number of applications. Pattern recognition methods using statistical multivariate analysis coupled to fuzzy neural networks have been applied with great success to turn a set of non-specific sensors into highly discriminating systems.

We were involved with the European Space Agency, Deutsche Aerospace, and HKR Sensor Systems, Germany, to develop an experimental system for real-time monitoring of the breathable atmosphere with the MIR Space station. A module containing 20 conducting polymer sensors and four quartz microbalance sensors was flow between September 1995 and February 1997. This module was able to monitor, in real time, the emission of volatile chemicals within the spacecraft due to normal crew activities, e.g. food preparation, cleaning and exercise, and more importantly, was able to monitor events due to faults or incidents within the missions. These included fluid leakages, fire and purity of the water supply.

Monitoring of malodorous environments, e.g. pig farms, and assessment of quality of raw materials used in the food industry are also important applications of the system. Examples were given of correlations with human panel assessments.

These developments are now being taken into the real world by AromaScan, based in Crewe, UK.

### Fibre-optic-based artificial nose for organic vapour

*David R. Walt, Todd A. Dickinson, Karri L. Michael and Sandra L. Schultz, Department of Chemistry, Tufts University, Medford, MA 02155, USA; Joel White and John S. Kauer, Department of Neuroscience, Tufts School of Medicine, Boston, MA 02111, USA*

We have developed the first fibre-optic-based 'artificial nose' for the detection of organic vapours. Inspired directly by principles of biological olfaction, the system is based on arrays of cross-reactive or semi-selective sensors in conjunction with various pattern recognition schemes. The system exploits the different temporal changes in fluorescence exhibited by a solvatochromic dye immobilized in different polymer matrices upon exposure to vapour pulses. Several different array formats have been demonstrated, from bundles of single core fibres to 400 μm diameter image guide arrays.

Recent innovations using acid-etching techniques have made it possible to form wells at the end of optical imaging fibres, into which thousands of micro-scale sensor beads can be placed. Beads are commercially available, and can be made with extraordinary uniformity and in very large numbers (billions per ml), thus facilitating the preparation of highly reproducible array sensors. In most bead applications, particularly in the field of combinatorial chemistry where large libraries of beads are often generated, different methods of encoding are needed for the subsequent identification of a particular bead, and its associated chemistry or synthesis product. Bead-encoding schemes have ranged from the use of fluorescent dyes to mass spectrometric analysis using molecular tags. In the present design, the inherent difference between the different polymer/dye beads eliminates the need for elaborate encoding techniques; because each sensor type is constructed from a different polymeric material, each bead type will have its own unique response to a particular test vapour pulse. In this way, beads are inherently 'self-encoding', and can be readily identified and categorized after their random placement into an image guide array. The redundancy created by incorporating large numbers of each bead type in the image guide not only protects the array from failure of any one sensor, but also affords signal amplification through bead-response summing. This signal averaging method has been used to significantly enhance signal-to-noise ratios, thus improving detection limits by over an order of magnitude.

### Applications of electronic nose in clinical diagnosis

*Susan S. Schiffman[1] and H. Troy Nagle[2], [1]Department of Psychiatry, Duke University Medical School, Durham, NC 27710, USA; [2]Department of Electrical and Computer Engineering, North Carolina State University, Raleigh, NC 27695-7911, USA*

Gaseous emissions from patients have been used to diagnose medical conditions since ancient time. Breath analysis, though uncommon in modern medical practice, can aid in the diagnosis of diabetes and other medical conditions. Diabetic patients can emit the odour of acetone. Odours are also associated with the skin in the onset of uremia during kidney failure. Odorous discharges are also a common feature of serious bacterial infections, as are sometimes seen in gastrointestinal maladies, vaginal discharges, traumatic wounds and pressure ulcers.

In recent years, new sensing techniques that are potentially more sensitive, objective and reliable than the human sense of smell have become available that employ arrays of gas sensors. These 'electronic nose' devices have been used in a wide range of applications including the clinical diagnosis of medical conditions. This technology promises to deliver inexpensive, portable instruments that can give instantaneous, accurate measurements at the point of care (POC). Rapid real-time measurements with inexpensive instruments can help improve treatment and reduce costs in today's healthcare environment. As healthcare service migrates from hospitals to outpatient and extended-care facilities, other applications of the electronic nose will emerge, e.g. determining how often Alzheimer's or bed-ridden patients should be washed or bathed. In this presentation, we reviewed a wide-range of potential medical applications for electronic nose technology.

### Electronic nose as a detector for gas chromatography

*Marc Breimer, Miriam Masila and Omowunmi A. Sadik, Chemistry Department, State University of New York at Binghamton, P.O. Box 6016, Binghamton, NY 13902-6016, USA*

An understanding of the chemical language of olfactory communication in mammals presents an immense challenge to modern instrumentation in terms of their sensitivity and discrimination. There has been several attempts to analyse volatile materials using headspace gas chromatography with mass spectrometry in order to search for patterns of occurrence of a range of different compounds which could determine an individual or group signature. Despite the high resolution and high sensitivity in gas chromatographic techniques, the sensitivity can not match that of the human nose. One major difficulty in interpretation is that the differences in sensitivity of the GC detector to different compounds do not correspond to those of the mammalian nose. Major chromatographic components may have little olfactory impact, and in most cases, the trace components will predominate.

In recent years, the combination of gas sensor arrays and artificial neural networks now commonly referred to as electronic noses, has provided a fast method of measuring odours by analogy with natural olfaction. While an electronic nose may provide an alternative variable that is thought to be equivalent, objective and/or unique as an indicator for the condition of interest, there may be additional information necessary to prove that this equivalence holds of all samples of interest. One would therefore have to look at alternative on-line or hyphenated laboratory methods that could be developed to compete with the mammalian olfactory system.

In this presentation, an alternative chemical imaging strategy based on the combination of GC and electronic nose was presented. Identification and quantitation using the combined gas chromatography with electronic nose (GC-EN) was described for PAHs, fatty acids, methyl esters and phenolic compounds. GC-EN should provide complementary chemical signalling and image profiling for a range of volatiles. This new information is considered as advantageous in designing artificial olfactory devices which may allow efficient and reliable monitoring of important compounds.

### Smart atmosphere monitoring by electronic nose

*Hans-Jürgen Keding, Daimler-Benz Aerospace, Richard-Wagener-Str. 31, D-18119, Rostock, Germany*

During the last five–seven years, so-called 'olfactory sensing systems' have been developed and are commercially available, which are based on the idea of simulating the olfactory system of humans. Such sensor types use arrays of transducers, which need not be highly specific to individual molecules, but which are slightly different and provide overlapping characteristics. An array of several transducers provides electrical response patterns to any mix of gasses which are typical for each gas-mix. Thus, 'signatures' in the form of patterns representing the 'odour' of the gas-mixture have to be analysed, for which pattern-recognition techniques are used. Different measurement systems based on multi-sensor arrays and pattern recognition technologies have been developed and implemented by the Daimler Benz Aerospace Rostock for the detection and assessment of complex gas mixtures.

A 'Smart Gas Sensor' has been realized in response to an order of the European Space Agency based on the usage of two different sensor arrays (quartz crystal oscillators, semi-conducting polymers). The measurement system has been used on the MIR space station with the objective to monitor the atmosphere in the spacecraft against a broad variety of contamination, and to identify contamination and hazardous events. The results of the mission confirmed that the 'Smart Gas Sensor' provides a fairly complete mapping of the atmosphere changes within the closed loop system of a spacecraft, and that the equipment can be used to identify non-nominal events which may be affecting the crew or spacecraft.

The analyser 'SamSelect' is a portable and modular device consisting of a multi-sensor module, a headspace autosampler and a reference gas generator. Due to the modular concept, the usage of different sensor technologies is possible (quartz microbalance – surface acoustic wave – metal oxide – semi-conducting polymer sensors). A separate computer has to be used for the evaluation of the sensor signals. The software contains data acquisition, data evaluation and data archiving tools. An integrated report assistant realizes an automated generation of measurement protocols.

Fire and other hazardous events with release of volatiles can be detected in an early phase by the monitoring of a broad variety of off-gassing substances and products of thermal digression. The smart pre-fire detector 'SamDetect' is a compact measurement system which consists of a six-fold sensor array made from MOS-sensors and a micro-controller for data acquisition, data processing and system control. Gas signatures measured by the multisensor array were analysed by smart data evaluation procedures in order to detect the cause of the hazardous event, to classify the burning substances and to set an alarm with low false alarm probability.

### Improved elemental analysis with electrospray mass spectrometry via on-line electrochemical sample pretreatment

*Jack R. Pretty and Gary Van Berkel, Chemical and Analytical Sciences Division, Oak Ridge National Laboratory, Oak Ridge, TN 37831-6375, USA*

The potential advantages of electrospray mass spectrometry (ES-MS) for speciation of inorganic analytes have long been recognized. However, a number of practical limitations have made it extremely difficult to apply this approach to real samples. These include relatively poor detection limits for most inorganic analytes (as compared with competitive detection systems, e.g. ICP-MS), matrix-dependent changes in the gas-phase ionization efficiency of the analytes, and the effects of sample matrices on fundamental aspects of the ES-MS process itself (e.g. the influence of changing solution conductivity).

Preconcentration of analyte to improve detection limits with simultaneous separation of analyte from sample matrices detrimental to ES-MS operation, would address many of the aforementioned limitations. In earlier research, the utility of on-line stripping voltammetric flow systems for improved detection via ICP-MS has been demonstrated. Substantial signal enhancements (one to two orders of magnitude) have been achieved for a wide variety of electrochemically responsive inorganic analytes, including various transition metals and uranium, while total elimination of high levels of matrices problematic in ICP-MS but electrochemically unresponsive may be conveniently and rapidly accomplished using such a system.

We are exploring the use of on-line stripping voltammetry flow systems for improved analysis of inorganic species with ES-MS detection, to provide analyte signal enhancement via preconcentration and matrix elimination via electrochemical selectivity. Particular requirements for interfacing such systems with ES-MS, including choice of cell materials, solvents and electrolytes, and the necessity for electrical decoupling were addressed. The convenient transfer of inorganic analytes from media wholly unsuited to ES-MS operation to suitable media (e.g. from acidic aqueous solutions into methanol or acetonitrile) was demonstrated, and quantification results for samples problematic in ES-MS, e.g. artificial and natural seawater, were discussed.

### The preliminary screening of trace organic contaminants in foodstuffs by gas chromatography plasma source mass spectrometry

*Gavin O'Connor, Steve J. Rowland and E. Hywel Evans, University of Plymouth, Department of Environmental Science, Plymouth PL4 8AA, Devon, UK; Christopher Wright, CSL Food Science Laboratory, Norwich Research Park, Colney, Norwich NR4 7UQ, UK*

The identification and quantification of trace organic contaminants in foodstuffs is an on-going area of research for many governmental bodies. The continued use of pesticides, process chemicals, additives and food cosmetics has led to a vast increase in the variety of organic contaminants finding their way into the food chain. Gas chromatography coupled to flame ionization detectors and electron capture detectors have been used for many contaminants, however, the most widely applicable detector is mass spectrometry. This provides qualitative information about the analyte, and trace and quantitative analysis can be performed using selected ion monitoring. However, this requires the operator to be specific about the compounds being analysed. Atomic emission spectrometry coupled to GC has been investigated for preliminary screening of halogenated compounds. In an effort to improve detection limits for preliminary screening, plasma source (PS) MS has been investigated. The problems associated with conventional argon inductively coupled plasma mass spectrometry, e.g. low ionization energy for halogens and the polyatomic interferences below mass 80 m/z, have been addressed by replacing the ICP with microwave-induced plasma and low pressure plasmas, and these sources have been investigated for the preliminary screening of priority contaminants. This was done by element-selective monitoring at ultra trace levels. Comparisons between the GC-PS-MS methods and conventional GCMS methods have been made. Sample clean-up, preconcentration and preparation for the removal of possible interferences, for the instruments, were discussed, along with overall analysis time and detection limits, for spiked foodstuffs.

### *In situ* determination of aqueous pesticides with a single measurement, fibre optic excitation-emission matrix fluorometer

*Renee D. Jiji, Gary Cooper and Karl S. Bookish, Department of Chemistry and Biochemistry, Arizona State University, Tempe, AZ 85287, USA*

Fluorescence spectroscopy is an attractive alternative to chromatographic techniques for determination of pesticides in groundwater. Many pesticides are naturally fluorescent and others photodegrade into fluorescent products. Two principal disadvantages associated with fluorescence spectroscopy are overcome by combining instrumentation advances with chemometric data analysis. An entire excitation-emission matrix fluorescence spectrum is imaged on a CCD chip and analysed in conjunction with previously collected standard spectra by 'three-way' methods, e.g. Parallel Factor Analysis (PARAFAC) and Direct Trilinear Decomposition (DTLD). PARAFAC and DTD-based analysis of the collected EEM data permit mathematical resolution of the analyte signature from most fluorescent backgrounds. Decomposing EEM data with three-way methods alleviates the need to find a single excitation and emission wavelength set that is unique to the analyte of interest. Furthermore, collection of single measurement EEM fluorescence spectra is quicker than collection of scanning EEM fluorescence or synchronous fluorescence for a given signal to noise level. Application of a fibre-optic-based, single measurement spectrophotometer for determination of targeted naturally fluorescent pesticides in ground water was demonstrated. Particular attention was aimed at the ability to resolve overlapping chemical signatures to perform accurate analyses in complex environments. The speed, accuracy and detection limit of EEM fluorescence were compared to that of chromatographic methods following solid phase extraction.

### Metals monitoring for process control of laser-based coatings removal

*Mark E. Fraser, Amy J. R. Hunter and Steven J. Davis, Physical Sciences, 20 New England Business Center, Andover, MA 01810, USA*

Cost-effective and environmentally sound means of paint and coatings removal is a problem spanning many government, commercial, industrial and municipal applications. For example, the Department of Energy is currently engaged in removing paint and other coatings from concrete and structural steel as part of decommissioning former nuclear processing facilities. Laser-based coatings removal is an attractive new technology for these applications as it promises to reduce the waste volume by up to 75%. To properly function, however, the laser-based systems require some form of process control.

Physical Sciences Inc. (PSI) is developing a feedback control technology for laser-based coatings removal systems by monitoring emissions from the laser/surface interaction. This approach is preferable to other alternatives as the degree of surface cleanliness is best characterized by direct measurement of primary and containment species in the plume. The PSI monitor is an enabling technology that permits the laser systems to optimize the economics of the cleaning process, reduce worker exposure, minimize cleaning time, identify the hazard level of the waste, and decrease the extent of post-surface characterization. The monitor readily distinguishes the lead paint stripes from the non-lead stripes.

For this DoE-sponsored programme, PSI is teaming with a laser cleaning system developer and national laboratory. The programme has already successfully demonstrated a monitor for lead for application to lead-based paints. Work in progress includes permanent integration of the technology into a laser system, and an upgrade to detect uranium and plutonium, important trace contaminants in DoE remediation operations.

### Microwave-assisted sample preparation and high speed multicapillary gas chromatography: a high throughput alternative for organotin speciation in solid samples

*Vincent Schmitt, Isaac Rodriguez Peirero and Ryszard Lobinski, Laboratoire de Chimie Bio-Inorganique et Environement, Centre Helioparc, 2 Ave du president Angot, F-64000 PAU, France*

Speciation of organometallic compounds released into the environment as a result of an anthropogenic activity has been attracting considerable attention in recent years. The analysis time and sample throughput have long been controlled by the length of the sample preparation step, which has traditionally been complex, multistep and time consuming.

The use of microwave field is demonstrated to shorten the time of sample preparation to a few minutes. This is now the duration of the GC run that is the virtual bottleneck of a speciation analysis. Indeed, the time needed for the whole separation by conventional capillary column of the most volatile ($SnEt_4$) and least volatile ($Ph_3SnEt$) of the organotins is about 25 min. Multicapillary column (MC) coupled to a microwave-induced plasma atomic emission detection (MIP-AED) was then developed to shorten the organotin speciation analysis to 2.5 min.

Influence of carrier and plasma gas flow rates, oven temperature, and injection volume onto the compatibility of MC with MIP-AED was discussed. Validation of the MC/MIP-AED after microwave-assisted sample preparation for the analysis of sediment (PACS-1 and BCR-462) and biological (NIES-11) certified reference materials was also presented.

### Direct mercury analysis with field capabilities: new EPA method 7473

*Helen M. Boylan, Peter J. Walter and H. M. 'Skip' Kingston, Department of Chemistry and Biochemistry, Duquesne University, Pittsburgh, PA 15282, USA*

A new analytical method that does not require sample pretreatment has been developed for the determination of total mercury in solid and aqueous samples. With EPA Method 7473, laboratory and field analysis of mercury in solids and solutions by thermal decomposition, amalgamation, and atomic absorption spectrometry, mercury in solids or solutions is measured in an analysis time of less than 5 min. Method 7473 has passed the preliminary review by the EPA and has been released for peer review.

Method 7473 is based on the instrumental methodology used by the DMA-80 mercury analyser (Milestone). Automated processing controls drying, and thermal and chemical decomposition of the sample. Mercury vapour is selectively trapped on a gold amalgam before release to the absorbance cells. While the fundamental theory behind this instrumentation has been available in the literature, this is the first instrumental implementation of these integrated concepts.

Validation of Method 7473 has been performed by analysis of various National Institute of Standards and Technology Standard Reference materials (SRMs) in the laboratory and in a field environment. The results agree with the certified values in both settings. Precision was evaluated using a local soil that was homogenized and analysed in triplicate. RSDs less than 10% were confirmed.

In collaboration with a large US corporation, this methodology is currently being used to test soil samples from mercury-contaminated sites. The results are being validated with concurrent analyses at a contract laboratory using independent analytical methods. Subsequent to valid in-laboratory examination of the soils, the method will be applied on-site in field studies. Method 7473 has potential for species-specific analyses and full site characterizations, but is not limited to environmental applications. Method validation data, 'real-world' solid results and potential applications for Method 7473 were presented.

### Evaluation of photoacoustic spectroscopy for quality control of cement

*Stanley J. Bajic*[1], *Glenn A. Norton*[1] *and John F. McClelland*[1,2], [1]*Ames Laboratory, Iowa State University, Ames, IA 50011, USA;* [2]*MTEC Photoacoustics, P.O. Box 1095, Ames, IA 50014 USA*

Cement failures, e.g. cracking, are frequently the result of variability in cement composition and can be minimized by analytical screening of the cement prior to hydration. A quick, accurate and non-destructive technique is needed to determine cement components prior to hydration. This paper will demonstrate the feasibility of using infrared-photoacoustic spectroscopy (PAS) as a viable technique that can quickly provide information on cement composition. Infrared spectroscopy provides a 'fingerprint' of the cement's composition, which then can be used to predict its performance. One particular area of interest is the sulphate mineral species present in the cement. For example, it is desirable to have the sulphate present as gypsum ($CaSO_4 \cdot 2H_2O$) rather than bassanite ($CaSO_4 \cdot 1/2H_2O$), anhydrite ($CaSO_4$) or arcanite ($K_2SO_4$).

The PAS technique is of interest because the cost is much lower than for other types of equipment (e.g. thermal analysers and X-ray diffractometers) frequently used for mineral analyses, it requires virtually no sample preparation, and can perform multi-component analyses in a matter of only several minutes. Strengths and limitations of the PAS approach for the mineralogical analyses of interest were identified and the utility of the technique for routine cement analysis was discussed.

### Identification and quantification of polycyclic aromatic sulphur heterocycles (PASH) in different fossil fuels using GC-AED and GC-MSD

*Stephanie G. Moessner and Stephen A. Wise, Analytical Chemistry Division, National Institute of Standards and Technology, Gaithersburg, MD 20899, USA*

An analytical method is described for the separation, identification and quantification of polycyclic aromatic sulphur heterocycles (PASH) in different fossil fuels, including two standard reference materials, SRM 1597 (Coal Tar) and SRM 1582 (Petroleum Crude Oil), as well as a number of different decant oils and crude oils. The compounds of interest were dibenzothiophene, methylated, dimethylated and trimethylated dibenzothiophenes, the three benzo[*b*]naphthothiophenes, as well as all 30 possible methyl-benzol[*b*]naphthothiophenes.

Oil is a very complex matrix, partly as a result of the excess of PAH compared to PASH. Therefore, the analytical method requires a number of necessary prerequisites: effective sample clean-up, selective stationary phases, as well as sensitive and selective methods of detection.

The clean-up involves sample preparation steps using aminopropylsilane solid phase extraction cartridges with different solvent mixtures followed by normal-phase liquid chromatographic isolation of the PASH based on the number of aromatic carbons. These aromatic ring fractions were then separated by high resolution gas chromatography using three different stationary phases with different selectivities (DB-5MS, DB-17MS, SB-Smectic). Quantification results achieved by high resolution gas chromatography using mass selective detection (GC-MSD) and atomic emission detection (GC-AED) were compared. In addition, various isomeric ratios of a number of alkyl-substituted dibenzothiophenes and benzo[*b*]naphthothiophenes, possibly due to differences in maturity and location, were evaluated.

### Overview of new developments in preparative chromatography

*R. Eric Gerber and Richard Hatch, Varian Chromatography Systems, 2700 Mitchell Drive, Walnut Creek, CA 94598, USA*

Does achieving highly efficient separations require small particle ($\leq 8\,\mu m$ average diameter) packing materials, specialized equipment and techniques that are significantly different than those familiar to most users of HPLC? Axial compression column packing hardware was tested for the preparation of highly efficient columns using silica-based packing materials. The effects of mobile phase temperature control were studied over the range of 14–24°C, and column performance at various flow rates and pressures was recorded. With the proper column packing and mobile phase pumping equipment, we have found that columns with efficiencies of 40 000 to well over 100 000 plates/m can be easily prepared. This presentation illustrated the use of small particle packing materials in a variety of column configurations from 41.4 to 152 mm i.d. Unusual considerations, e.g. the need for stringent temperature control of mobile phases, consideration of sample displacement effects, and the postseparation processing of purified fractions were discussed. When all critical factors are controlled, true high performance liquid chromatography can now be practiced at scale-up factors of at least 1000 from typical analytical separations.

### Monitoring air quality on spacecraft by open path FTIR

*Melissa D. Tucker, Rebecca C. Rowe, Ellen V. Miseo and James R. Valentine and Arthur D. Little, Cambridge, MA 02140, USA*

Monitoring the air quality on a spacecraft is essential to assure that major and trace constituents are at levels appropriate for sustaining life and conducting planned experiments. Ambient air must be monitored throughout the cabin, especially in areas where air is relatively stagnant. The air quality must be characterized in terms of specific major and trace species concentrations, and their rates of change.

The National Aeronautics and Space Administration (NASA) is currently defining environmental monitoring and control requirements for human space missions. NASA is also developing a suite of sensing technologies that meet the requirements established. Spacecraft maximum allowable concentration (SMAC) levels have been established for major and trace constituents anticipated to be found in the spacecraft environment.

The characteristics and capabilities of open path Fourier transform infrared (OP-FTIR) spectrometry were evaluated for application to the measurement of trace contaminants in spacecraft air systems. The results of this evaluation suggest that OP-FTIR has the potential to provide simultaneous, near real-time quantification and confirmation of identity for most contaminants on the current SMAC list. In addition, using an open-path measurement mode may afford a unique means to monitor the composition and distribution of air contaminants throughout spacecraft air systems without the need for multi-point sampling. The open-path monitoring mode permits the flow of the ambient atmosphere through the optical path.

Most organic and inorganic compounds have unique vapour-phase infrared signatures that can be observed for concentrations from the low ppm to high ppb levels. For this preliminary assessment, a commercially available infrared spectral library was used which contained over half the compounds on the SMAC list. Two complementary pathlengths were identified to be optimum for monitoring and to assure that most compounds would be observed at the SMAC exposure limits. The combination of pathlengths offering the most thorough analysis of the SMAC compounds is a 15–20 cm path and a 25–40 m path. Multiple paths can be covered using a single instrument and reflective optics or multiple instruments. Measurement along an array of paths and subsequent analysis of these paths allows for tomographic reconstruction of the spatial distribution and dynamic flow characterization of the air constituents and contaminants.

### Measurement of chromium speciation changes in *in vitro* bio-availability assays by IC-ICP/MS

*Brian Buckley, Scott Skorupsky, Willie Johnson, Wei Fang and Ikhlas Rashid, Environmental and Occupational Health Sciences Institute, Rutgers University, 170 Frelinghuysen Road, Piscataway NJ 08854, USA*

The fraction of a metal contaminant found in a soil or waste matrix that can be incorporated into the human has usually been estimated by animal feeding studies and the subsequent analysis of the tissue. This is an expensive and potentially inaccurate means of performing this assay. An inexpensive alternative model is being developed to used human bio-fluid surrogates to extract the waste material. Preliminary data suggest these surrogates can provide more than the total fraction of bio-available metal contaminant. It is possible to use this model as a means to elucidate the mechanisms involved with the complexation and de-complexation of the metals with the organic substituents found in the bio-fluids.

Many of the substituents found in the biological fluids can complex or reduce Cr (VI) to the carcinogenic form. Speciation by IC-ICP/MS has allowed us to isolate these complexing agents. Initially chromatographic data suggested that some of the chromium remained in a complexed carcinogenic form after extraction with the gastric juice. Subsequently, the complexation process appeared to have been reversed as the extraction fluid changed from the gastric juice to the jejunal fluid. Varying the chromatographic conditions suggests other possibilities exist.

This presentation focused on the isolation and identification of the complexing agents, and the reversal of the complexation process. The speciation method used to study the chromium as its species changes during the synthetic bio-fluid extraction process was also described. The bio-available/bio-accessible assay method was also presented, as well as data on the fraction of the metal contaminants thought to be bio-accessible.

### Determination of trace elements in urine by flow injection hydride generation electrothermal atomic absorption spectrometry

*Julian F. Tyson, Nusret Ertas and Robert I. Ellis, Department of Chemistry, Box 34510, University of Massachusetts, Amherst, MA 01001-4510, USA; Susan McIntosh and Frank Fernandez, Perkin-Elmer Corporation, 761 Main Avenue, Norwalk, CT 06859, USA*

A flow injection, hydride generation electrothermal atomic absorption spectrometry (FI-HG-ETAAS) procedure for the determination of total selenium in urine has been developed (*Spectrochimica Acta Part B*, in press) which utilizes an off-line open vessel pretreatment with bromine, generated from hydrobromic acid and potassium bromate, to convert all species to inorganic selenium(iv), the required precursor for formation of dihydrogen selenide. On-line sample pretreatment would simplify this procedure. Our experiments show that some organic selenium compounds are relatively easily determined by FI-HG-ETAAS from a urine matrix using online focused microwave digestion. Selenocysteine, and selenomethionine are 100% recovered. However, recoveries of selenopurine and trimethylselenonium are only 40 and 10%, respectively. The limit of detection for selenium in human urine using this procedure is around 10 ppb. The use of alternative reagents, e.g. persulphate, will be presented. Lead has also been determined by a FI-HG procedure which incorporates an oxidizing agent, ferricyanide and quartz tube atomization. An accurate analysis of NIST SRM2670 (toxic metals in urine) has been obtained at both the 10 and 100 ppb concentrations. The limit of detection is 80 ppt. A method is currently under development for the determination of lead in the urine of patients undergoing chelation therapy. It is expected that the presence of a chelating agent will cause a depressive interference in the HT procedure and that additional pretreatment will be needed to release the lead prior to generation of the volatile hydride.

### Determination of mercury in biological samples at ultra trace levels by cold vapour atomic fluorescence spectrometry

*Reshan Fernando[1], Phyllis Elkins[1], Michelle Lang[1], Robert W. Handy[1] and Bradley J. Collins[2], [1]Research Triangle Institute, 3040, Cornwallis Road, RTP, NC 27709; [2]National Institute of Environmental Health Sciences, P.O. Box 12233, Mail Drop A2-02, NC 27709, USA*

In support of a toxicological study of mercury (Hg) in rats, analytical methods were developed and validated for quantitating total mercury in a variety of rat tissues, including kidney, liver, lung, red blood cells, plasma, implants, ovary, uterus, brain and milk at parts-per-trillion level. The developed methods have been optimized to use sample sizes as small as 0.050 g of tissue.

The digestion procedure involves treatment of homogenized biological tissues with concentrated nitric and sulphuric acids in a sealed vessel at elevated temperature in a muffle furnace. After digestion, samples were further treated with $KBr/KBrO_3$ and hydroxylamine hydrochloride, and diluted to volume with deionized water. For a number of tissues, a pre-digestion step was included in the digestion scheme, where a portion of acid was added to the sample and was allowed to sit at room temperature overnight. Digested samples were treated with tin(II) chloride and analysed for mercury by atomic fluorescence spectrometry.

The validated methods have been used successfully for the determination of total mercury in both adult and neonatal tissues collected from dosed and control animals. Dosed animals were exposed to mercury vapour at four different concentrations; 1, 2, 4 and 8 $mg/m^3$ by nose-only, and control animals were exposed to conditioned air at the same flow rate as animals receiving mercury vapour.

Analysis results have indicated good correlation between the total mercury found in tissues of animals exposed to mercury vapour and the level of exposure. Method validation and sample analysis results were presented in detail.

### Determination of arsenic in air particulates, house dust and soil samples at ultra trace levels by vapour generation atomic fluorescence spectrometry

*Reshan Fernando[1], Preston Snee[1], Edo D. Pellizzari[1], Kent Thomas[1] and J. Quackenboss[2], [1]Research Triangle Institute, 3040, Cornwallis Road, RTP, NC 27709, USA; [2]US EPA National Exposure Research Laboratory, Las Vegas, NV 89193, USA*

As part of the National Human Exposure Assessment Survey (NHEXAS) program, methods have been developed for determination of arsenic in a variety of sample matrices, including aerosol, house dust and soil. The types of aerosol samples collected include personal, indoor and outdoor samples. Personal aerosol samples were collected using a specifically designed personal sampling system consisting of a total inspirable aerosol sampling inlet containing a 25 mm diameter, 3 µm porosity Gelman PTFE filter as the substrate. The aerosol sampling packages for collection of indoor and outdoor aerosol samples from fixed location inside and outside of the participant homes incorporate the same components as the personal sampling system. Wipe samples were collected by the Lioy–Weinman–Weisel (LWW) method using a template with 150 $cm^2$ area in homes and by the wet wipe towel (WWT) method using moistened polyethylene filters. One sweep dust and one soil sample were collected from each of the participant homes selected.

Aerosol, house dust and soil samples were extracted with nitric acid, and the total arsenic measured using a vapour generation atomic fluorescence spectrometry. The soil samples were processed prior to extraction by sieving through a 300 µm screen. Method performance evaluation and sample analysis results for arsenic in these different media were presented.

This work has not been subjected to Agency review and therefore does not necessarily reflect its views.

### On-line Raman spectroscopy of peptides preconcentrated by capillary isoelectric focusing

*Karen A. Reshni, Patrick A. Walker III and Michael D. Morris, Department of Chemistry, University of Michigan, Ann Arbor, MI 48109-1055, USA*

We demonstrate on-line Raman spectroscopy of peptides concentrated by capillary isoelectric focusing (cIEF). The technique requires only conventional electrophoretic apparatus with no or only minor modification to the Raman microprobe which serves as the detector.

Raman detection offers unique advantages as a capillary isoelectric focusing detector. It is instrumentally simple, and provides detailed and characteristic spectroscopic signatures. Carrier ampholytes do not interfere. They form their own quasi-continuous zones which are in any event spectroscopically easily distinguishable from peptides. Because distinct zones are formed, the fluorescence problems common to normal Raman spectroscopy with 532 nm excitation, which are largely due to matrix impurities, are minimized or completely eliminated.

We described the separation of representative renal peptides, including angiotensin II and several variant forms. These are separated according to their isoelectric points (pl) along a pH gradients generated by carrier ampholytes. We employ a two-step mode of isoelectric focusing.

The peptides are focused and then cathodic mobilization transports them through the Raman detection window. The focused peptides are concentrated approximately 200–300× to very pure zones. Raman spectra of the peptides (532 nm excitation, 600–2000 $cm^{-1}$ observation), which are concentrated to at least millimolar levels, are typically collected at 3/s. This collection rate is generally fast enough to avoid resolution loss or missed bands of minor components. The multivariate data reduction techniques which we have applied to isotachophoresis/Raman spectroscopy are used with similar success in isoelectric focusing.

### Applications of *in situ* spectrometric measurements for decommissioning surveys

*Kevin M. Miller, Environmental Measurements Laboratory, U.S. Department of Energy, New York, NY 10014–4811, USA*

Rapid and cost-effective methods for measuring radioactive contaminants are needed to support the decommissioning of various governmental and commercial facilities throughout the country that have been associated with the nuclear weapons complex or the nuclear fuel cycle.

As a method that has been well established for assessing radionuclides in the environment from fallout, *in situ* spectrometry is now finding application in site characterization before, during and after remediation work. By directly measuring the photon fluence rate in the field and relating it to source strength, this technique provides non-intrusive, nuclide-specific concentration or deposition levels in soils within minutes for many radionuclides at levels within the range of release limits. Significantly, this technique can be used for the final status or certification survey as the information generated is well suited to answering the fundamental question as to whether the average levels of residual radioactivity within a survey unit meet the release criteria.

In areas where there is a highly heterogeneous radionuclide distribution, an uncollimated *in situ* spectrum provides a centre-weighted averaging effect over its field of view. Thus, a single measurement leads to a far more representative concentration value than a single soil sample. For situations where a more complete picture of contaminant levels is desired, measurements can be spaced such that an overlap results in the detector field of view. Deconvolution techniques can then be applied to produce either a smoothed distribution of concentrations or one that maximizes potential peaks hidden in the data set.

For radionuclides that have been driven into the surface soil by natural processes or other physical disturbances, *in situ* spectrometry is a robust technique that gives representative concentrations despite variations in terrain and oil make-up. The area of soil being viewed can be adjusted with detector height, while the depth being viewed is related to the energy of the gamma ray being analysed.

*In situ* spectrometry has been included as an optional measurement tool in the Multi-Agency Radiation Survey and Site Investigation Manual and has been applied to several demonstration surveys performed for the Nuclear Regulatory Commission in support of the new decommisioning rulemaking. Within the Department of Energy, the soils certification programme at the Fernald Environmental Management Project is implementing *in situ* spectrometry with a considerable cost savings projection over standard soil sampling and laboratory-based analyses.

### Automatic analysis of pollutants in the atmosphere at sub ppb levels

*J. N. Driscoll, R. Koch and E. S. Atwood, HNU Systems, 160 Charlemont St., Newton, MA 02161, USA*

Hydrocarbons along with oxides of nitrogen and ultraviolet light combine in the atmosphere, in a complex manner, to form photochemical smog. To make matters worse, automotive sources emit several hundred different hydrocarbons. Over the past decade, aromatics have replaced tetraethyl lead in gasoline as octane enhancers, thereby increasing the emissions of aromatic hydrocarbons, e.g. benzene, toluene, ethyl benzene and xylene (BTEX) from automotive sources. There is a definitive need to monitor these species in the atmosphere at low or sub ppb levels. Our approach has been to design an automatic GC that employs the HNU photoionization detector (50 times more sensitive than a FID for aromatics) and a concentrator. This instrument has the advantage of sensitivity, specificity [fewer interferences than the flame ionization detector (FID)], no flame to blow out, and no requirement for cylinders of hydrogen and air (or no expense for hydrogen generators).

When we ran tests on the UHP nitrogen gas cylinders, we found significant (ppt) levels of these aromatic compounds in the gas (toluene, 50 ppt and xylene, 200 ppt). Typical zero gas has a specification of $< 0.1$ ppm of hydrocarbons. While this is adequate for most studies, it is a serious problem then one is analysing low ppb or ppt levels of gases. The instrument was calibrated and allowed to run for seven days on ambient air. The same standard (high ppb level) was then analysed under identical conditions. The results of this analysis showed that the stability of the device was excellent (within $\pm 1\%$). Compounds, e.g. benzene, toluene and *m*-dichlorobenzene showed a small change after seven days, about 15% or less, while the results for the *o*-xylene were somewhat higher, *ca.* 24%. The stability of the retention time during the time period was excellent ($< 0.2\%$ relative). The reproducibility [coefficient of variation

(CV)] of the standard low ppb gas for five sequential analyses varied from 4 to 7.7%.

### Direct measurement of methane and non-methane hydrocarbons in vehicle exhaust by flash gas chromatography

*Stephen J. MacDonald, Thermedics Detection, 220 Mill Road, Chelmsford, MA 01824; Morgan Lapan, Chelsea Proving Grounds, Chrysler Corporation, 3700 South State Road, 52, Chelsea MI 48118-9691, USA*

The measurement of methane and non-methane hydrocarbon (NMHC) levels in vehicle exhaust is an important step in the characterization of engine performance. Present methods measure the total hydrocarbon (HC) content and the methane content independently, and then subtract the methane from the total HC to determine NMHC levels. Direct measurement of the NMHC is more accurate than subtracting the methane from the total HC. Measurements are typically done during engine start up and warm up conditions, and take longer than 5 min. Faster analysis provides more timely data and allows more samples to be taken during the engine warm up stage.

A method was devised to directly measure the methane and NMHC content of vehicular exhaust using Flash GC. Flash GC techniques employ resistive heating of a metal tube containing a capillary GC column to attain temperature programming rates in excess of 30°C/s. Two capillary columns of different GC phases were used with two six-port rotary valves to automatically sample, separate and quantitate the methane and NMHC with a cycle time of 40 s. The accuracy and precision of system performance, along with detection limits and applicability, were discussed.

### Novel, automated, sample handling approach to deliver faster results and increase sample throughput of environmental labs

*Fausto Pigozzo, Pietro Mapelli, Albino Sironi, Sorin Trestianu and Jessie Butler, CE Instruments, Strada Rivoltana, I-20090 Rodano, Milan, Italy*

A novel, automated GC technology has been developed to improve productivity of environmental laboratories. Most of the standardized methods applied for the determination of organic pollutants in soil or water prescribe sample confirmation procedure with different polarity columns to resolve 'critical pairs' of closely eluting peaks. In some cases, even different GC detectors are required to provide specific and positive identification.

The conventional GC procedure involves separate injections of the same sample into two different analytical channels, usually two gas chromatographs, therefore doubling analysis costs and the time involved to characterize the sample.

This paper describes an automated approach that permits a single GC to do the work of two. Using a novel, automated system capable of accessing two inlets in a single shot, the DoublePro system, the same sample can be sequentially injected and analysed at the same time, with two different analytical channels using a single, optimized, GC program. This reduces the cost per analysis, delivers faster results and increases sample throughput. This solution has proved to be very beneficial to environmental laboratories in the following occasions:

- simply cutting 50% of the total analysis time and power consumption of labs performing hundreds of samples per day. This is achieved using two identical analytical channels run under the same unattended GC conditions, therefore providing the output of two samples from one instrument.
- reducing the time spent for confirmation procedures. In this case the same sample is sequentially injected and analysed at the same time, with two different analytical channels (e.g. two columns of differing polarity used for chlorinated pesticides) using a single, optimized, GC program.
- eliminating the needs of an additional GC unit in case of quick response from samples requiring different detectors for specific or positive identification. This is the case of the determination of organochloro, nitrogen and phosphorus pesticides in ground water using a double channel GC, automated with an AS 2000 Autosampler, and equipped with an electron capture detector (ECD) and nitrogen phosphorus detector (NPD).

Results are shown and discussed for each case. The system ensures a high reproducibility of retention items, and an unbeatable degree of accuracy and precision even under the extremely demanding performance criteria such as those imposed by CLP pesticides analysis.

### Asbestos monitoring—regulatory overview

*Michael E. Beard, ASTM Subcommittee D22.07 on Asbestos, 40004 Brewster Drive, Raleigh, NC 27606, USA*

Asbestos is a generic term applied to a wide variety of naturally occurring mineral silicates which are separable into fibres. The fibres generally possess high tensile strength, good thermal and electrical insulation properties, and good chemical resistance. Asbestos is a versatile fibre which has been widely and effectively used for fireproofing, insulation, soundproofing, asbestos concrete materials, friction materials, roofing and flooring materials, and a variety of industrial products. However, when asbestos fibres become airborne and are breathed into the lungs, serious adverse health effects may develop. Workmen subjected to prolonged exposure to asbestos fibres have developed asbestosis, lung cancer and mesothelioma. Government agencies have declared asbestos to be a carcinogen and have implemented regulations to prevent exposure. The Occupational Safety and Health Administration (OSHA) and the U.S. Environmental Protection Agency (EPA) have developed regulations requiring asbestos monitoring to protect workers and the general public from exposure hazards. OSHA regulates airborne levels of asbestos in the workplace and requires the identification of asbestos containing materials in buildings to control potential exposure to workers. EPA regulates exposure to asbestos in schools and emissions of asbestos to the atmosphere from mining and manufacturing, and from the renovation and demolition of buildings. Exposure to asbestos reentrained from settled dust has recently become a concern for many building owners, occupants and homeowners. No federal regulations or monitoring methods have been promulgated to deal with this potential source of exposure to asbestos. Concerned occupants and building owners frequently resort to litigation to deal with real or perceived exposure hazards from this source of asbestos. The American Society for Testing and Materials (ASTM) is developing methods and strategies for monitoring asbestos in various media, including settled dust.

The OSHA and EPA regulations rely on a variety of analytical methods to monitor asbestos. Asbestos in building materials is analysed using polarized light microscopy (PLM), X-ray powder diffraction (XRD), and/or transmission electron microscopy (TEM). Airborne asbestos is monitored using phase contrast microscopy (PCM) or TEM. Asbestos in settled dust may be monitored using PLM, scanning electron microscopy (SEM) or TEM. In general, large fibre bundles occur in workplace atmospheres and in building materials, and these fibres lend themselves to monitoring by optical techniques, e.g. PCM and PLM. However, certain industrial applications and processes reduce the fibre bundles to individual fibres which require more sensitive TEM monitoring techniques. The XRD technique is used primarily as a supplement to PLM or TEM for quantitative analysis. Federal requirements for asbestos monitoring and compliance monitoring techniques were reviewed in this presentation. Requirements for laboratory accreditation and quality assurance programmes were also discussed.

### Automation of DNA profiling procedures in law enforcement forensic laboratories

*J. E. Johnson, J. MacMurray and W. G. Engelhart, Hamilton Company, 4970 Energy Way, Reno, NV 89502, USA*

In 1991, the FBI created the Combined DNA Index System (CODIS). This nationwide system allows forensic laboratories to store and match DNA records from convicted offenders and crime scene evidence. Forty-three states have enacted legislation requiring convicted offenders to submit samples for DNA databanking. DNA 'fingerprinting' by the Restriction Fragment Length Polymorphism (RFLP) technique is a labour-intensive process, often requiring weeks to produce results. The time-consuming sample preparation associated with RFLP and PCR analyses, and the massive number of samples involved makes implementation of DNA databanking a major challenge to forensic laboratories at the state and local level.

Automation of sample archiving and DNA processing procedures is the solution adopted by several state laboratories to manage their workloads without increasing their staff. Robotic pipetting systems are utilized to archive blood samples, extract and cleanup DNA, prepare DNA fragments for RFLP or PCR, and store isolated DNA. Automation protocols for DNA archiving and profiling were reviewed along with analytical results and laboratory productivity gains achieved.

### Quick screening for drugs of abuse by fast gas chromatography

*Cheryl Pape, Erin Wiley and Anthony Andrews, Centre for Intelligent Chemical Instrumentation, Clippinger Laboratories, Ohio University, Athens, OH 45701-2979, USA*

Forensic laboratories are in need of fast screening methods for biological samples when the presence of drugs of abuse is suspected. A viable method, which provides the necessary speed of analysis, is fast gas chromatography (GC). A fast GC technique incorporating a carbon dioxide-based cryotrap/thermodesorption inlet system enables instantaneous volatilization of analytes onto the head of the column, decreasing band broadening while increasing sensitivity.

The retention times of several drugs of abuse and some principle metabolites have been determined. Intra- and inter-day, as well as inter-week variances of retention times have been calculated. Intra-day relative standard deviations of retention time were 2% or less. Experimental conditions used to obtain these results, as well as variance data, were presented.

Reproducibility of peak heights and peak areas has been greatly affected by the cryotrap temperature. When the cryotrap temperature is excessively cold, the resistive heating mechanism is inefficient in completely volatilizing the analytes. Cold-trapping the analytes at a high temperature that is still below the boiling point of the compound somewhat solves this problem. Development of a constant temperature cooling system would essentially eliminate the problem. The effects of the cryotrap temperature on desorption of the drug components were discussed in detail.

Results of a separation of several drug components obtained using a dual column system were presented. Detection of the compounds has been accomplished with a flame ionization detector (FID). Limits of detection were provided, as well as a discussion of the insensitivity of the FID to the drugs under investigation. An alternative method of detection was also discussed.

### Automated chemical analysis system for LNG reference standard

*Yasushiro Gomi, Junichi Akiyama and Yasuhiro Kobayashi, Fundamental Technology Research Laboratory, Tokyo Gas Co., 16–25 Shibaura, 1-chome Minato-ku, Tokyo, 105, Japan*

Liquefied Natural Gas (LNG) is usually traded by total calorific value. This total calorific value is specified in the contract calculated at the unloading sites from the results of chemical analysis, which is performed in accordance with the gas chromatography described in GPA (Gas Processors Association) Standard 2261. Accordingly, the accurate chemical analysis at all ports where LNG is unloaded from tankers is important for calculating the total calorific value. The reference standard to be referred to in the analysis generally uses the mixed gas of known concentration resembling the composition of LNG. The content of each constituent in the reference standard gas is determined by the sub-atmospheric pressure gas entry method in accordance with GPA 2261. According to the sub-atmospheric pressure sample entry method, all kinds of pure gases, each corresponding to the components of natural gas or similar gaseous mixture, are prepared in advance. Each pure gas, at a pressure corresponding to the concentration of each component in the standard gas, is introduced to the GC. The peak area appearing in the chromatogram of a pure gas can be approximately the same as a component in the standard gas. Therefore, all the response factors are obtained by each pure gas, the composition of the reference standard gas can be correctly determined using these response factors. We have developed a fully automatic gas chromatograph (GC) system for determining the composition of the LNG reference standard by the sub-atmospheric pressure sample entry method.

This automated chemical analysis system comprises a sequence controller, personal computer, two precise pressure sensors, flow-switching cocks, two vacuum pumps and four GCs with thermal conductivity-type detector (TCD). These instruments can be controlled by a a sequence controller and personal computer. As a result, this apparatus enables a series of analytical steps in the sub-atmospheric pressure gas entry system to be performed automatically. With four TCD-GCs, 16 compositions of LNG or refinery gas can be analysed completely within 18 h.

### Trace levels of transition metals, silicate and borate in high purity waters with microbore on-line ion chromatography

*Rosanne Slingsby, Maria Rey, Alex Kirkland, Jacek Jagodzinski and John Riviello, Dionex Corporation, 1228 Titan Way, Sunnyvale CA 94086, USA*

Water quality in high purity waters (HPW) is commonly assessed on-line and/or by grab sampling using several parameters, including conductivity, total organic carbon, particulate count, anions, cations, transition metals, silicate and borate. Measurements of trace silicate and borate have proven useful as indicators for breakthrough of mixed bed ion exchangers used in HPW production. Detection limit requirements are lowered periodically to reflect the latest in the state-of-the-art needs in semiconductor, disk drive and manufacturing processes. In this paper, we discussed benchtop and on-line results in our current efforts to deliver sub-ppb detection limits, while improving ease-of-use for transition metals, silicate and borate methods. The transition metal and silicate methods utilize a 2 mm format for columns and post-column reactors in order to lower reagent consumption and improve ease-of-use. New column chemistries are also employed. The borate system uses a newly developed borate-specific concentrator column that is coupled to an ion exclusion separator.

### Automatic evaluation of $^1$H NMR spectra of combinatorial libraries

*Renate Bürgin Schaller, Pius Portmann and Ernö Pretsch, Department of Organic Chemistry, Swiss Federal Institute of Technology (ETH), CH-8092 Zürich, Switzerland and Reinhard Neudert and Bernd Follmeg, Chemical Concepts, P.O. Box 10 02 02, D-69442 Weinheim*

A new $^1$H NMR spectra-structure compatibility checking system has been developed with a view to automatically evaluate combinatorial libraries. The program presented here was written using the Proton Shift program for spectra estimation and is being interfaced to the SpecInfo database. First, a database of all the experimental spectra is generated automatically and the chemist enters the structures proposed by means of a drawing program of his choice. The evaluation module developed then uses the spectra and the corresponding structures. Chemical shifts are, on the one hand, estimated from the structures and, on the other, approximately derived from the experimental spectra by applying a heuristic procedure. Comparison of the resulting two sets of chemical shifts provides a measure for the quality of agreement. If necessary, a set of proposed structures can be compared with a single spectrum, or vice versa, and ranked accordingly.

### On-line FTIR monitoring of an industrial esterification process

*Rocko Burgo and James Vetter, Inolex Chemical Company, Jackson St., Philadelphia, PA 19148, USA; Steve M. Donahue, Scott W. Strand, Dave B. Hobart and Norman E. Van Order, Jr, ASI Applied Systems, 8223 Cloverleaf Dr., Millersville, MD 21108, USA*

Industrial chemical processes often need on-line measurement techniques that provide compositional analysis. In many cases, this information arises from off-line wet chemical analysis of the reaction mixture. While off-line methods provide valuable information, they require long analysis times, generate waste and expose operators to hazardous samples. The effect on the chemical process is long reactor hold times, low efficiency and slow response to process upsets. In contrast, on-line measurements provide real-time information to improve process efficiency, product quality and operator safety.

The use of *in situ* mid-infrared spectroscopy has emerged as a powerful approach for monitoring chemical reactions. It offers inherently fast, sensitive measurements that contain specific compositional information. Although these characteristics are suitable for tracking Hydroxyl Number (OH#) and Acid Number (AN), only near-infrared measurements have found wide acceptance at the plant level. The concerns over using FTIR relate to sampling technology, the accuracy and detection limit for these components, and whether a single method is applicable over a wide range of products.

Direct esterification of organic acids represents a common reaction used to produce synthetic base stocks and additives for the lubricant industry. Typically, the production requires off-line determination of the OH#

(AOCS) and AN (ASTM) to monitor and control the process. Recent advances in FTIR-based technology have allowed *in situ* monitoring of esterification reactions to investigate the feasibility of replacing the off-line methodology. This paper described the application of FTIR process monitoring in the ester-based lubricants industry. The topics for discussion included the correlation of infrared data to OH#, trend analysis of reagents (alcohol, acid) and product (ester), and calibration design for full reaction monitoring versus end point detection.

### On-line composition analysis in molten glass during waste vitrification

*Roger W. Jones, Stanley J. Bajic and John F. McClelland, Ames Laboratory, Iowa State University, Ames, IA 50011, USA; James C. Marra, Kathryn M. Marshall and John M. Pareisz, Westinghouse Savannah River Company, Savannah River Technology Center, Aiken, SC 29808, USA*

Vitrification is being developed by the Department of Energy as a method of immobilizing radioactive transuranic metals prior to their shipping and long-term storage. Real-time monitoring of the molten glass as it exits the vitrification system would be valuable for process control, quality assurance and nuclear-material accounting. It would also reduce worker exposure by reducing the need for physical sampling. We reported on developing thermal emission spectroscopy as an on-line, real-time analysis technology for this application. Many oxides of actinides and lanthanides have low-lying electronic transitions in the near-infrared and visible regions. The molten glass stream is sufficiently hot (> 1300°C) that transitions anywhere in the near infrared region should produce an observable emission.

A prototype near-infrared emission spectrometer has been built based on an AOTF crystal, and has been tested with a non-radioactive molten glass stream. The molten stream consisted of a glass frit containing neodymium oxide that had been developed for plutonium vitrification. The frit was spiked for the tests with various known amounts of ytterbium oxide, which is a spectroscopic surrogate for plutonium and americium.

### Continuous on-line GC monitoring with a microtrap

*Chaohua Feng, Chen Yun, Arthur Lai, Yong Xu and Somenath Mitra, Department of Chemical Engineering, Chemistry and Environmental Science, New Jersey Institute of Technology, Newark, NJ 07102, USA*

Continuous on-line monitoring of volatile organic compounds (VOCs) in air emission offers the advantage of obtaining real-time information about a chemical process or an environmental emission. Compared to traditional air sampling methods, where field sampling is followed by laboratory analysis, continuous monitoring application requires sampling, sample conditioning and analysis to be done on-line. No delay occurs between sampling and analysis, consequently results are obtained in real-time. Moreover, since sample handling and storage is eliminated, these techniques also produce more accurate results.

Sampling and injection devices based on microtrap absorption/desorption have shown advantages in continuous, on-line monitoring of VOCs in air. Microtrap is a stainless steel capillary packed with an absorbent. The microtrap serves as a preconcentrator and an injector, and is connected directly to a column during GC analysis. The absorbent in the microtrap can selectively retain and concentrate organics, while background gases are allowed to pass through. The injection is made by heating the microtrap with a pulse of electric current. The thermal energy can be supplied at a short pulse duration of 0.8–1.2 s. Each electric pulse generates an analyte 'concentration pulse' that serves as an injection for GC analysis. Continuous monitoring is achieved by passing the sample continuously through the microtrap and heating the microtrap at regular intervals. Each desorption pulse serves as an injection.

### Automation of determination of acid number or base number for petroleum products

*Suzanne Bode Blohm and Rick Dadson, Bohdan Automation, 1500 McCormick Boulevard, Mundelein, IL 60060, USA; Dennis W. Hughes and David L. Wooton, Ethyl Petroleum Additives, 500 Spring Street, Richmond, VA 23217, USA*

This paper described the unique combining of a workstation with a titrator, creating a fully automated, walkaway system to perform acid number and base number determinations of petroleum products utilizing ASTM methods D664 and D2896. Prior to the development of the workstation, the process required choosing between complex robotics systems or limited automation, e.g. sample changes coupled with tedious manual sample preparation.

The data presented are from a workstation which was developed for use in a centralized testing facility at Ethyl Corporation which receives field test-used oil samples of various viscosities and requiring multiple titrations. Samples are sorted by the required titration method using a bar code number and an operator-defined network file, eliminating the need for presorting samples as part of the instrument set up. To meet the ASTM-required weight specifications which range from 0.10 to 20 g, the workstation utilizes disposable pipettes, beakers and a syringe pump for sample handling. Sample weights are confirmed with a 4-place analytical balance and sent to a workstation-controlled titrator, which performs the desired titration. After the titration, the titration data are recorded to a file for future uploading to the LIMS. The necessary electrode cleaning and conditioning process is performed prior to accessing the next sample.

For ease of operation, a titration method, its associated solvents for sample dissolution, and parameters for electrode cleaning are automatically accessed throughout the run. This enables the operator to return to collect a finished report.

### On-line detection of accidental pollution in industrial wastewater

*H. El Khorassani, P. Trebuchon, H. Bitar and O. Thomas, Laboratoire Génie de l'Environnement Industriel Ecole des Mines d'Alés, 6, avenue de Clavières, 30319 Ales Cedex, France*

Industries have nowadays to check their wastewater, either for the control of wastewater treatment plant, or for the survey of their impact on receiving medium. The systems used are generally complex and expensive, and therefore more simple and cheap instruments must be developed.

The aim of the work was to propose a procedure for the detection of accidental pollution, using both ultraviolet spectrophotometry and global organic pollution measurement, if needed.

The general procedure starts with the study of the UV spectrum of wastewater for the detection of a quality variation with respect to usual effluent composition. A specific method, called UVDIAG, has been designed for the deterministic deconvolution of the spectrum and the diagnosis of quality variation. In parallel, the organic load is directly measured in order to complete the survey procedure. If the UVDIAG results show a quality variation, the level of organic pollution can lead to wastewater by-pass (for a postponed treatment). Obviously, the proposed procedure cannot be used for non-UV-absorbing compounds.

The procedure is carried out with the use of an on-line UV detector, placed on the inlet of the wastewater treatment plant, and a TOD (or TOC) meter. This last can be located either on-line near the UV detector or in the laboratory.

The interest of the procedure was illustrated from experimentation carried out in chemical and petrochemical plants. Several cases were explained, showing the relation between the characteristics and the presumed origin of accidental pollution, generally coming from incidents in process units.

### LIMS on the net? You bet!

*Donald Kolva, Aaron Kushner, Tom Miller and Christine Paszko, Accelerated Technology Laboratories, Belmont, CA 94002, USA*

Laboratory Information Management Systems (LIMS) are changing rapidly, and the World Wide Web is today's 'next generation' venue for disseminating real-time information. The widespread use of the internet, and its deployment to laboratories throughout the world, means that lab managers must learn how to take advantage of this technology as well as appreciate its limits.

New web-based applications mean that laboratory information management users will be able to access their data whenever and wherever there is web accessibility. Sample results, requisitions and automated e-mail, e.g. may be delivered through web-based applications. 'Push technology' may deliver high priority results or values that have exceeded test limits to a users desktop when they are available.

This communication revolution will present new challenges as well. Such issues as security, access, speed, cost and availability will affect the adoption of this technology. This presentation addressed the technical capabilities and challenges that lab managers face as they adopt this technology.

### Determination of uranium by FIAS-ICPMS

*Zeev Karpas and Avraham Lorber, Department of Analytical Chemistry, Nuclear Research Center, Negev, P.O. Box 9001, Beer-Sheva, Israel 84190; Ludwik Halicz and Itai Gavrieli, Department of Geochemistry, the Geological Survey of Israel, Jerusalem, Israel*

Ingestion of uranium compounds, which are commonly abundant in the biosphere, occurs through human diet, mainly drinking water, and also through inhalation. The uranium content that is excreted in urine may serve as an indication of the uranium body content. A method based on flow injection and inductively coupled plasma mass spectrometry (FI-ICPMS) was found to be most suitable for determination of uranium in clinical samples (urine and serum), environmental samples (sea water, wells and carbonate rocks) and in liquids consumed by humans (drinking water and commercial beverages).

The mean value for the concentration of uranium in urine was found to be 12 ng/l, leading to a diurnal excretion of about 20 ng/l. This is two orders of magnitude below the daily intake, indicating that only about 1% of the ingested uranium is taken up by the body. In Israel, the uranium content in drinking water reflects the source of the water: local wells or water from the Sea of Galilee. Uranium concentration in wells and drillings in arid areas may serve as a means of correlating these water sources with specific aquifers. Finally, the method can be used to screen carbonate rock samples, which are used for dating based on uranium/thorium ratio, prior to TIMS measurements.

### A client/server approach to laboratory automation

*Soheil Saadat, Vic Patil, Mitch Pearlman and Mark Mullins, Scientific Software, 6612 Owens Drive, Pleasanton, CA 94588, USA*

Managers of today's larger laboratories oversee many analysts working on multiple projects using hundreds of instruments from different manufacturers. Their task has become more challenging due to increasing regulatory requirements for data integrity, security and audit trail. Laboratory throughput and efficient use of instruments is becoming increasingly more important.

This paper describes recent advances in Windows Client/Server architecture eliminating the traditional boundaries around instruments and PCs. Instruments may now be monitored/controlled over the network or via the Internet. This allows for data sharing across multiple clients and installations.

In addition to data sharing and instrument control/monitoring, the Windows® 95/NT environments provide much-needed advances in security and data protection. EZChrom Elite Client/Server's unique enterprise architecture is used to demonstrate one implementation of a system designed for larger laboratories.

### How much lab can we put on-a-chip with capillary electrophoresis?

*D. Jed Harrison, Thompson Tang, C. Colyer, Charmaine X. Qiu, Nghia Chiem, Gregor Ocvirk, Shakuntala Mangru, Hossein, Salimi-Moosavi and Cameron Skinner, Department of Chemistry, University of Alberta, Edmonton, AB, Canada T6G2G2*

Micromatching technology has provided a means of fabricating miniaturized, three-dimensional structures capable of performing clinically relevant analyses. This technology offers promise in developing miniaturized instrumentation with a high level of automation and rapid analysis times. Many of the successful active devices, in which fluid flow and delivery is driven on-chip have been based on electrokinetic effects. In these devices, electroosmotic flow has been used to drive fluid flow in complex networks of fluid channels, providing pumping and valving action without the need for moving parts. On-chip chemical reactions, dilutions, separation steps, post-separation reactions, peak heart cutting steps, polymerase chain reactions, cell lysis and other laboratory procedures have been demonstrated. The possibilities of integrating these various procedures together on-chip is very exciting. However, barriers remain to be overcome, including the need for sensitive detection, simple sample introduction, inexpensive interfacing to the external environment, a better understanding of the fluid mechanics and demonstration of good performance when many elements of a system are integrated on a single chip. This paper surveyed the results in our laboratory directed towards electrokinetically controlled microfluidic analytical devices, and attempted to evaluate the present progress in the field in terms of the barriers discussed above.

### Simple graphical user interfaces (GUIs) for a sophisticated data system using Visual Basic

*Steve Miller, Vikram Patil, Dale O'Neill and Soheil Saadat, Scientific Software, 6612 Owens Drive, Pleasanton, CA 94588, USA*

Data Acquisition and Control Software has evolved into a very powerful tool for today's laboratories. Now, true 32-bit Data Acquisition and Reprocessing software is available for the Microsoft Windows NT 4.0 platform. Windows NT 4.0 brings powerful new tools to these types of application.

One such data system, EZChrom Elite, provides users with simple commands that allow full control of the program using a programming language, e.g. Visual Basic. Visual Basic makes it simple for 'non-programmers' to create custom Graphical User Interfaces (GUIs) that allow full control of data acquisition, while limiting the required amount of user interaction.

One such interface is designed for high production laboratories. Managers are typically in charge of method parameters, e.g. integration, calibration fits, reports, etc. Technicians simply run instruments by making single injections. This GUI is very simple to use. The technician chooses an instrument from a scroll box, selects a method, types in a sample name and clicks the 'Ready to Inject' button. This information is seamlessly passed to the Server, the instrument readied with the specified method and file name, and starts running when the injection is made.

Another GUI takes advantage of Windoes NT Network security by adding it to EZChrom Elite Client/Server User access levels. These GUIs and the steps used to create them were discussed in this presentation.

### Ultimate control: accessing and controlling instrumentation behind the network firewall

*Michael Johnson, Deborah Sweetin and Roger Crandell, Los Alamos National Laboratory, CST-8, PO Box 1663, MS G740, Los Alamos, NM 87545,USA*

Remote access is the ability to access data or applications stored on a PC or corporate network from another PC over some form of communication link. Remote access technology is a valuable capability which provides increased productivity in today's modern laboratory environment. Through advanced technology, it has become possible to access information, and establish control of laboratory instrumentation and computers from a remote location. As a result, a scientist can maintain full operational control of the actual instrumentation either from a distance of several feet or any number of miles.

This presentation focused on the increased productivity resulting from developing remote access capability to laboratory instrumentation and computers. A unique challenge to remote access technology is the accessing of instrumentation behind a network firewall designed to limit access. However, network access through smartcard technology, current software and modern operating sytems makes this process possible.

Although configuring the connections can be a time-consuming and tedious undertaking, one does not have to be either an expert or a computer programmer to 'get connected'. The actual procedures used for configurating the necessary hardware, system software and remote access software to 'remotely access' a laboratory ion chromatograph located behind a network firewall were discussed, along with the increased productivity resulting from greater than 50% increase in sample throughput.

### Bar code sample labelling and tracking alternatives for remote labelling applications and two-dimensional bar code labelling for information on small label applications

*Clarence Gilles, Innovative Management Services, 833 Wind Bluff Point, Springboro, OH 45066, USA*

This study is on bar code labelling and the tracking of laboratory samples collected in remote environments. A remote environment is defined as any location that is not in the laboratory itself, e.g. landfills, crime scenes, animal habitats, etc.

While various bar code tracking processes are currently practiced, one method minimizes the possibility of human error. Through the use of new products, end-users can now generate bar code labels at remote sites, providing accurate data for both the technicians and the laboratories. Today's durable label materials assure data integrity and longevity throughout the process.

(A) Benefits of bar coding over hand-written sample labelling and tracking systems:

- Afford keyless entry which reduces input and transposition errors.
- Reduce data input time in the field and laboratory.
- Diminish log-in time as a complete chain of custody is created.
- Provide accurate identification and location of samples through the entire laboratory process.
- Decrease critical laboratory turn-around time of samples.

(B) Bar code label and printing product capabilities:

- Make integration with custom programs which meet laboratory and technician requirements possible.
- Allow choices for linear or two-dimensional bar code.
- Permit scanning and data collection alternatives.
- Provide reporting options for billing, regulatory requirements and sample logging.
- Identify the sample with automatic date and time, technician and sample number.
- Afford the ability to add additional information through the keyboard, e.g. location and case number.
- Establish the chain of custody at the sample location.
- Are designed to print on small adhesive labels which apply to multiple surfaces and conditions.
- Offer data transfer capabilities to LIMS systems or laboratory host systems through various communication alternatives.

### Approaches to automation of compound dissolution in drug discovery

*Suzanne Bode Blohm and Sally Dowling, Bohdan Automation, 1500 McCormick Boulevard, Mundelein, IL 60060, USA*

Typically, drug discovery includes three steps: compound synthesis, compound dissolution, and structural characterization and activity analysis. The workstations described in this paper were developed to automate the compound dissolution step and the sample aliquoting for analysis step of the drug discovery process. Two examples presented herein demonstrate various options in automation, which enable the operator to continue using existing equipment and labware.

Compound dissolution can be carried out with a fixed volume or by calculating a volume based upon a desired concentration. The molecular weights used for calculating the dissolution volume are contained in an operator-selectable file. In one example, the sample weights are obtained from a weighing workstation, which allows the operator to use the same sample racks for both the weighing workstation and dissolution workstation. In the second example, the workstation includes a 4-place analytical balance to perform the weighing process. The racks in this case were designed to be used for the compound dissolution, as well as sample collection racks in the synthesis step and the centrifugal evaporation step. Both cases eliminate potential errors in transferring tubes from one instrument rack to another. Sample transfer and dilution can be into either microtiter plates for bioassay screens or septum vials for HPLC or MS analysis.

Software features, e.g. number of wash cycles and volume of wash solvent give the operator control in eliminating sample carry over.

### Sound for pharmaceutical process monitoring?

*R. M. Belchamber and M. P. Collins, Process Analysis and Automation, Fernhill Road, Farnborough, Hampshire GU14 9RX, UK; M. Whitaker and D. Rudd, Glaxo Wellcome, Ware, Hertfordshire SG12 0DP, UK*

Sound provides an added dimension to process measurements.

In a scientific world dominated by the electromagnetic spectrum, sound has largely been ignored as an analytical probe. Many processes have quite distinct acoustic emission characteristics. Most processes that involve multiphase systems produce some kind of measurable activity. For instance, crystallization and effervescence usually have some measurable acoustic activity.

Acoustic emission is rich in real time information. With suitable signal processing, acoustic emission can potentially be integrated into closed loop control. Measurements are made via non-invasive transducers. This enables installation costs to be kept low and eliminates contamination problems. High frequency (ultrasonic) transducers effectively localize the source of the acoustic emission activity and eliminate interference from extraneous sounds.

We have been focusing on heterogeneous processes associated with pharmaceutical production. These include fluid bed drying and granulation, mixing, blending and disintegration. Particle size, velocity, shape and hardness all effect the acoustic emission characteristics.

The goal of this work was to develop robust methodology enabling these processes to be controlled better, end points accurately determined and upsets avoided.

### Implementing laboratory method development and data processing software in process gas chromatographs

*Christoph Klawun, Steve Trimble and Brad Diehl, Applied Automation, P.O. Box 9999, Bartlesville, OK 74005, USA; Soheil Saadat and Mitch Pearlman, Scientific Software, 6612 Owens Drive, Pleasanton, CA 94588, USA*

In modern analytical laboratories, proprietary chromatographic method development and data processing workstations have been largely replaced by software packages that run on standard PC platforms, e.g. Windows 95 and NT. User interface design guidelines for these platforms flatten the software learning curve. Furthermore, data exchange with other applications, e.g. word processors or spreadsheets can easily be accomplished via standard file formats or other means of Windows-specific mechanisms (clipboard, DDE, OLE).

However, for process gas chromatographs, method development, setup and troubleshooting is still carried out through proprietary software, databases and networks. This paper presented a completely redesigned generation of process gas chromatography analysers that are networked using the standard interface protocol TCP/IP over ethernet and other wiring options. The operation of the analyser is driven by a real-time database accessible via SQL and custom tools.

For method development and chromatogram analysis, the standard chromatography software package EZ-Chrom Elite has been extended to interface with the process gas chromatograph for method setup. In addition, EZChrom Elite's core algorithms for chromatogram integration, quantitation and calibration have also been embedded in the analyser's firmware. This ensures matching results both on the Windows 95/NT and embedded platforms. Overall, a significant reduction in cost-of-ownership is expected through increased productivity and non-proprietary interconnectivity.

### Real-time analysis of tropospheric aerosols

*Kimberly A. Prather, Christopher A. Noble, Eric E. Gard and Deborah S. Gross, University of California, Riverside, CA 92521, USA*

Over the past five years, our research group has been involved in the development of a new technique for aerosol analysis, Aerosol-Time-of-Flight Mass Spectrometry (ATOFMS). ATOFMS provides real-time data on the size and chemical composition of individual aerosol particles. In order to obtain this information, the techniques of aerodynamic particle sizing and time-of-flight mass spectrometry are combined in a single instrument.

ATOFMS is being used to directly analyse ambient aerosol particles with the goal of establishing unique correlations between individual particle size and chemical composition. The recent development of two field-transportable ATOFMS instruments allowed us to participate in field studies in Southern California. In these studies, three ATOFMS instruments were strategically positioned along a path that allows real-time monitoring of transformation of aerosols in a common air parcel as they move through the three locations. We are using these data to establish links between the presence of particular types of particles with such factors as the time of day, weather conditions, and measured concentration levels of gaseous smog components including VOCs, $NO_x$, $SO_x$ and $O_3$. These studies provided unique data on tropospheric gas-particle processes which can be used to develop appropriate strategies for controlling air pollution. This presentation described the results of these recent atmospheric field studies.

### Real-time microparticle analysis using tandem mass spectrometry

*W. B. Whitten, P. T. Reilly, R. A. Gieray and J. M. Ramsey, Oak Ridge National Laboratory, P.O. Box 2008, Oak Ridge, TN 37831-6142, USA*

Rapid real-time chemical characterization of individual particles represents a near ideal approach to identifying understanding distributions of microparticulate in various matrices, e.g. the atmosphere, work place or in liquid streams. Traditionally, such analyses have been performed on collected particles using various analytical tools, e.g. laser desorption mass spectrometry, secondary ion mass spectrometer and photoelectron spectroscopy. These approaches are, in general, laborious and potentially compromise the integrity of the samples due to long delays between collection and analysis. Over the last several years, a number of research groups have independently developed methods for analysis of individual microparticles based upon laser desorption/ablation coupled to mass spectrometry. All of these groups have developed atmospheric sampling interfaces which allow particles to be directly sampled from atmospheric pressure sources into a vacuum chamber. The associated gas expansion accelerates particles to a terminal velocity that is dependent upon the particles size, down to some asymptotic limit. Measurement of particles velocity provide size information on the particles. The gas expansion process also allows the particles to be collimated and delivered to an ionization region where typically, UV laser radiation is used for desorption/ablation ionization. Our group has been unique in the use of an electrodynamic ion trap for mass analysis of the laser-generated ions. The ion trap allows efficient collection of these ions and importantly ion manipulations for tandem mass spectrometry. Multiple stages of mass spectrometry are critical for obtaining structural information for molecular species contained in microparticles. Applications discussed included analysis of airborne bacteria, soot, actinide-containing particles, as well as water-borne microparticulates.

### Monitoring the bioremediation of explosives using liquid chromatography/mass spectrometry

*B. J. Spencer[1], S. Thiboutot[2], G. Ampleman[2] and J. Hawari[1], [1]Biotechnology Research Institute, National Research Council Canada, 6100 Royalmount, Montreal, QC, H4P 2R2 Canada; [2]Defence Research Establishment Valcartier, P.B. 8800, Courcelette, QC, G0A 1R0 Canada*

The remediation of soil and groundwater contaminated with explosives from military test ranges and munitions plants has been studied for several years now. A common method for remediation is using microorganisms to destroy the contaminants in soil and groundwater. Bioremediation has proven to be an efficient and cost-effective technique for cleaning up environmentally problematic sites. Advances in the development of technologies in this area rely on a fundamental understanding of the metabolic pathways by which microorganisms transform organic pollutants.

Monitoring the bioremediation process is critical for technology assessment and determination of the toxicity of the output discharged into the environment. Identification and quantification of intermediates and end-products have traditionally been carried out using GC/MS and HPLC. Recent advances in LC/MS, however, have made this technique suitable for the determination of metabolic pathways.

This presentation described the use of electrospray (ESI) and atmospheric pressure chemical ionization (APCI) techniques for LC/MS to monitor the fate of explosives in remediation processes. Time profiles for the disappearance of the energetic compound, e.g. TNT, RDX and HMX, and the formation of their metabolites and end-products will be used to construct the metabolic pathways of the biodegradation of these compounds.

### Automated solid-phase extraction method development for use with biological fluids

*David T. Rossi, Bioanalytical Core Group, Department of Pharmacokinetics, Dynamics and Metabolism, Parke-Davis Pharmaceutical Research, Division of Warner-Lambert, Ann Arbor, MI 48105, USA*

With the widespread acceptance of LC/MS/MS as a predominant technique in Bioanalytical Chemistry, there has been a resurgence of interest in the automation of solid-phase extraction for sample preparation. This resurgence has been fuelled by the relative ease with which the technique lends itself to automation. One approach which has lagged behind has been the automation of the method development step. Several approaches to automated solid-phase extraction method development have now been evaluated and compared.

An automated solid-phase extraction method development system utilizing a Zymate XP robot and a custom-designed 144-port manifold has been developed and extensively evaluated. Quantitative recoveries ($96 \pm 6\%$) of example drug substances from blood plasma were obtained with imprecision of 2–10% RSD. One important advantage of this system is the ability to completely automate the method development experiment, including the production of spiked biological fluid samples, selection of solid-phase sorbents, and selection of wash and elution solvents.

A second approach utilized an automated workstation to develop, characterize and validate two separate HPLC methods for quantifying drugs in plasma. Method development was facilitated by workstation functions which allowed wash solvents of varying organic composition to be mixed and tested automatically. Imprecision estimates were within 6.0 and 2.0% RSD across the respective calibration ranges, and inaccuracies were between $-1.2$ and $+4.8\%$ RE over ng/ml and µg/ml calibration ranges, respectively. Optimized recoveries were quantitative and were generally $> 90\%$ for the four analytes tested, and depended heavily, as expected, on the composition of the wash solvent.

A third approach considers the 96-well workstations, and their utility in automation of solid-phase extraction method development. Sample throughput benchmarks for each of these approaches, as well as their advantages and disadvantages were discussed.

### On-line TLC-SWASV for the real-time determination of $Cd^{2+}$, $Cu^{2+}$ and $Pb^{2+}$

*Steven C. Petrovic and Howard D. Dewald, Clippinger Laboratories, Department of Chemistry, Ohio University, Athens, OH 45701, USA*

Thin-layer chromatography (TLC) still remains the simplest and most accessible form of chromatography to date, and is used to separate a variety of chemical species, including metal cations. Quantitation of metallic cations is typically accomplished by post-chromatographic derivation followed by either visually determining the TLC spot size or densitometry. Reports of quantitative TLC using electrochemical detection are few, and only a subset of these involve analyte quantitation in real time, unlike other chromatographic methods.

We are using square-wave anodic stripping voltammetry (SWASV) as a detection method for metal cation ($Cd^{2+}$, $Cu^{2+}$, $Pb^{2+}$) during a TLC separation, and refer to this method as On-Line TLC-SWASV, indicating our ability to acquire data in real time. A screen-printed electrode consisting of a carbon microelectrode array, a Ag/AgCl reference trip and a carbon auxiliary electrode is secured against a carboxymethyl cellulose TLC plate in a custom-made TLC development chamber. During chromatographic development, a 30 s deposition time is used to preconcentrate the analytes at the electrode surface prior to voltammetric detection, allowing a voltammogram to be obtained every 60 s.

SWASV is a sensitive detection method providing additional selectivity to quantitative TLC. Characterization of the analyte can easily be accomplished using voltammetric peak potentials in addition to $R_f$ values. Detection limits for all three analytes are currently less than 10 ng, which is more than an order of magnitude less than visually obtained detection limits.

### Optimization of dissolution methods using automated on-line HPLC analyses

*Michael E. Swartz, Patricia M. Young and Diane Pransky, Waters Corporation, 34 Maple Street, Dept. TG, Milford, MA 01757, USA*

Dissolution assays of solid dosage forms are performed during formulation development and as a condition of product release. These assays are currently performed predominantly by UV spectroscopic methods, however, trends toward increasingly complex formulations are generating the need for more sophisticated analyses by HPLC. HPLC analysis of dissolution samples can be performed either off-line or in an automated on-line fashion, depending upon method logistics. Automated on-line analyses must use optimized high throughput methods, be capable of handling complex dissolution media, including surfactants, and be capable of media replacement. In addition, the HPLC should also be capable of addressing sample neutralization and pooled sample analysis according to recently issued changes to

USP Chapter 711 on dissolution. Custom calculations are also required to generate rate release profiles used to determine if a dissolution test passes specifications.

In this presentation, we presented data illustrating the use of an automated on-line HPLC dissolution system capable of dissolution bath sampling, HPLC analysis, calculations and reporting that addresses the issues outlined above. The results of a comparison study of manual versus automated methods for traditional and pooled sample analysis that show method equivalency were also reported, along with precision, accuracy and recovery data.

### Multiplexers of multiple instruments: economics versus technology in NIR

*Haor Moise, LT Industries, 6110 Executive Blvd #800, Rockville, MD 20852, USA*

Near-infrared technology (NIR) has rapidly evolved from a lab analysis of one property in a sample to multiple properties on-line multi-point process installations.

The option of multiplexing has presented two alternatives: stream multiplexing or preferably the use of an optical multiplexer to reduce the risk of cross-contamination and the time required to flush between streams. As the number of measurement points increases, the cost of one analyser divided by the number of points being measured yields a smaller $/measurement ratio.

This paper presented and analysed the next technological breakpoint: full-fledge NIR analysers that are cost-effective to be ubiquitous at every measuring point. An instrumentation proposal that is distributed, asynchronous and ubiquitous delivers results in a robust, reliable and faster way, achieving and improving the economical cost of implementing it.

Case results were described and guidelines for evaluating the technological tradeoffs were presented.

### PBMS: facilitating the use, approval and acceptance of new measurement methods for environmental monitoring

*Llewellyn R. Williams, U.S. Environmental Protection Agency (EPA), National Exposure Research Laboratory, P.O. 93478, Las Vegas, NV 89193-3478, USA*

The Performance-Based Measurement System (PBMS) is a set of processes wherein the data quality needs, mandates or limitations of a programme or project are specified, and serve as criteria for selecting appropriate methods to meet those needs in a cost-effective manner. The EPA issued a notice of intent in the Federal Register (FR 62:193, pp. 52 098–52 100, 6 October 1997) to implement, to the extent feasible, a PBMS for environmental monitoring in all of its media programmes. Where PBMS is implemented, the regulated community would be able to select any appropriate analytical test method for use in complying with EPA's regulations. It is the EPA's intent that implementation of PBMS has the overall effect of improving data quality and encouraging advancement of analytical technologies.

Under PBMS, the Agency would identify relevant performance characteristics of analytical methods and specify quantitative performance criteria for each of those characteristics without prescribing specific procedures, techniques or instrumentation. The EPA intends to implement PBMS on a programme-specific basis; each programme has or is developing an implementation plan. Individual EPA programmes may need to adopt a phased approach, however, EPA's ultimate goal is to specify performance criteria that are not linked to methods, techniques or instruments.

In a programme where PBMS is implemented, the regulated community would be required to: (1) demonstrate that the measurement method to be used meets the specified performance criteria by documenting both initial and continuing performance according to a required protocol; and (2) maintain written certification that they used appropriate quality assurance procedures. PBMS is expected to apply to most physical, chemical and biological measurements conducted to the field or laboratory. Situations in which the PBMS would not apply were also discussed in this presentation.

Implementation of PBMS by the Agency represents a real opportunity for new technology developers: first, to be able to use published performance criteria as targets in their development efforts, and second, to have their worthy methods/techniques adopted without the long, cumbersome processes they have had to endure in the past.

The information in this abstract was developed for an oral presentation; it has not been peer-reviewed by the Agency.

### The determination of the holding time of mercury in tissue

*Debra Thompson, Elizabeth Fumal and Edward Schorr Jr, Environmental Research Institute, University of Connecticut, U-210, Storrs, CT 06269, USA*

The impact of mercury on the environment can be quantified by determining the mercury concentration in tissue. Mercury has a severe impact on the environment in trace amounts due to bioaccumulation. The trace level results must be obtained using the most accurate data generated from representative tissue samples. Any variation in concentration causes a large impact on results due to the low level range of the analysis.

Metals concentrations are believed to decrease in environmental samples stored over time, even when frozen. The instability of mercury makes it especially susceptible to this problem. The holding time for mercury in frozen fish tissue is specified in the EPA 'Guidance for assessing chemical contaminant data for use in fish advisories' as 28 days. This holding time has only been established for water.

A time-study digesting and analysing a deer liver and heart over a period of time was discussed. An accurate holding time for mercury in tissue was established. EPA

Method 245.6 was used to determine the total (inorganic and organic) mercury in tissues by cold vapour atomic absorption spectrometry.

### Development of an automated workstation to perform process development for new compound discovery

*James R. Harness, Bohdan Automation, 1500 McCormick Boulevard, Mundelein, IL 60060-4447, USA; Henry J. O'Connell, Gilson, 3000 W. Beltline Highway P.O. Box 620027, Middleton, WI 53562-0027, USA*

The pharmaceutical, biotechnology, chemical and agricultural industries continue to allocate more R&D resources into combinatorial chemistry and high throughput screening. The impact of these accelerated compound development activities is rapidly becoming a substantial increase in the number of compounds being handled by the Process Development groups. Automating the activities associated with this increased demand is viewed as critical for keeping up with these needs.

From an automation viewpoint, early stage process development activities can be broken down into functional categories that include small volume process screening/optimizing and larger volume process characterizing. The first equipment developed specifically for automating process screening/optimizing activities was described in this presentation.

The Automated Workstation that has been developed provides an automated mechanism for evaluating a wide variety of organic synthesis reaction conditions simultaneously. The Workstation consists of a multi-position, solution-phase organic synthesizer working in conjunction with a liquid handling robot and HPLC analyser. The organic synthesizer includes a reaction block that can perform 12 solution-phase reactions simultaneously. The reactions can be performed at 12 different temperatures ranging from $-20°$ to $140°C$ under inert conditions in volumes up to approximately 20 ml. Reactions are independently stirred with magnetic stir bars and can be refluxed to support the performance of reactions at or above the solvent boiling point. Reactants and reagents for setting up the reactions are held in septum-capped vials, and can be heated or cooled between $-10°C$ and $50°C$ with individual stir bar mixing. Reactants are automatically transferred to the reaction vessel by specially designed pipettors. Each reaction can be sampled at operator-selectable times, and the samples, then, passed to a liquid handling robot for preparation prior to HPLC analysis. The liquid handler can quench the reaction mixtures and perform diluting, mixing or filtering activities, as required, prior to injecting the samples into the HPLC analyser. HPLC analysis is performed on a fully functional gradient HPLC with diode array detection. The entire Workstation assembly is computer controlled, and all data from each instrument are compiled into a single report at the end of each run. Individual analysis and reaction software packages are provided for controlling each function separately.

### New generation chromatography software with advanced automated reporting capabilities

*Linda Jackson, James Schibler, Der-Min Fan and Damon Gragg, Dionex Corporation, Sunnyvale, CA 94086, USA*

Automation and chromatography data systems have helped analysts improve productivity by generating and reporting on more analytical results in less time. Today's chromatographers demand flexibility in defining the context and format of the reports generated from their results. Most data systems provide some formatting options for single-injection reports, but the options are often too limited or too difficult to use. Furthermore, few systems can generate format summary reports, which are often the most useful way to report results. These issues are addressed with a new object-based Report Designer which allows chromatographers to define, position and format the contents of the report (tables, header fields, graphics) in a manner similar to graphic design software. The Report Designer handles elements as objects that users can manipulate and combine as desired. The Report Designer provides a simple drag-and-drop user interface with unlimited report templates for all report classes: Calibration Update, Check Standard, Sample and Summary.

The Object Linking and Embedding (OLE) standard is supported, allowing dynamic objects e.g. spreadsheets and charts to be pasted in from other applications.

### Routine analysis using on-line identification methods in quality assurance

*H. Amine, S. Labreche, T. T. Tan and F. Loubet, Alpha MOS S.A, 20, Avenue Didier Daurat, 31400 Toulouse, France*

Routine use of electronic nose systems in quality control laboratories has driven the vendors to provide reliable and easy to use prediction tools. These identification/classification models are proprietary tools based on many well-known pattern recognition techniques, which include neural-networks, fuzzy-logic, conventional statistics and an Alpha MOS proprietary identification/classification algorithm. Different performances are required from the classifiers to provide qualitative information for varying requirements for the many applications in the routine quality assurance laboratory. Some of the requirements include:

(1) classifying typical/marginal/unacceptable;
(2) discriminating between two or more products;
(3) identifying a product as one of many products.

The key specifications for these prediction tools include reliability and accuracy in prediction, ease of use and robustness with long term stability. A further consideration would be the speed of prediction in the on-line/in-line applications.

With the use of a common database of different coffees and different pure chemicals using a Fox4000 system consisting of 18 sensors over a six month period, the different specifications were investigated using conventional statistics, neural-networks, fuzzy logic, statistics and an Alpha MOS proprietary identification/classification algorithm. Our method is composed of the following processes:

(1) removal of outliers;
(2) correction of sensors drift by calibration;
(3) summarizing of the most information contained in the raw data by a small number of synthetic variables;
(4) constructing of robust identification model.

Each identification model is associated relatively to geometrical, probabilistic or fuzzy rule. The most robust method is chosen to predict the on-line/in-line applications. The choice is made by comparing the performance of each model on the database generated in the learning step.

### On-line measurement of ozone precursors from $C_2$ to $C_{10}$ in ppb levels

*Felix Behm, Airmotec ag, Länggstr. 19, 8308 Illnau, Switzerland*

Ozone precursors are the main focus when monitoring air pollution. Today, equipment for monitoring these high volatile organic carbons (VOC) in the range from $C_2$ to $C_{10}$ are complicated, slow, bulky and consume up to several kW energy. For monitoring $C_2$ in ambient air, liquid nitrogen is used for cryotrapping normally. There is no way to measure the ozone precursors on site with this classical equipment. These limits are overcome with the automated and fast high resolution micro GC airmoVOC 2010. With this device, ppb level analysis of VOC from $C_2$ to $C_{10}$ within 30 min is possible. Because of its small size (19″, 3 units high), low power consumption and the use of compressed $CO_2$ for the cryogenic focusing, the airmoVOC 2010 is well-suited for monitoring VOC in unmanned stations, trucks, cars and even aeroplanes.

The principle allows for continuous mode operation (sampling during analysing time), so even high dynamic changes of the VOC will be measured correctly. The linearity and reproducibility over concentration ranges from 0.1 to 150 ppb in ambient air are better compared to laboratory equipment. The device is highly automated so the whole process can be run either stand-alone or under remote control by modem.

*Principle of measurement*

Airborne VOC are sucked through an adsorbent (stacked pack of CARBOTRAP®B/CARBOSIEVE® IIIS) which are cooled to about 18°C by expanding $CO_2$. At this temperature, $C_2$ compounds are safely trapped in up to 500 ml sample volume, but there is still little risk of condensation of humidity. To allow for continuous mode monitoring, several adsorbent tubes are placed on a drum. While the sample is taken on a tube, the preceding tube is desorbed into the chromatographic path. Only while rotating the tubes, the sampling is disabled. A sampling coverage by time of 98% can be achieved. The sample is thermally desorbed and focused on a micropacked cryotrap at low temperature (down to −78° C with $CO_2$). The sample is injected with a fast heating pulse up to 350°C in 20 ms on a 25 m column (0.2 mm i.d., 2 µm film with 2.5% phenyl). Chromatography is done with hydrogen as carrier gas under temperature and pressure programme. The signals from the micro FID with electronic controlled gas feed are integrated into the GC system. Both data (chromatogram and peak data) are sent via a RS 232 interface to a PC.

*Results*

The compounds range from ethane up to *n*-decane in concentrations around 30 µg/m$^3$. In about 10 min the compounds are separated with high resolution. The reproducibility at this concentration level is better 5% ($n = 12$). The linearity over the concentration range of 1–300 µg/m$^3$ (aromatic compounds) is better than 5%.

### A method for the analysis of total reduced sulphur (TRS) compounds in aqueous kraft mill process streams

*Brian O'Connor and Serge Genest, Pulp and Paper Research Institute of Canada, 570 St. John's Boulevard, Pointe Claire, Quebec, Canada H9R 3J9*

To date, a suitable published method for the quantification of the total reduced sulphur (TRS) components in aqueous samples, e.g. foul condensates, weak black liquors and effluents, from pulp and paper mills has not been demonstrated. While methods, e.g. purge and trap, headspace analysis and direct injection in conjunction with gas chromatography (GC) have been proposed, there are various drawbacks, and the methods have not been routinely used for the analysis of aqueous process streams for the pulp and paper industry.

In this work, a procedure for the analysis of TRS compounds in aqueous kraft mill process streams was further developed and optimized from a preliminary method outlined by the National Council of the Paper Industry for Air and Stream Improvement (NCASI). The method involves the use of a specially designed glass sparger for nitrogen purging of the aqueous sample followed by collection of the gas in a tedlar bag and analysis by gas chromatography. The method provides excellent recoveries (93–110%), and for spiked water samples and kraft mill process streams (condensates, combined mill effluents and weak black liquor), the average standard deviations were typically found to be $<4\%$. With the apparatus used in this study, the potential oxidation of methyl mercaptan to dimethyl disulphide did not appear to be a problem.

### Real time monitoring of ethylene oxide sterilization processes

*Terry R. Todd and Gary Brown, UOP Guided Wave, 5190 Golden Foothill Parkway, El Doraldo Hills, CA 95762, USA*

The utility of monitoring ethylene oxide sterilization processes using Near Infrared (NIR) spectrophotometers will be discussed. Ethylene oxide (EO) is the most common sterilizing gas now in use in the pharmaceutical packaging industry. There is a need for accurate measurement of the EO concentration in the isolation chamber on a real time basis. As in the hydrogen peroxide sterilization process, NIR can provide accurate and useful real time EO measurements as well. The process requirements will be defined leading to the instrumental requirements and spectral details of measuring ethylene oxide in the NIR region. These details will include detection wavelengths, minimum detection levels, accuracy, time response and potential interfering species.

### An on-line, *in situ* ethylene oxide gas analyser for sterilization

*Seetha Ananth and Gary Lang, Rosemount Analytical, 1201 N. Main Street, Orrville, OH 44667, USA*

Pharmaceutical manufacturers are in need of a real-time, reliable, safe, direct method to determine ethylene oxide (EtO) and moisture concentrations in sterilizers. Manufacturers must comply with established government regulations for validation and routine control of the EtO sterilization cycle.

Parametric release requirements mandate the direct measurement of both EtO and relative humidity in the sterilizer during a cycle. Gas chromatography, theoretical pressure differential techniques and relative humidity (RH) sensors are currently used to determine EtO and moisture levels during the sterilization process. However, these techniques do not allow for continuous monitoring.

The Rosemount AOTF-NIR$^{TM}$ Ethylene Oxide Analyser system provides *in situ*, real-time, direct determination of EtO and moisture throughout the entire sterilization cycle. The system is intrinsically safe and the measurements correlate with NIST traceable standards. Calibration techniques and results from actual sterilization cycles were discussed. Data were shown that illustrate that the analyser meets the AAMI standards for accuracy.

### Diesel process control using near-infrared analysis

*Martha Ranc, Larry McDermott and Ernie Baughman, Orbital Sciences 2771 N. Garey Ave., Pomona, CA 91757, USA; Peter Eversley, Lyondell-Citgo Refining Corporation*

Near-Infrared (NIR) analysis has recently received a lot of attention for the control of gasoline blending processes. Diesel fuel (fuel oil) is the second most common fuel produced by North American refiners, and actually is the prevalent fuel used in many other countries. Typical diesel production processes use fewer base components than gasoline blending. A multiplexed NIR analyser can be used to measure the finished diesel product and all of the base components simultaneously.

Diesel fuel is analysed for numerous chemical, physical and performance parameters using ASTM (American Society for Testing Materials) methods. These methods are typically performed in the laboratory and may take up to several hours to perform. Many of the key diesel fuel properties routinely measured can be measured using Near-Infrared Spectroscopy. On-line process monitoring allows refiners to dramatically reduce laboratory analysis turn-around times for several tests. This real-time analysis can further result in deceased production problems, resulting in higher product consistency.

### Aromatics and oxygenates monitoring by GC-IR analysis in the refinery

*Sheldon S. Cantor and Jay R. Powell, Bio-Rad Laboratories, Digilab Division, 237 Putnam Avenue, Cambridge, MA 02139, USA*

Pharmaceutical manufacturers are in need of a real-time, reliable, safe, direct method to determine ethylene oxide (EtO) and moisture concentrations in sterilizers. Manufacturers must comply with established government regulations for validation and routine control of the EtO sterilization cycle.

Parametric release requirements mandate the direct measurement of both EtO and relative humidity in the sterilizer during a cycle. Gas chromatography, theoretical pressure differential techniques do not allow for continuous monitoring.

The Rosemount AOTF-NIR$^{TM}$ Ethylene Oxide Analyser system provides *in situ*, real-time, direct determination of EtO and moisture throughout the entire sterilization cycle. The system is intrinsically safe and the measurements correlate with NIST traceable standards. Calibration techniques and results from actual sterilization cycle were discussed. Data were shown that illustrate that the analyser meets the AMI standards for accuracy.

### On-line low-level sulphur determination in refinery samples using ultraviolet fluorescence detection

*Stephen Tarkanic, John F. Welsh Jr and James S. Wreyford, Antek Industrial Instruments, 300 Bammel Westfield Road, Houston, TX 77090, USA*

Sulphur determination is one of the strongest challenges in the analyses of petroleum refinery and other industrial streams. This is particular true, as the future of analytical instrumentation will focus increasingly on taking the instrument to the sample, i.e. on-line monitoring.

One of the best available technologies today to meet this challenge is sample combustion followed by the detection of ultraviolet (UV) light from the fluorescence of sulphur dioxide. This technology has been used very successfully in such applications as gasoline and diesel blending, and natural gas processing. In these applications, the sulphur levels are generally as high as 20 ppm or higher. The detection of UV fluorescence has not been generally regarded as an effective technique on process streams that may have sulphur concentrations at the low ppm or sub-ppm level.

In this paper, we presented Antek Industrial Instruments' improved Process Sulfur Analyzer that successfully addresses this problem. Good sensitivity for low level sulphur using UV fluorescence was shown. Our data also demonstrated excellent short-term reproducibility and precision, as well as long-term reproducibility that is required for on-line applications.

### A new generation of process analytical liquid chromatograph: staying on the forefront of analytical needs

*Michael J. Doyle, Mary Jo Wojtusik, Edward Kaiser, Jill Jeko and Mindy Donofrio, Dionex Corp, 1228 Titan Way, Sunnyvale CA, 94088, USA*

On-line LCs have been used for many years in the pharmaceutical, chemical, power generation and semiconductor manufacturing industries. The unique capabilities of HPLC and Ion Chromatography to characterize a variety of analytes in demanding matrices have brought on-line LCs from a novelty to integral part of many industrial process monitoring programmes. The demands placed the use of these instruments have taken on a two-fronted attack: applications performance and analyser usability. The existing applications have continually demanded lower detection limits and higher specificity. New applications have strained the capabilities of the existing hardware. In parallel, the users have pushed to make the analysers more reliable, easy to use and adaptable to industrial information systems. This presentation addressed advances made on both fronts with the introduction of a new generation of process analytical liquid chromatographs. Advances made in the analyser design are helping to support demanding separation conditions, reach lower limits for trace determinations, and meet user needs for a more reliable, reproducible and configurable analyser.

### Near-infrared spectroscopy in on-line analysis of a xylene separation process

*Joel Bigman, Irena Zilberman and Ilan Sela, Petrometrix Ltd, P.O. Box 47, Migdal Ha'Emek, 10551, Israel*

The separation of *para*-xylene from a mixture of xylene isomers, in an aromatics extraction plant, is being monitored on-line, using NIR (Near-Infra Red) spectroscopic technology.

Specta and reference laboratory data of real process samples were used to create the calibration models, using chemometric methods.

The NIR instrument is of an innovative design that uses standard telecommunications optical fibres to separate the sample probe, located next to the process, from the analyser, which is located in the plant control room. The instrument is multiplexed to three sample probes in the plant. Two probes monitor the *para*-xylene separation process, while the third probe monitors an *ortho*-xylene stream.

A case study illustrated the effectiveness of this method during plant upsets, as well as during normal day-to-day operation.

### Application of on-line near infrared spectroscopy to extrusion processes of multicomponent polymer mixtures

*D. Fischer, Institute of Polymer Research, PF 120411, D-01005 Dresden, Germany*

Extrusion is one of the most important processes in plastic engineering. High demands on the quality of polymeric products and reducton of cost by avoidance of polymers of insufficient quality require fast and reliable monitoring methods. The use of near-infrared (NIR) spectroscopy on polymer melts opens up new possibilities of such process monitoring. The application of in-line NIR spectroscopy to extrusion processes of multicomponent polymer mixtures was the aim of our work. We used transmission spectroscopy for transparent melts and diffuse reflectance spectroscopy for opaque melts. The connection between the NIR probes and NIR spectrometer were realized through quartz fibres. The probes were adapted in-line at the end of an extruder.

The quantitative analysis of the polymer mixtures was carried out using chemometric methods, the Partial Least Squares method (PLS) and Principle Component Regression (PCR). Subjects of our investigations were the simultaneous quantification of the content of several additives and fillers in polymers, e.g. in poly(vinyl chloride) and polypropylene. We developed calibration models for all mixtures and applied the multiple scattering correction (MSC) for all diffuse reflectance spectra. The limit of detection and accuracy of the quantification for additives in polymers are dependent on the material and on the polymer used. Our results are between 0.1 and 1.5%.

An important role for the quantification of small amounts plays the optimization of the fibre accessory for the link between the spectrometer and NIR probe. It is furthermore taken into consideration that the investigated systems by using diffuse reflectance spectroscopy are blended nearly homogenous and that the calibration blends have the same scattering properties as the investigated real system. Our results showed, that in this manner, NIR spectroscopy with transmission and diffuse reflectance probes was suitable for quantitative in-line process analysis of multicomponent polymer mixtures.

### Automated off-line chelation sample pretreatment for complex matrices

*J. M. Riviello and A. Kirkland, Dionex Corporation, 1228 Titan Way, Sunnyvale, CA 94086, USA; P. Carpenter, ToxScan, 42 Hangar Way, Watsonville, CA 95076, USA*

The spectroscopic determination of trace metals in complex matrices, e.g. seawater, brines sludge and soils, is complicated by the presence of high concentrations of alkali and alkaline earth metals. Analysis of samples by inductively coupled plasma-mass spectrometry (ICP-MS), which contain dissolved solids above 0.5%, compromises detection limits due to physical and spectral interferences. One technique used to minimize these interferences involves the use of a chelating stationary phase which selectively retains trace elements of interest from the sample matrix.

In this presentation we described an automated sample pretreatment system which utilizes chelation chemistry. The system consisted of a high performance gradient pump, sampling pump and sampling valve to divert the flow to a chelation column, together with a liquid sampling system which supplies raw samples and collects the processed sample. This allows up to 42 samples, ranging in volume from 5 to 20 ml, to be processed automatically. The performance of the automated, off-line chelation sample pretreatment system was demonstrated using a seawater matrix and analysis by ICP-MS.

### Designing rugged automated SPE

*Lynn Jordan and Andrea Belec, Zymark Corporation, 68 Elm Street, Zymark Center, Hopkinton, MA 01748, USA*

When automation comes to mind, often people think of massive numbers of samples being processed unattended. The reality is that to accomplish this goal, the initial sample preparation, equipment and automated method must be rugged.

One of the most important aspects of any automated sample preparation involves designing the entire process to be rugged, from the sample handling before the samples are loaded onto the automated instrument, to the design of the automated instrument, through the method-automated instrument runs.

This paper focused on the automation of solid phase extraction (SPE) for biological samples using an automated SPE workstation. The talk covered the aspects of dealing with the inconsistencies present in biological samples, designing an automated procedure that can help to eliminate the effects of these variations, and designing of the equipment to help prevent issues from arising when working with biological samples.

### Reproducibility through automation for the determination of method 1664 with solid phase extraction

*Michael C. Janes, Horizon Technology, 8 Commerce Drive, Atkinson, NJ 03811, USA*

Solid Phase Extraction (SPE) has many advantages over Liquid Liquid Extraction (LLE) for the analysis of Oil and Grease Method 1664. Consistent and reproducible data are achieved when combining the benefits of SPE with the Horizon Technology SPE-DEX® extractor.

SPE reduces solvents, eliminates emulsions and produces quality data. The disk is eluted with 25 ml of hexane, a 78% solvent reduction from LLE. This environmentally friendly alternative reduces the time required for evaporation. Emulsions are eliminated, otherwise a common occurrence with LLE hexane. In many cases, SPE produces results closer to LLE freon than LLE hexane.

Performing extractions manually with SPE is labour intensive. Automation increases productivity, improves consistency and provides a safe work environment. Automating the procedure allows the analyst to accomplish multiple tasks while extractions are taking place. Multiple technicians can achieve higher precision due to the extraction consistency. Removing direct contact with solvents creates a safe environment. Finally, it minimizes the amount of glassware and reduces cleanup time.

This paper focused on the benefits of automating the SPE procedure. Precision and accuracy data were presented from chemical/petroleum companies, automotive industry and private laboratories. Recovery data comparing LLE and automated SPE showed the benefits of the system.

### Laboratory automation engineering, computer-aided optimization

*Jan E. van den Bosch, Philips Analytical X-Ray, Automation Business Unit, Lelyweg, 1, 7602 EA Almelo, The Netherlands*

Since serving a laboratory's customers is directly influenced by the laboratory performance in analysis speed and quality, especially for the automated industrial laboratory, the design and layout of the lab becomes critically important.

Accurate selection of equipment type and quantity, as well as the inter-linking dependence, determines the speed of sample throughput and analysis. Samples are sent to the laboratory in irregular intervals. Regardless of whether sample volume is low, steady or high, instruments still require maintenance/repair and actual sample throughput times are difficult to measure. In practice, this means that decisions for equipment set-up and connections within the laboratory are being made based on estimated guesses leading to unrealistic expectations regarding performance of the equipment, as well as performance of the laboratory once systems are installed and running.

By using modern (computer-aided) techniques, it is now possible to engineer the optimal laboratory design and layout. A computer program has been developed that simulates the complete automated laboratory and calculates actual sample throughput times within the laboratory (including airtube sending and receiving if applicable). The program calculations are based upon real-time sample arrival data, machine/instrument processing times and individual instrument characteristics, e.g. check/re-calibration, daily maintenance, breakdown, etc. Bottlenecks are easily identified by graphic visualization of sample processing where the entire process can be observed and evaluated. Deadlocks can be determined before they actually occur in the operation laboratory. This eliminates down-time once the system is installed and running. All events are logged and stored in a spreadsheet-readable format for further evaluation.

This report demonstrated the power of current and future laboratory automation, and reported on in-depth studies of the laboratory engineering technique.

### A switching system of twin samples for the analysis on-line by a gas chromatograph

*Yu Yingtao, Department of Applied Chemistry, Fushun Petroleum Institute, Liaoning, 113001, China*

In this paper, a novel auxiliary technology called 'the switching system of twin samples' (SSTS) is introduced, which can be of great use for the analysis on-line by a gas chromatograph.

The principal functions and advantages of SSTS can be generalized as follows:

By using this technology, the twin samples (i.e. a sample and its duplication) can be obtained at the same time and then analysed in turn. Thus, it can be convenient for the analyst to examine the repeatability and stability of the instruments used for analysis. Alternatively, during a reaction, if the analysis of one sample is unsuccessful or the result is abnormal, the duplication can be kept for a second analysis, without the whole reaction and following analyses on-line intermitted.

On the other hand, the quantity of one sample can be different from that of its duplication by using the sampling tubes of different sizes, respectively. Furthermore, each of the twin samples can be switched into either column in a gas chromatograph. If one kind of carrier gas, one column, one detector etc. are not efficient for the analysis of the same components in a sample, two sets of operating conditions can be used for the analyses of the twin samples, respectively. In other words, the nature of the adsorbents, inert solid supports, carrier gases, dimensions of columns, detectors and even the flow rates of the carrier gases can be different from each other in the analyses of the twin samples. Therefore, more information on the sample can be obtained.

In addition, the twin samples from different points on-line can be obtained alternately, or even at the same time, with the above-mentioned advantages maintained. If necessary, more than one duplication of one sample can be obtained at the same time and then analysed one by one. In other words, SSTS can undoubtedly bring convenience to an analyst and be of great use for many gas chromatographic methods. The principle and usage of the SSTS were also described in detail.

